# Using mutability landscapes of a promiscuous tautomerase to guide the engineering of enantioselective Michaelases

**DOI:** 10.1038/ncomms10911

**Published:** 2016-03-08

**Authors:** Jan-Ytzen van der Meer, Harshwardhan Poddar, Bert-Jan Baas, Yufeng Miao, Mehran Rahimi, Andreas Kunzendorf, Ronald van Merkerk, Pieter G. Tepper, Edzard M. Geertsema, Andy-Mark W. H. Thunnissen, Wim J. Quax, Gerrit J. Poelarends

**Affiliations:** 1 Department of Pharmaceutical Biology, Groningen Research Institute of Pharmacy, University of Groningen, Antonius Deusinglaan 1, Groningen 9713 AV, The Netherlands; 2 Department of Biophysical Chemistry, Groningen Biomolecular Sciences and Biotechnology Institute, University of Groningen, Nijenborgh 7, Groningen 9747 AG, The Netherlands

## Abstract

The Michael-type addition reaction is widely used in organic synthesis for carbon–carbon bond formation. However, biocatalytic methodologies for this type of reaction are scarce, which is related to the fact that enzymes naturally catalysing carbon–carbon bond-forming Michael-type additions are rare. A promising template to develop new biocatalysts for carbon–carbon bond formation is the enzyme 4-oxalocrotonate tautomerase, which exhibits promiscuous Michael-type addition activity. Here we present mutability landscapes for the expression, tautomerase and Michael-type addition activities, and enantioselectivity of 4-oxalocrotonate tautomerase. These maps of neutral, beneficial and detrimental amino acids for each residue position and enzyme property provide detailed insight into sequence–function relationships. This offers exciting opportunities for enzyme engineering, which is illustrated by the redesign of 4-oxalocrotonate tautomerase into two enantiocomplementary ‘Michaelases'. These ‘Michaelases' catalyse the asymmetric addition of acetaldehyde to various nitroolefins, providing access to both enantiomers of γ-nitroaldehydes, which are important precursors for pharmaceutically active γ-aminobutyric acid derivatives.

## Abstract (web summary)

The Michael-type addition reaction is used for carbon-carbon bond formation; however biocatalytic methods for this reaction are rare. Here, the authors generate and exploit mutability landscapes of 4-oxalocrotonate tautomerase to direct the redesign of this promiscuous enzyme into enantio-complementary Michaelases.

## Graphical abstract

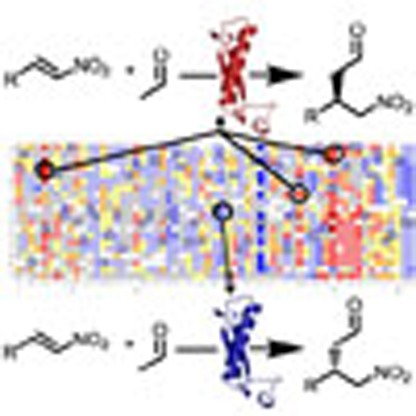

Chiral γ-nitroaldehydes are valuable precursors for pharmaceutically active GABA (γ-aminobutyric acid) derivatives. For example, marketed pharmaceuticals Phenibut (anxiolytic), Pregabalin (anticonvulsant), Baclofen (muscle relaxant) and Rolipram (antidepressant) can be readily obtained from distinct chiral γ-nitroaldehydes by two simple chemical steps^1^,^2^. Preparation of the appropriate γ-nitroaldehyde precursors for these GABA derivatives would require the Michael-type addition of acetaldehyde to diverse nitroalkene acceptors. The Michael-type addition of unmodified aldehydes to nitroalkenes has recently become viable by the development of proline- and peptide-based organocatalysts^3^,^4^,^5^. However, examples including acetaldehyde as donor substrate are scarce and 10–20 mol% of organocatalyst in organic solvent is typically applied^4^,^5^,^6^,^7^,^8^,^9^. Therefore, there is great interest in the development of alternative biocatalytic procedures for the enantioselective synthesis of γ-nitroaldehydes. Unfortunately, enzymes that naturally catalyse carbon–carbon bond-forming Michael-type additions are extremely rare^10^. A few elegant studies on promiscuous enzyme-catalysed carbon–carbon bond-forming Michael-type additions have been reported; however, most of these reactions proceed in organic solvent with low or moderate stereocontrol and do not involve acetaldehyde as donor substrate^11^,^12^,^13^,^14^,^15^,^16^,^17^,^18^.

The enzyme 4-oxalocrotonate tautomerase (4-OT) is composed of six identical subunits of only 62 amino acid residues each^19^,^20^. It belongs to the tautomerase superfamily, a group of homologous proteins that share a unique catalytic amino-terminal proline (Pro-1) and a characteristic β-α-β structural fold^21^,^22^,^23^. 4-OT takes part in a catabolic pathway for aromatic hydrocarbons in *Pseudomonas putida* mt-2 (Fig. 1a), where it catalyses the tautomerization of 2-hydroxymuconate (**1**) to yield 2-oxohex-3-enedioate (**2**)^24^. Residue Pro-1 functions as a general base (p*K*_*a*_≈6.4) that transfers the 2-hydroxyl proton of **1** to the C5-position to give **2** (ref. 25). Two other key catalytic residues are Arg-11 and Arg-39. Arg-39 is proposed to interact with the 2-hydroxyl group of **1** and a C-1 carboxylate oxygen, whereas Arg-11 is proposed to interact with the C-6 carboxylate group in a bidentate manner^26^. The latter interaction may draw electron density towards C-5 to facilitate protonation by Pro-1 (refs 21, 26, 27).

Inspired by the versatile success of proline and its derivatives as organocatalysts, we anticipated and found the proline-based enzyme 4-OT to promiscuously catalyse carbon–carbon bond-forming reactions such as aldolizations and Michael-type additions^28^,^29^,^30^,^31^. These include Michael-type additions of linear aldehydes, such as acetaldehyde (**3**) or butanal (**4**) (Fig. 1b), to a variety of nitroalkenes (**5a**–**5g**) to yield enantioenriched γ-nitroaldehydes (**6a**–**6g** and **7**), which are important precursors for GABA analogues such as the above-mentioned pharmaceuticals^31^. The proposed catalytic mechanism involves formation of an enamine intermediate of the aldehyde donor with the active site Pro-1 residue of 4-OT, reminiscent of the mechanism of proline-based organocatalysts^28^,^32^.

The discovery of the promiscuous Michael-type addition activity of 4-OT is an important step on the way to develop new biocatalysts for synthetically useful Michael-type reactions. Accordingly, it would be very attractive to enhance the Michael-type addition activity of 4-OT and improve its enantioselectivity. Because of the strong advantage of working with a protein with a small monomer size, we describe herein the construction of entire mutability landscapes for the expression, tautomerase and Michael-type addition activities, and enantioselectivity of 4-OT. These landscapes may be assessed and reasoned about as a whole, providing detailed insight into sequence–function relationships. Simultaneously, intelligent mutability-landscape navigation, coupled to combinatorial mutagenesis, offers exciting opportunities for rapid enzyme engineering^33^,^34^. This is illustrated here by the generation of 4-OT variants with improved activity and enantioselectivity, as well as inverted enantioselectivity, in Michael-type additions. The usefulness of these designer ‘Michaelases' is demonstrated by their application in the asymmetric addition of acetaldehyde to various nitroalkenes, providing convenient access to both enantiomers of chiral γ-nitroaldehydes, which are valuable precursors for GABA derivatives, several of which represent marketed pharmaceuticals.

## Results

### Collection of single 4-OT mutants

To chart the mutability landscapes of 4-OT, a collection of 4-OT genes encoding nearly all possible single variants of 4-OT was constructed. This unique collection covered at least 15 of the 19 possible variants at each residue position, from Ile-2 to the carboxy-terminal Arg-62. Single mutants of the N-terminal proline residue (Pro-1) were not included in the collection, because Pro-1 is a key catalytic residue and mutations at this position lead to incorrect demethionylation of the protein^35^,^36^. The 4-OT genes did not contain any affinity tag and were individually cloned, stored and expressed in *Escherichia coli* BL21(DE3). Importantly, as all 4-OT variants are stored and analysed separately, no oversampling is required.

### Mutability landscape of 4-OT for protein expression

A mutability landscape for protein expression was generated by determining the effect of each mutation on the production of soluble 4-OT protein. From the alignment of this landscape with the secondary structure elements of 4-OT (Fig. 2), it becomes apparent that many mutations in the α_1_-helix result in decreased production of soluble protein. Most notably, the introduction of a proline residue at any given position in the α_1_-helix has a pronounced effect on soluble 4-OT production. It seems likely to be that the introduction of a proline disrupts this α_1_-helix, resulting in an insoluble 4-OT mutant. The β-hairpin element (Gly-51 to Leu-56) is thought to play a crucial role in the stabilization of the hexameric structure of 4-OT^20^,^21^. Although most mutations in this region do not seem to affect the amount of soluble protein produced, replacing the negatively charged Glu-55 with a positively charged residue (Lys or Arg) decreases the amount of soluble 4-OT protein produced to a level that is below the detection limit. The average effect of each amino acid substitution on the amount of soluble protein produced reveals that substitutions to Trp, Arg and Pro are the most unfavourable ones (Fig. 2b). A probable explanation for this is that these residues have the biggest impact on the protein structure because of their volumetrically large side chain, charge or conformational rigidity, respectively. The finding that these amino acid substitutions are highly unfavourable for soluble protein production is consistent with the results of a recently published systematic mutagenesis study on a protein-binding domain^37^. On the other hand, some mutations seem to increase the amount of soluble protein produced. These mutations can be found mainly at positions His-6, Ile-7 and Lys-47. Gratifyingly, the majority of the mutants was produced in sufficient amounts to enable activity assays.

### Mutability landscape of 4-OT for tautomerase activity

**Figure 1:**
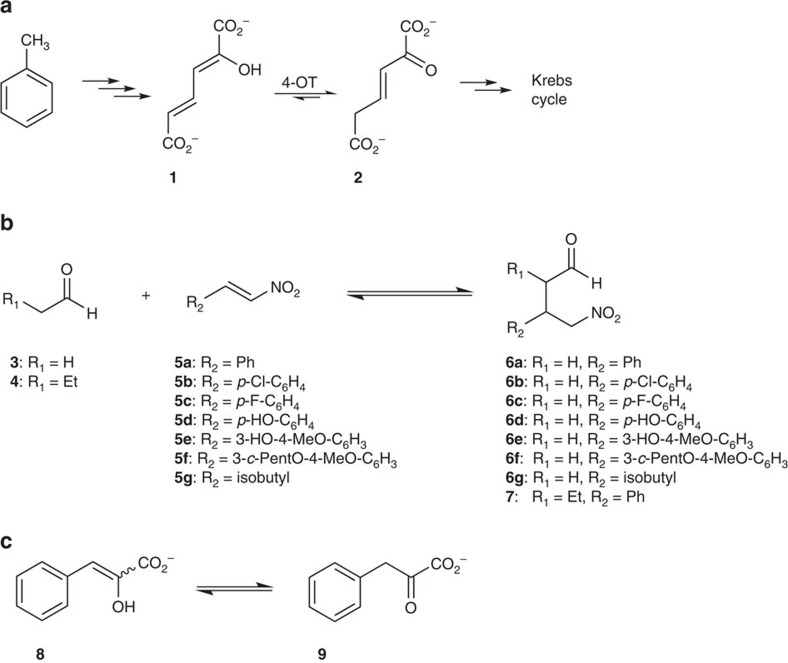
Natural and promiscuous reactions catalysed by 4-OT. (**a**) 4-OT-catalysed tautomerization of 2-hydroxymuconate (**1**) to yield 2-oxohex-3-enedioate (**2**) as part of a degradative pathway for aromatic compounds. (**b**) 4-OT-catalysed Michael-type additions of acetaldehyde (**3**) or butanal (**4**) to nitroalkenes **5a**–**5g** to give γ-nitroaldehydes **6a**–**6g** or **7**. (**c**) 4-OT-catalysed tautomerization of phenylenolpyruvate (**8**) to yield phenylpyruvate (**9**).

A mutability landscape for the tautomerase activity of 4-OT was generated by determining the effect of each mutation on the ability of the enzyme to ketonize phenylenolpyruvate (**8**) to phenylpyruvate (**9**) (Fig. 1c). Screening assays with the native substrate of 4-OT, 2-hydroxymuconate (**1**), were not possible due to the high activity (*k*_cat_∼3500, s^−1^) of the enzyme with this substrate^38^. Hence, activity profiling required very low enzyme concentrations, probably leading to hexamer dissociation, yielding unreproducible results. Therefore, the alternative substrate **8** (*k*_cat_=73 s^−1^) was used to monitor the tautomerase activity of 4-OT, which enabled a practical screening assay with reproducible results^39^.

**Figure 2:**
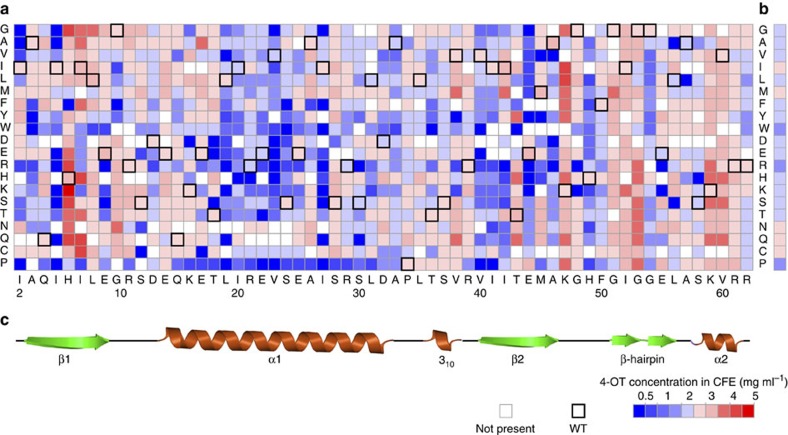
Mutability landscape of 4-OT for protein expression. (**a**) The horizontal axis of the data matrix depicts the wild-type sequence of 4-OT and the vertical axis depicts the 20 possible amino acid residues. The wild-type amino acid residue at each position is indicated by bold squares and white boxes represent mutants that are not present in the collection. The colour indicates the concentration of soluble 4-OT variants in CFE in mg ml^−1^, which was determined by quantitative densitometry on SDS–PAGE gels (Supplementary Fig. 1; details on this procedure can be found in the Methods section). The detection limit of this method is 0.5 mg ml^−1^. The depicted data are an average of two separate experiments (*n*=2). (**b**) The average effect of each amino acid substitution across the entire protein on the expression of soluble protein. (**c**) The secondary-structure elements of 4-OT^20^.

**Figure 3:**
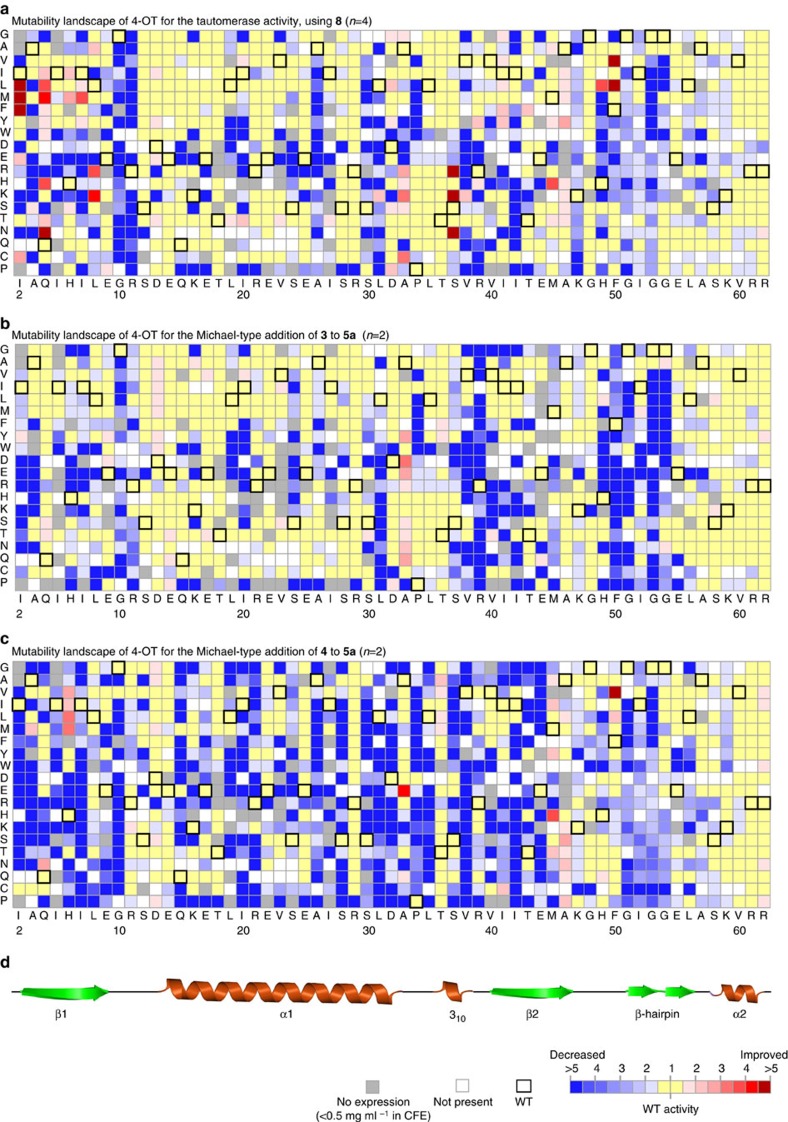
Mutability landscapes of 4-OT for tautomerase and promiscuous ‘Michaelase' activities. The horizontal axes of the data matrices depict the wild-type sequence of 4-OT and the vertical axes depict the 20 possible amino acid residues. The wild-type amino acid residue at each position is indicated by bold squares. Grey boxes represent mutants that were not produced above the detection limit (0.5 mg ml^−1^ in the CFE) and white boxes represent mutants that were not present in the collection. The colour indicates the specific activity of each mutant relative to that of wild-type 4-OT ((*U*_mut_ × mg^−1^)/(*U*_wt_ × mg^−1^)). All activities were determined using ultraviolet spectroscopy (details can be found in the Methods section) and enzyme concentrations in CFE were individually determined for each 4-OT variant using quantitative densitometric analysis of SDS gels. Each screening plate contained samples of wild-type 4-OT of which the average specific activity was used to calculate the relative specific activities of all 4-OT variants on that same plate. (**a**) The mutability landscape of 4-OT for tautomerase activity using substrate **8** (*n*=4). (**b**) The mutability landscape of 4-OT for the Michael-type addition of **3** to **5a** (*n*=2). (**c**) The mutability landscape of 4-OT for the Michael-type addition of **4** to **5a** (*n*=2). (**d**) The secondary-structure elements of 4-OT^20^.

The mutability landscape for 4-OT's tautomerase activity demonstrates the mutational robustness of the enzyme for this activity (Fig. 3a). This is apparent from mutations in various regions of the enzyme that show no or little effect on activity, including the regions Ser-12 to Arg-29 and Gly-54 to Arg-62. It has been hypothesized that such neutral mutations can have an important role in natural enzyme evolution, because they may result in ‘neutral drift'^40^,^41^,^42^. Whitman and colleagues^26^,^27^ demonstrated that the two active-site arginines (Arg-11 and Arg-39) of 4-OT are required for the tautomerization of dicarboxylate substrates, because they interact with the carboxylate moieties of the substrate. Fully consistent with this finding, we observed that only one active-site arginine (Arg-11) is essential for the tautomerization of the monocarboxylate substrate **8**. In addition to Arg-11, also Gly-10 appears to be important for the tautomerase activity. As mutations at Gly-10 have no significant effect on enzyme expression (Fig. 2), it is unlikely to be that this residue is required for correct overall folding of the enzyme. It is therefore tempting to speculate that this residue is required for securing the correct architecture of the active site.

Interestingly, five positions at which single mutations result in significantly improved tautomerase activity (>5-fold) were identified. These include mutations at positions Ile-2, Gln-4, Leu-8, Ser-37 and Phe-50. Whereas Ile-2, Leu-8, Ser-37 and Phe-50 are lining the Pro-1 pocket, the distance between Gln-4 and Pro-1 is ∼11 Å, illustrating that both close and distant mutations can improve activity^43^. These findings suggest that 4-OT could be readily engineered to become a more efficient catalyst for the tautomerization of **8**.

### Mutability landscape of 4-OT for the addition of **3** to **5a**

To generate a mutability landscape for the promiscuous ‘Michaelase' activity of 4-OT, the effect of each mutation on the ability of the enzyme to catalyse the addition of acetaldehyde (**3**) to *trans*-β-nitrostyrene (**5a**) was determined. This activity was monitored (using ultraviolet spectroscopy) by following the depletion of substrate **5a** in the presence of **3** and enzyme. For each mutant, control experiments confirmed that the depletion of **5a** was dependent on the presence of both **3** and enzyme. This indicates that the conversion of **5a** was indeed the result of an enzyme-catalysed Michael-type addition of **3** to **5a** (rather than the enzyme-catalysed addition of water to **5a**).

The mutability landscape shows that the majority of mutations does not have a significant effect on this ‘Michaelase' activity of 4-OT (Fig. 3b). This demonstrates the mutational robustness of the enzyme for this promiscuous activity. The key functionally important residue that can be identified from the mutability landscape is Arg-39, which is in accordance with the proposed mechanism of this Michael-type addition reaction, in which Arg-39 serves as a catalytic acid^29^. The observation that the activity is partially restored when Arg-39 is replaced by a positively charged residue that could function also as an acid (Lys or His) further strengthens this proposed mechanistic role of Arg-39. Interestingly, the other active-site arginine (Arg-11) appears to be less important for this promiscuous ‘Michaelase' activity (Fig. 3b), whereas it was shown to be essential for the phenylenolpyruvate tautomerase activity of 4-OT (Fig. 3a). Residues around and in the β-hairpin element (Gly-51 to Leu-56) also appear to be important for this Michael-type addition activity of 4-OT. Especially mutations at the positions of Phe-50 and the two glycine residues in the middle of the β-hairpin seem to have a large functional effect.

Most notably, four mutations at position Ala-33 result in an improved ‘Michaelase' activity of 4-OT. When this residue was replaced by negatively charged residues (Asp or Glu), or the corresponding polar residues (Asn or Gln), the activity was significantly enhanced. Of these mutants, variant A33D had a 3.5-fold increase in specific activity, making it the most active single 4-OT mutant for this Michael-type addition reaction. In contrast, the phenylenolpyruvate tautomerase activity of variant A33D was reduced ∼8-fold when compared with that of wild-type 4-OT.

**Figure 4:**
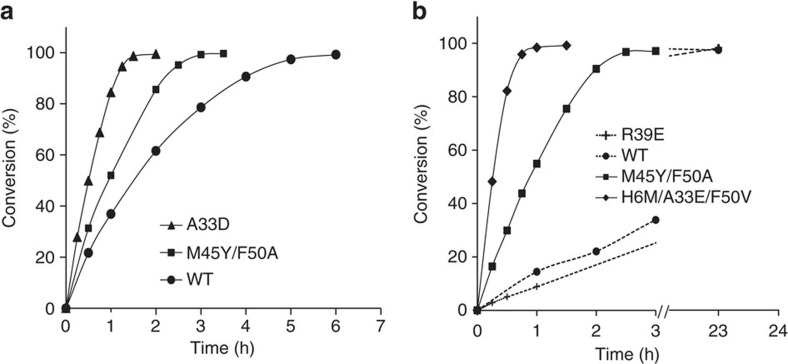
Enzyme-catalysed transformations. (**a**) Progress curves of the Michael-type addition of acetaldehyde **3** (50 mM) to *trans*-nitrostyrene **5a** (2 mM, 18 mg) catalysed (0.7 mol%) by wild-type 4-OT or 4-OT mutants A33D or M45Y/F50A. The reactions were carried out in buffer (20 mM NaH_2_PO_4_/10% ethanol (v/v)) at pH 7.3. (**b**) Progress curves of the Michael-type addition of butanal **4** (50 mM) to *trans*-nitrostyrene **5a** (2 mM, 18 mg) catalysed (0.7 mol%) by wild-type 4-OT or 4-OT mutants R39E, H6M/A33E/F50V or M45Y/F50A. The reactions were carried out in buffer (20 mM NaH_2_PO_4_/10% ethanol (v/v)) at pH 5.5. These progress curves are derived from the preparative scale reactions listed in Table 1.

The preparative usefulness of the A33D mutant was compared with that of wild-type 4-OT by transformations using 0.7 mol% biocatalyst and a 25-fold excess of **3** (50 mM) over **5a** (2 mM). Analysis of the progress curves of these reactions confirmed that mutant A33D is more efficient in the addition of **3** to **5a** than wild-type enzyme (Fig. 4a), with the A33D-catalysed reaction being completed within just 90 min at room temperature. The products were purified and characterized using ^1^H NMR spectroscopy, chiral phase high-performance liquid chromatography (HPLC) and optical rotation measurements (Supplementary Figs 2 and 3, and Supplementary Table 1). This analysis revealed that in addition to enhanced activity, mutant A33D also has improved enantioselectivity compared with wild-type 4-OT (Table 1, compare entries 1 and 2), producing the *3S* enantiomer of 4-nitro-3-phenylbutanal (**6a**) with an enantiomeric ratio (e.r.) of 99:1. The isolated product yield could be improved by conducting the enzymatic reaction at pH 6.5 instead of pH 7.3 (Table 1, entries 4 and 5, and Supplementary Figs 4–6), lowering the amount of byproducts formed, which result from the inherent tendency of acetaldehyde to rapidly react with itself, forming oligomers^44^. This makes the A33D mutant a promising biocatalyst for application in the asymmetric synthesis of enantiopure *3S*-**6a**.

### Synthetic usefulness of the A33D mutant enzyme

To further demonstrate the synthetic usefulness of the A33D mutant enzyme, we used this biocatalyst in the Michael-type addition of acetaldehyde (**3**) to a series of aromatic and aliphatic nitroolefin acceptors (**5b**–**5g**; Table 2). Nitroalkene (2–5 mM), acetaldehyde (50–150 mM) and A33D enzyme (0.5–5.3 mol%) were incubated in an appropriate solvent system, and reactions were followed by ultraviolet spectroscopy (Supplementary Figs 9–14). After complete conversion of the respective nitroalkene, standard workup and purification procedures were carried out, which yielded γ-nitroaldehydes **6b**–**6g** as confirmed by ^1^H NMR spectroscopy (Supplementary Figs 15–20). Analysis of products **6b**–**6g** by HPLC or gas chromatography (GC) on a chiral stationary phase (Supplementary Figs 21–26) revealed that the A33D-catalysed Michael-type addition reactions are highly enantioselective, producing these γ-nitroaldehydes with excellent e.r. values between 95:5 and >99:1 (Table 2). These results clearly demonstrate the potential of the A33D mutant enzyme for application in enantioselective synthesis of various γ-nitroaldehydes. The discovery of the A33D mutant illustrates the opportunities for protein improvement afforded by experimental protein mutability landscapes; this single mutant would probably not have been generated by using an error-prone PCR approach, because the codon for Ala-33 in 4-OT (GCG) requires two point mutations to produce an aspartate (GAT or GAC).

### Mutability landscapes of 4-OT for the addition of **4** to **5a**

To gain further insight into which residues govern the activity and enantioselectivity of 4-OT in Michael-type addition reactions, we determined the effect of each mutation on the ability of 4-OT to catalyse the addition of butanal (**4**) to *trans*-β-nitrostyrene (**5a**), and to yield enantioenriched product **7**. The addition of **4** to **5a** was chosen as a model reaction for determining the mutational effect on stereoselectivity, because the diastereo- and enantiopurity of product **7** can be determined directly by HPLC on a chiral stationary phase (Supplementary Fig. 27 and Supplementary Table 2), whereas the separation of the enantiomers of product **6a** (from the enzymatic addition of **3** to **5a**) by chiral-phase HPLC requires derivatization^29^,^30^. To prevent epimerization of product **7** and minimize the formation of side products, reactions were performed at pH 5.5 instead of pH 7.3 (ref. 30). Under these conditions, however, slight protein precipitation was observed with some mutants, precluding accurate activity measurements.

Nevertheless, the mutability landscape clearly demonstrates the mutational robustness of the C-terminal domain (Ala-57 to Arg-62) of 4-OT for the Michael-type addition of **4** to **5a** (Fig. 3c). This robustness of the C-terminal domain has also been observed for the other two activities analysed (Fig. 3a,b), which may suggest a minor contribution of this domain to catalysis. The overall mutational robustness of 4-OT for the Michael-type addition of **4** to **5a**, however, seems less when compared with the Michael-type addition activity of **3** to **5a**. This may be related to the difference in the pH of the assay buffer (pH 5.5 versus pH 7.3).

Importantly, the mutability landscape (Fig. 3c) revealed four residue positions at which mutations significantly improved the specific activity of 4-OT for the Michael-type addition of **4** to **5a**. The higher number of favourable mutations found for this reaction, when compared with the Michael-type addition of **3** to **5a**, could be the result of the additional ethyl group on the aldehyde substrate, which could serve as an extra handle for the enzyme to coordinate substrate binding. The first position where beneficial single mutations (Val, Ile, Leu or Met) were found is His-6. For this position, the best mutant enzyme was H6M, which has an ∼3-fold increased specific activity compared with that of wild-type 4-OT. The second position is Ala-33, which on mutation to a Glu improved the specific activity ∼4-fold compared with that of wild-type 4-OT (a Gln at this position improved the activity slightly). Notably, the A33E mutation also significantly improved the activity of 4-OT for the Michael-type addition of **3** to **5a** (Fig. 3b). The third position is Met-45; at this position, four single mutations improved the activity, including mutations to Ile (2.1-fold), Tyr (2.3-fold), His (3.6-fold) and Thr (2-fold). Finally, at position Phe-50, mutations to Leu and Val improved the specific activity 2.5- and ∼5-fold, respectively, compared with that of wild-type 4-OT. This makes the F50V enzyme the most active single 4-OT mutant for the Michael-type addition of **4** to **5a** (Supplementary Fig. 28 and Supplementary Table 3). These identified hotspots are good targets to further enhance 4-OT's promiscuous ‘Michaelase' activity, as shown by combinatorial mutagenesis, which yielded a triple mutant, H6M/A33E/F50V, with strongly enhanced activity for the Michael-type addition of **4** to **5a** (Fig. 4b, Table 1 and also see Supplementary Discussion for full details).

**Figure 5:**
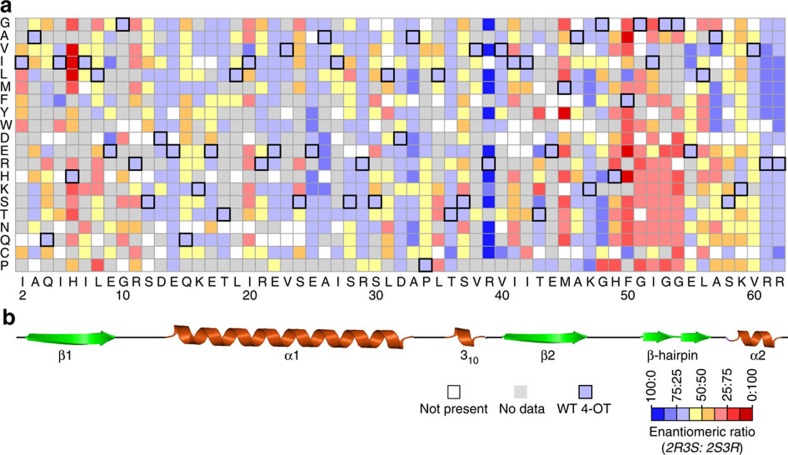
Mutability landscape of 4-OT for enantioselectivity in the Michael-type addition of **4** to **5a**. (**a**) The horizontal axis of the data matrix depicts the wild-type sequence of 4-OT and the vertical axis depicts the 20 possible amino acid residues. The wild-type amino acid residue at each position is indicated by bold squares and white boxes represent mutants that are not present in the collection. The colour indicates the e.r. (*2R3S*:*2S3R*) of the major *syn* diastereoisomer of **7** as produced by the 4-OT variants. The e.r. values were determined by HPLC on a chiral stationary phase. Grey boxes represent mutants that did not produce sufficient amounts of **7** to give an adequate ultraviolet signal during HPLC analysis. As expected, these grey boxes mainly coincide with the grey and dark blue boxes in Fig. 3c, which indicate mutants that either did not express or lack significant ‘Michaelase' activity. (**b**) The secondary-structure elements of 4-OT^20^.

No significant changes in the diastereoselectivity were observed, as all active mutant enzymes such as wild-type 4-OT produced the *syn* diastereomer of **7** in large excess (diastereomeric ratio (d.r.) ≥72:28). This observation is consistent with the postulated topological rule that explains the *syn*-selectivity in diastereoselective Michael-type additions of enamines to nitroalkenes^45^. Wild-type 4-OT has enantiopreference towards the *2R3S* enantiomer of the *syn* diastereoisomer, producing **7** with an e.r. of 57:43 (*2R3S:2S3R*) (Table 1, entry 7). The effect of each mutation on the ability of 4-OT to produce enantioenriched product **7** is shown in Fig. 5. The mutability landscape shows that many single mutations have a significant effect on 4-OT's enantioselectivity. The residue positions where single mutations led to the most pronounced improvement in enantioselectivity are Ala-33, Arg-39, Ala-57 and Arg-61. A comparison of the mutability landscapes suggests that in contrast to the mutational robustness of the C-terminal domain for the natural and promiscuous activities of 4-OT, this domain appears to contribute largely to the enantioselectivity of 4-OT in the Michael-type addition of **4** to **5a**.

The single mutants with the best enantioselectivity at each of these residue positions (A33D, R39E, A57Y and R61M) were purified and characterized for their ability to produce enantioenriched products in the Michael-type addition of **4** to **5a**, as well as **3** to **5a** (Supplementary Table 4). Fully consistent with the mutability landscape shown in Fig. 5, the enzyme R39E has the highest enantioselectivity for the Michael-type addition of **4** to **5a**, producing γ-nitroaldehyde **7** with an e.r. of 94:6 (*2R3S:2S3R*). Although mutant R39E also displayed significantly improved enantioselectivity in the Michael-type addition of **3** to **5a**, producing *3S*-**6a** with an e.r. of 96:4 (Supplementary Table 4), mutant A33D has the highest enantioselectivity in this reaction (*vide supra*).

The preparative usefulness of the R39E mutant was compared with that of wild-type 4-OT by transformations using 0.7 mol% biocatalyst and a 25-fold excess of **4** (50 mM) over **5a** (2 mM). Analysis of the progress curves of these reactions showed that R39E exhibits similar activity compared with WT 4-OT (Fig. 4b). However, in contrast to wild-type 4-OT, R39E afforded highly enantioenriched product **7** with an e.r. of 95:5 (Table 1, compare entries 7 and 9). This makes the R39E mutant a promising biocatalyst for application in the asymmetric synthesis of enantioenriched *2R3S*-**7**.

Interestingly, the mutability landscape (Fig. 5) further shows that several single variants of 4-OT have inverted enantioselectivity, producing the *2S3R* enantiomer of **7** in excess over the *2R3S* enantiomer. Mutations at positions His-6, Arg-11, Met-45, Phe-50 and Gly-54 had the most pronounced effect on inversion of enantioselectivity. Three of these positions (His-6, Met-45 and Phe-50) overlap with the positions at which single mutations were identified, which improve 4-OT's ‘Michaelase' activity for addition of **4** to **5a** (Fig. 3c). The best mutant at each of these positions (H6I, R11I, M45H, F50A and G54E) was purified and characterized for its ability to produce enantioenriched products in the Michael-type addition of both **3** to **5a** and **4** to **5a**. At position Met-45, the second-best mutant was chosen for analysis, because the best mutant at this position (M45Y) could not be purified to homogeneity due to insufficient expression (consistent with the mutability landscape shown in Fig. 2a). Strikingly, all five mutants did not only have inverted enantioselectivity for the Michael-type addition of **4** to **5a** but also for the Michael-type addition of **3** to **5a** (Supplementary Table 5). Of these mutants, the enzyme F50A has the highest enantioselectivity producing both *3R-***6a** and *2S3R-***7** with an e.r. of 93:7, demonstrating its suitability for the preparation of the opposite enantiomers (as compared with those produced in the same reactions catalysed by A33D, R39E or wild-type 4-OT) of γ-nitroaldehydes **6a** and **7**.

### Engineering 4-OT variants with inverted enantioselectivity

To further improve the selectivity of 4-OT towards the *2S3R* enantiomer of **7**, we generated double and triple mutants based on all possible combinations of the best single 4-OT mutants (that is, H6I, M45H or M45Y, and F50A). Using analytical scale assays, these double- and triple-mutant enzymes were evaluated for their enantioselectivity in the Michael-type addition of **4** to **5a**, yielding **7**. Three (H6I/M45H, H6I/M45Y and H6I/M45H/F50A) of the seven constructed mutant enzymes displayed either low or no detectable ‘Michaelase' activity. Gratifyingly, the other four mutants (H6I/F50A, M45Y/F50A, M45H/F50A and H6I/M45Y/F50A) displayed respectable activity and produced **7** with an e.r. >96:4 (*2S3R:2R3S*) (Supplementary Table 6). This product enantiopurity is higher than that obtained with the best single mutant (F50A), indicating an additive effect of the mutations.

The preparative usefulness of the mutant with the best activity and enantioselectivity, M45Y/F50A, was analysed by transformations using 0.7 mol% biocatalyst and a 25-fold excess of aldehyde (**3** or **4**) over **5a**. The progress curves of these reactions demonstrate that this mutant, in addition to inverted enantioselectivity, also has significantly enhanced ‘Michaelase' activity for the addition of **3** to **5a** (Fig. 4a), as well as **4** to **5a** (Fig. 4b) when compared with wild-type 4-OT. The M45Y/F50A-catalysed Michael-type addition of **4** to **5a** gave *2S3R*-**7** in excellent yield (86%) and with high enantiopurity (96:4) (Table 1, entry 10). Interestingly, the M45Y/F50A mutant also displayed high enantioselectivity in the Michael-type addition of **3** to **5a**, yielding *3R***-6a** with an e.r. of 94:6 and a yield of 30% (Table 1, entry 3). The product yield could be improved to 65% by conducting the enzymatic reaction at pH 5.5 (Table 1, entry 6), which lowers the amount of side-products formed.

### Synthetic usefulness of the M45Y/F50A mutant enzyme

To further demonstrate the usefulness of the M45Y/F50A mutant for synthesis of the opposite enantiomers of γ-nitroaldehydes **6b**–**6g**, as compared with those afforded by mutant A33D (Table 2), we also used this engineered enzyme in the Michael-type addition of acetaldehyde (**3**) to the nitroolefin acceptors **5b**–**5g** (Table 3). After complete conversion of the respective nitroalkene (Supplementary Figs 9–14), standard workup and purification procedures were carried out, affording γ-nitroaldehydes **6b**–**6g** (Supplementary Figs 15–20). Analysis of these enzymatic products by chiral-phase HPLC or GC (Supplementary Figs 21–26) revealed that the M45Y/F50A-catalysed Michael-type addition reactions indeed yield the opposite enantiomers of γ-nitroaldehydes **6b**–**6g** with e.r. values between 62:38 and 97:3 (Table 3). These results clearly demonstrate the potential of the M45Y/F50A enzyme for application in the synthesis of enantioenriched *3R*-**6b-6f** and *3S*-**6g**, which are valuable precursors for pharmaceutically active GABA analogues, and further illustrate the exciting opportunities for enzyme engineering afforded by experimental protein mutability landscapes for enantioselectivity.

### Structural consequences of the M45Y and F50A mutations

**Figure 6:**
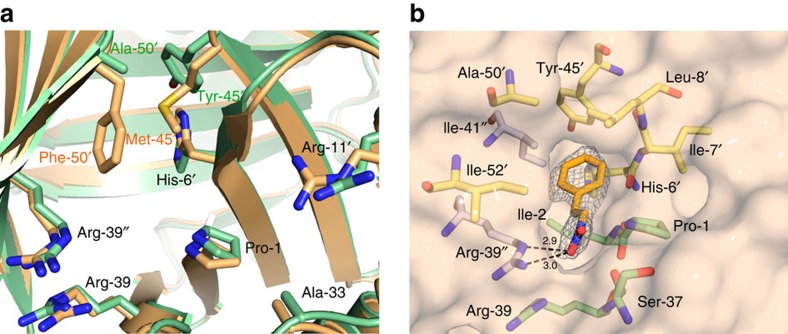
Structural characteristics of the M45Y/F50A mutant. (**a**) Superposition of the residues lining the hydrophobic Pro-1 pocket in wild-type 4-OT (orange) and the M45Y/F50A mutant (green). (**b**) Surface and stick representation of the Pro-1 pocket in the M45Y/F50A mutant, depicting the bound *trans*-β-nitrostyrene (orange) as observed in the crystal structure. The grey mesh shows the 2*F*_o_−*F*_c_ electron density contoured at 1.0*σ*. Residues from the neighbouring chains are shown in different colours and labelled with apostrophes. The hydrogen-bonding interactions (distances in Å) are shown as black dashed lines.

To obtain insight into the structural consequences of the M45Y and F50A mutations, we determined crystal structures of mutant M45Y/F50A in an unliganded state and in complex with substrate *trans*-β-nitrostyrene (**5a**) at 2.7 and 2.3 Å resolution, respectively (Supplementary Table 7 and Supplementary Fig. 29). The overall structure of the M45Y/F50A mutant did not reveal significant changes in backbone conformation compared with wild-type 4-OT. Hexameric Cα backbones of mutant and wild-type 4-OT superimpose with an average root-mean-square deviation of 0.32 Å (Supplementary Fig. 30). However, the M45Y and F50A mutations did have a pronounced effect on the active-site geometry near Pro-1 (Fig. 6a and Supplementary Fig. 31a,b). In the wild-type 4-OT structure, the side chains of Met-45 and Phe-50 point towards Pro-1, restricting access to a small hydrophobic pocket at the back of the active site. In the structure of mutant M45Y/F50A, the side chain of Tyr-45 is rotated away from Pro-1 towards the back of the active site, where it forms a hydrogen bond with His-6 (Supplementary Fig. 31c). Combined with the replacement of the bulky Phe-50 with an alanine, the M45Y mutation results in a significant opening and enlargement of the hydrophobic pocket near Pro-1 (Fig. 6a and Supplementary Fig. 31a,b). This enlarged pocket in the M45Y/F50A mutant is able to accommodate the phenyl group of **5a**, as revealed in the crystal structure of mutant M45Y/F50A complexed with this substrate (Fig. 6b). The phenyl group of **5a** makes van der Waals contacts with residues Pro-1, His-6, Ile-7, Leu-8 and Ile-41 in the hydrophobic pocket, whereas the nitro group is in van der Waals contact with Ser-37 and forms a salt bridge with the guanidinium group of Arg-39. The central ethylene group of **5a** is only ∼3 Å away from the amino group of Pro-1. The observed binding mode of **5a** in the structure of mutant M45Y/F50A is not possible in the wild-type 4-OT structure, due to steric hindrance by the Phe-50 and Met-45 residues.

In conclusion, our results reveal that the two mutations (M45Y and F50A) unexpectedly created a new substrate-binding pocket. This active-site remodelling is most probably responsible for the observed differences in activity and enantioselectivity between mutant and wild-type 4-OT. However, these findings *per se* do not explain the enantiopreference of mutant M45Y/F50A. The electron density for bound **5a** does not indicate a preference of this substrate to bind with an orientation relative to Pro-1 that is consistent with the formation of the (*R*)-product; the other orientation differing by a rotation of ∼180° around the longitudinal axis of the substrate molecule, and consistent with formation of the (*S*)-product, fits equally well (Supplementary Fig. 31d). In addition to highlighting that active-site remodelling may lead to inverted enantioselectivity, our crystallographic results provide an important guide for future engineering experiments.

## Discussion

In summary, we provide evidence that a catalytically promiscuous tautomerase is a good starting point to develop novel enantiocomplementary biocatalysts for unnatural Michael-type additions of acetaldehyde (or butanal) to various nitroalkenes. These enzyme-catalysed reactions provide ready access to both enantiomers of γ-nitroaldehydes, given that the product yields may be further improved by optimizing the reaction conditions and purification protocols. This new biocatalytic procedure provides an attractive complement to existing organocatalytic methodologies. Chiral γ-nitroaldehydes are valuable building blocks for GABA derivatives and their rapid synthesis should facilitate the development of new pharmaceuticals.

The applied systematic mutagenesis strategy allows the important discrimination between beneficial mutations and those that are neutral or detrimental, providing detailed insight into sequence–function relationships. The results provide support for the notion that the generation of large-scale mutational data, revealing the functional consequences of a great number of protein variants, may have important implications for understanding and engineering proteins^46^,^47^,^48^. Interestingly, the mutability landscapes of 4-OT demonstrate that in contrast to the mutational robustness of the C-terminal domain for 4-OT's different activities, this domain appears to contribute largely to the enantioselectivity of the enzyme in the Michael-type addition reaction. Hence, this domain may represent a good target for future mutagenesis experiments, with the aim to optimize the enantioselectivity of 4-OT in Michael-type additions using different combinations of aldehyde and nitroalkene. We also aim to exploit the large amount of mutational data generated within this study, which can be used as a unique training set, and machine learning algorithms to generate mutation prediction models for 4-OT as well as closely related tautomerase superfamily members.

## Methods

### Mutant collection preparation

To create mutability landscapes of 4-OT, a defined collection of 1,040 single mutant 4-OT genes was purchased from DNA2.0 (Menlo Park, CA). This collection covered at least 15 of the 19 possible variants on each amino acid residue position ranging from Ile-2 to the C-terminal Arg-62. In total, 90% of all possible single 4-OT mutants is present in this collection. Each mutant gene was individually cloned in a pJexpress 414 vector, sequenced and transformed into *E. coli* DH10B by DNA2.0. Each DH10B culture, which carried a mutated 4-OT gene in a pJexpress 414 vector, was separately grown in liquid lysogeny broth (LB) containing 100 μg ml^−1^ ampicillin (amp). All plasmids, each containing a single mutant 4-OT gene, were isolated from these cultures using the NucleoSpin 96 Plasmid Core Kit (Bioké, Leiden, NL). An aliquot of each isolated plasmid was transformed individually into chemically competent *E. coli* BL21 (DE3). The transformants were selected at 37 °C in liquid LB containing 100 μg ml^−1^ amp. Each *E. coli* BL21 (DE3) transformant harbouring a pJexpress 414 vector with a unique 4-OT gene was stored at −80 °C until further use.

### CFE production and enzyme concentration assessment

The expression levels, activities and enantioselectivities of all members of the 4-OT mutant collection were determined using cell-free extracts (CFEs) of cultures each expressing a different 4-OT mutant. To inoculate these cultures, aliquots of the −80 °C stock of the transformed *E. coli* BL21 (DE3) cells were placed into wells of a 2.2-ml 96-deep-well plate (Greiner Bio-one, 96-well Masterblock). Each mutant was placed into two wells as a duplicate. Each well contained 1.25 ml LB, supplemented with 100 μg ml^−1^ amp and 100 μM isopropyl-β-D-1-thiogalactopyranoside as an inducer. The deep-well plates with inoculated LB medium were sealed with sterile, gas-permeable seals (Greiner Bio-one, BREATHseal) and incubated overnight at 37 °C with shaking at 250 r.p.m. After the incubation, the cultures were pelleted at 3,500 r.p.m. for 30 min at 4 °C. The pellets of the duplicates were pooled and lysed with 375 μl BugBuster (Novagen), which was supplemented with 25 U ml^−1^ benzonase nuclease. The cell lysates were incubated with vigorous shaking at room temperature for 20 min. The cell lysate was cleared by centrifugation at 4,000 r.p.m. for 20 min at 4 °C, after which the CFE was obtained as the supernatant. To assess the 4-OT concentration in each CFE, 2 μl of each CFE was mixed with 18 μl of protein sample buffer (containing 9% (w/v) SDS, 40% (v/v) glycerol, 20% (v/v) β-mercaptoethanol, 0.1% (w/v) bromophenol blue and 250 mM Tris pH 6.8). Of the final mixture, which contained CFE and protein sample buffer, 10 μl was loaded on a 26-well pre-cast 10% polyacrylamide gel (NuPAGE Novex 10% Bis-Tris). Four samples with standard amounts of 0.5, 1, 2.5 and 5 μg of homogenous wild-type 4-OT were loaded on the gel besides the CFE samples and served as calibration samples for the quantification of the 4-OT mutant protein in the CFE. The purification method of wild-type 4-OT is described elsewhere^28^. The concentration of the purified wild-type 4-OT was determined by the Waddell method^49^. After electrophoresis (at 150 V for 1 h), the gels were stained using the Coomassie-based stain InstantBlue (Expedeon Ltd). A digital image of the gel was recorded using the Chemi Genius^2^ Bio Imaging System (Syngene, Cambridge, UK). A typical picture of an SDS-gel, which was used in this densitometric concentration assessment, is present in Supplementary Fig. 1. The concentrations of the 4-OT mutants in the CFE were quantified based on the size and intensity of the 4-OT bands on the digital image of the gel, relative to the 4-OT bands used for calibration. The calibration samples were present on each gel and were only used to quantify 4-OT concentrations of the samples that were loaded on that same gel. The size and intensity of the protein bands were quantified by using the software GeneTools (version 4.02, Syngene) and were used to make a calibration curve. The average *R*^2^ value of the 122 calibration curves, used to determine all expression levels, was 0.97. This indicates the accuracy of this quantification method. This concentration assessment preceded each activity screening of each CFE sample. The CFE was stored at room temperature (<5 h) until further use. The mutability landscape of 4-OT for the expression of soluble protein was generated using the data obtained from these concentration assessments. The mutability landscape was generated using Microsoft Excel 2010.

### Mutability landscapes of 4-OT for activity

All enzymatic activities of the 4-OT mutants were determined by ultraviolet spectroscopy. The ultraviolet-spectroscopic measurements were done in 96-well microtitre plates (MTPs) (UV-star μclear, Greiner Bio-one). The volume of the reaction mixtures in all wells was 100 μl and the temperature in the ultraviolet-plate reader was set to 25 °C for all analyses.

Mutability landscape of 4-OT for tautomerase activity using phenylenolpyruvate (**8**). The tautomerase activity of the 4-OT mutants was assessed by determining their ability to ketonize phenylenolpyruvate (**8**). The final reaction mixtures consisted of the following: CFE (0.2% v/v), phenylenolpyruvate (400 μM (two times *K*_m_ of wild-type 4-OT)^39^) and ethanol (10% v/v) in 10 mM NaH_2_PO_4_ buffer (pH 7.3). First, the CFE and buffer were added to each well of the MTP using a Packard Multiprobe II HT EX 8 tip robotic liquid handling system. The assay was initiated by the addition of 10 μl stock solution of phenylenolpyruvate (4.0 mM in ethanol) to each well of the MTP, which already contained the appropriate CFE and buffer mixture using the integrated reagent injector on the SPECTROstar Omega plate reader (BMG LABTECH, Isogen Life Science, de Meern, NL). After adding the substrate to one well, the plate was briefly shaken (2 s at 500 r.p.m.) before that well was analysed for 40 s at 283 nm with a 0.26-s data interval, following the depletion of **8**. After finishing the measurement in one well of the MTP, the substrate was added to the next well to initiate the assay in that well. These reaction conditions enabled accurate rate determination in the range of fivefold decreased to fivefold increased activity compared with wild-type 4-OT. This activity assay was done in quadruplicate.

Mutability landscape of 4-OT for the Michael-type addition of acetaldehyde (**3**) to *trans*-β-nitrostyrene (**5a**). The reaction mixtures used to monitor the 4-OT-catalysed Michael-type addition of **3** to **5a** consisted of the following: CFE (40% v/v), substrate **5a** (500 μM (two times *K*_m_ of wild-type 4-OT)^29^), substrate **3** (50 mM) and ethanol (5% v/v) in 10 mM NaH_2_PO_4_ buffer (pH 7.3). First, the appropriate amounts of CFE and buffer were added to each well of the MTP using a Packard Multiprobe II HT EX 8 tip robotic liquid handling system. The assay was initiated by adding 10 μl of a stock solution of **3** (500 mM in 10 mM NaH_2_PO_4_ buffer (pH 7.3)) and 5 μl of a stock solution of **5a** (10 mM in ethanol) to each well of the screening plate, which already contained the appropriate CFE and buffer mixture. To ensure proper mixing of the reagents, the plate was shaken (30 s at 500 r.p.m.) before the reaction rates were recorded by monitoring the depletion of **5a** at 320 nm (*λ*_max_ **5a**=320 nm) for 40 min with a 60-s data interval. To observe possible precipitation or other atypical changes in the reaction mixture, the ultraviolet spectrum (220–500 nm) of each well was recorded before and after the reaction. The activity assay was done in duplicate. Separate control experiments in which the depletion rate of **5a** was assessed in the presence of each 4-OT mutant, but in the absence of aldehyde, indicated that none of the 4-OTs in the mutant collection could catalyse the conversion of **5a** at a significant rate in the absence of **3**.

Mutability landscape of 4-OT for the Michael-type addition of butanal (**4**) to **5a**. The reaction mixture used to monitor the 4-OT-catalysed Michael-type addition of **4** to **5a** consisted of the following: CFE (20% v/v), substrate **4** (32.5 mM), substrate **5a** (650 μM) and ethanol (5% v/v) in 50 mM NaH_2_PO_4_ buffer (pH 5.5). First, the appropriate amounts of CFE and buffer were added to each well of the MTP using a Packard Multiprobe II HT EX 8 tip robotic liquid handling system. The assay was initiated by adding 32.5 μl of a stock solution of **4** (100 mM in 50 mM NaH_2_PO_4_ (pH 5.5)) and 5 μl of a stock solution of **5a** (13 mM in ethanol) to each well of the MTP, which already contained the appropriate CFE and buffer mixture. To minimize evaporation of solvent and aldehyde **4**, the MTPs were sealed with ultraviolet transparent plate seals (VIEWseal, Greiner Bio-one). To ensure proper mixing of the reagents, the plate was shaken (30 s at 500 r.p.m.) before the reaction rates were recorded by monitoring the depletion of **5a** at 320 nm for 80 min with a 60-s data interval. To observe possible precipitation or other atypical changes in the reaction mixture, the ultraviolet spectrum (220–500 nm) of each well was recorded before and after the reaction. The buffer pH of 5.5 was used to prevent epimerization of the product (**7**). The activity assay was done in duplicate.

Data analysis. The duplicates of the substrate depletion curves obtained in the activity assays were combined and an average substrate depletion curve was plotted. The initial substrate depletion rates were determined from the slopes of the linear section of these curves using MARS data analysis software (version 2.40 (BMG LABTECH, Isogen Life Science)). The background substrate depletion rates were determined separately for each assay by assessing the average initial rate of substrate depletion in reaction mixtures containing CFE of *E. coli* BL21 (DE3) cultures carrying an empty pJexpress 414 vector (EV). This background substrate depletion rate was subtracted from all other substrate depletion rates determined for 4-OT variants on that same MTP. When the resulting enzyme activity (U) was zero, the sample was marked accordingly. The earlier established enzyme concentrations in the CFEs were used to calculate the specific activities (U mg^−1^) of each mutant. When an enzyme concentration in the CFE was lower than 0.5 μg  μl^−1^ (that is, the detection limit), the sample was marked accordingly. An average of the wild-type 4-OT-specific activities was calculated for each MTP and all the other specific activities of the mutants on the same MTP were divided by this value to yield the relative specific activities ((*U*_mut_ × mg^−1^)/(*U*_wt_ × mg^−1^)) of all mutants. The EV and wild-type 4-OT controls were exclusively used for calculations of relative activities of mutants on that same MTP. The data were graphically represented in the mutability landscapes for activity, which were generated using Microsoft Excel 2010.

### Mutability landscape of 4-OT for enantioselectivity

Reverse-phase HPLC method development for chiral separation of stereoisomers of **7**. Racemic 2-ethyl-4-nitro-3-phenylbutanal (**7**) was prepared according to a literature procedure^30^. This racemic compound was used to test the ability of a Chiralpak ID column (150 mm × 4.6 mm, Daicel) to separate the *2R3S*-**7** and *2S3R*-**7** enantiomers, with the aim of establishing a rapid reverse-phase HPLC method that could be used to generate the mutability landscape of 4-OT for enantioselectivity. All four stereoisomers of **7** could be separated using this column with an isocratic mobile phase (MeCN:H_2_O 31:69) at a flow rate of 1.5 ml min^−1^ (Supplementary Fig. 27). The chromatogram was recorded using a diode array detector (210 or 220 nm). The absolute configuration of the *syn*-enantiomers was determined by injecting **7** that was enzymatically produced by wild-type 4-OT, which produces the *2R3S*-**7** enantiomer in excess^30^. The in-house HPLC apparatus consisted of the following components: Shimadzu LC-10 AT pump, a Shimadzu SIL-20A auto injector and a Shimadzu SPD-M10A VP diode array detector. The HPLC chromatographic data were analysed using data processing software (LC Solutions) obtained from Shimadzu.

Supplementary Fig. 27a demonstrates the chiral separation of racemic **7** using the above-mentioned HPLC conditions. As wild-type 4-OT has been reported to produce **7** with a d.r. of 89:11 (*syn:anti*) and an e.r. of 69:31 (*2R3S:2S3R*)^30^, the absolute configuration of the *syn-*enantiomers could be assigned based on the HPLC chromatogram of enzymatically produced **7** (Supplementary Fig. 27b and Supplementary Table 2). As the enantioselectivity of wild-type 4-OT is unknown for the anti-enantiomers, their absolute configuration cannot be assigned. Therefore, the peaks corresponding to the enantiomers of the anti-diastereoisomer are labelled as ‘anti-1' and ‘anti-2' based on the order of elution (Supplementary Fig. 27).

#### Generating the mutability landscape of 4-OT for enantioselectivity

The same reaction mixtures that were used to monitor the Michael-type addition of **4** to **5a** catalysed by the 4-OT variants were also used to evaluate the ability of each 4-OT variant to produce enantioenriched **7**. The duplicate reaction mixtures were pooled and filtered using 96-well ultrafiltration plates (AcroPrep Advance 96 Filter Plate 3 K Omega, Pall Life Sciences). The samples were passed through the filters by applying vacuum. The filtrate, which was now free of debris and protein, was analysed for enantioenriched **7** by reverse-phase chiral HPLC, based on the above-mentioned protocol. All four stereoisomers of the product **7** were separated using a Chiralpak ID column (150 mm × 4.6 mm, Daicel) as shown in Supplementary Fig. 27. To decrease the retention times, the flow rate was increased to 1.8 ml min^−1^ for the HPLC analyses used to generate the mutability landscape of 4-OT for enantioselectivity. The injection volume of the samples (that is, the filtered reaction mixtures) was 20 μl. The e.r. values were calculated based on the peak integrations, which were determined at 210 nm. This data was graphically represented in the mutability landscape for enantioselectivity, which was generated using Microsoft Excel 2010.

Analytical scale determination of the ability of purified 4-OT variants to produce enantioenriched **7**. To measure the ability of purified 4-OT enzymes to produce enantioenriched **7**, analytical scale experiments were conducted. The reaction mixtures for this consisted of the following: **4** (32.5 mM), **5a** (0.65 mM), 65 μM 4-OT (monomer concentration) and ethanol (5% v/v) in 20 mM NaH_2_PO_4_ buffer (pH 5.5); the final volume of this reaction mixture was 2 ml. The reaction was followed by monitoring the depletion of **5a** with ultraviolet spectroscopy at 320 nm. Aliquots of 200 μl were taken from this reaction mixture, cleared by ultrafiltration using Nanosep 3 K omega centrifugal filters (Pall Life Sciences) and analysed by chiral reverse-phase HPLC using a Chiralpak ID column as described above. The obtained e.r. values are depicted in Supplementary Tables 4 and 5.

Assessment of the ability of purified 4-OT variants to produce enantioenriched **6a**. The reaction conditions used to assess the ability of purified 4-OT variants to produce enantioenriched **6a** was based on a previously described method^30^. The reaction mixture consisted of the following: 4-OT (14.7 μM (0.7 mol%)), **3** (50 mM), **5a** (2.0 mM), and ethanol (10% v/v) in 20 mM NaH_2_PO_4_ buffer (pH 5.5). The reaction volume was 20 ml. Ultraviolet spectra (200–400 nm) were recorded at 30-min intervals to monitor the depletion of **5a**. The reaction was completed when the absorbance at 320 nm (*λ*_max_ **5a**=320 nm) vanished. The enzyme was removed from the reaction mixture by ultrafiltration (Vivaspin centrifugal concentrator (molecular weight cut off (MWCO) 5,000 Da)). The filtrate was collected and extracted with ethyl acetate (3 × 10 ml) using a separatory funnel. The organic phases were combined, dried with MgSO_4_ and evaporated *in vacuo*, yielding a yellowish oil. The product was dissolved in CDCl_3_ and a ^1^H NMR spectrum was taken to confirm the formation of **6a**. All ^1^H NMR spectra were recorded on a Bruker DRX-500 (500 MHz) spectrometer and were referenced to residual CHCl_3_ (*δ*=7.26). After confirming that product **6a** was obtained, it was converted to its corresponding ethylene glycol acetal according to a literature procedure^29^. The e.r. of the ethylene glycol acetal of **6a** was determined based on a literature procedure using reverse-phase HPLC with a Chiralpak AD-RH column (150 mm × 4.6 mm, Daicel) and an isocratic mobile phase (MeCN:H_2_O 70/30) at 0.5 ml min^−1^ (ref. 30). The obtained e.r. values are depicted in Supplementary Tables 4 and 5.

### Combinatorial mutagenesis of ‘hotspots'

Construction and screening of the focused library to further improve the Michael-type addition (**4** to **5a**) activity of 4-OT. The mutability landscape revealed four residue positions at which mutations improved the activity of 4-OT for the Michael-type addition of **4** to **5a**. To further improve this activity, a 4-OT mutant library was constructed in which the most favourable mutations at these four positions were combined. The degeneracy at the positions His-6, Ala-33 and Phe-50 was limited to the following amino acid residues: His-6: H, I, L, M and V; Ala-33: A, D, E and Q; and Phe-50: F, V, L and A. Complete degeneracy was used at position Met-45 by using an NNK codon. The number of possible variants in this library equaled 1,600. To construct this library, an equimolar mixture of the genes coding for A33D-, A33E-, A33Q- and wild-type 4-OT in the pJexpress 414 plasmid was used as a template in the PCR. The forward primers were an equimolar mixture of oligonucleotides carrying a mutagenic codon at position 6 (entry 7–11; Supplementary Table 8) and the reverse primers were an equimolar mixture of oligonucleotides carrying a mutagenic codon at position 50 and an NNK codon at position 45 (entries 3–6; Supplementary Table 8). In total, 35 PCR cycles were carried out with the following temperatures and times: denaturation at 95 °C (30 s), annealing at 60 °C (60 s) and elongation at 72 °C (60 s). The PCR product was gel purified, digested with NdeI and BamHI, and cloned into an empty pJexpress 414 plasmid, which was compatibly digested and treated with alkaline phosphatase. After the ligation, the plasmid was transformed into chemically competent *E. coli* DH5α. The transformants were selected at 37 °C on LB agar plates containing 100 μg ml^−1^ amp. Plasmid DNA was extracted from a few randomly selected transformants and the entire 4-OT gene was sequenced, to ensure that the desired mutations were introduced. After establishing that the library was of good quality, the library was collected by pooling ∼5,200 DH5α colonies, an aliquot of which was used for plasmid DNA isolation. An aliquot of this isolated plasmid mixture was used to transform chemically competent *E. coli* BL21 (DE3) cells. Transformants were selected at 37 °C on LB agar plates containing 100 μg ml^−1^ amp. Of these transformants, 1,600 were randomly chosen and grown in 1.25 ml liquid LB/amp medium. These cultures were used to produce CFE according to the above-mentioned protocol. The 4-OT mutants in the CFE were screened for their activities for the Michael-type addition of **3** to **5a** and **4** to **5a**. The same assay conditions as described above were used, but without assessing the 4-OT expression levels. After screening the 1,600 members, 55 were selected for their pronounced activity. These mutants were again assayed for their Michael-type addition activities (**3** to **5a** and **4** to **5a)** and tested in a control assay monitoring the depletion of **5a** in the absence of aldehyde. In addition, the expression level of each of the mutant enzymes was assessed using the densitometric assay as described above. The most active mutant enzymes were identified based on their specific activities relative to wild-type 4-OT ((U_mut_ × mg^−1^)/(U_wt_ × mg^−1^)) for the Michael-type additions. The plasmid DNA of the corresponding transformants was extracted and the entire 4-OT gene was sequenced to identify the mutations.

4-OT purification method. All 4-OT enzymes were purified using a procedure described elsewhere^28^. This procedure required adjustments for the purification of the 4-OT mutants H6M/A33E/F50V and H6L/A33E/F50V. In these cases, the elution of enzyme from the DEAE-sepharose column was done using 0.1 M Na_2_SO_4_ (instead of 0.5 M). The protein precipitation was conducted with 1.6 M (NH_4_)_2_SO_4_ in accordance with the protocol. However, after the precipitation step the concentration of (NH_4_)_2_SO_4_ was reduced to 1.0 M by adding the appropriate amount of 10 mM NaH_2_PO_4_ buffer (pH 7.3). The solution was loaded on a phenyl-sepharose column (equilibrated with 1.0 M (NH_4_)_2_SO_4_) and washed with 1.0 M (NH_4_)_2_SO_4_ (instead of 1.6 M). These adjustments were required because of the higher binding affinity of H6M/A33E/F50V and H6L/A33E/F50V for the phenyl-sepharose column material compared with wild-type 4-OT. Purified H6M/A33E/F50V and H6L/A33E/F50V enzymes were obtained in the flow-through and wash fractions during this column chromatography step. The subsequent desalting, concentrating and concentration assessment of the purified enzyme was done according to a literature procedure^28^. All purified 4-OT variants were analysed by electrospray ionization mass spectrometry to confirm the correct mass of the proteins.

Characterization of H6L/A33E/F50V and H6M/A33E/F50V. To confirm the improved ‘Michaelase' activity of the engineered 4-OT variants H6L/A33E/F50V and H6M/A33E/F50V, a progress curve analysis was done using these biocatalysts in the Michael-type addition of **4** to **5a** (Supplementary Fig. 28). The reaction mixture for this analysis consisted of the following: **4** (100 mM), **5a** (2 mM), 4-OT (0.7 mol% relative to **5a**, based on monomer concentration) and ethanol (5% v/v) in 20 mM NaH_2_PO_4_ (pH 5.5); the final volume of this reaction mixture was 20 ml. Samples were taken from the reaction mixture and analysed by ultraviolet spectroscopy (320 nm) at timely intervals, to monitor the depletion of **5a**. After the reaction was completed, product **7** was extracted with ethyl acetate (3 × 25 ml). The combined organic layers were dried using anhydrous MgSO_4_ and concentrated *in vacuo* to yield **7**. The product was analysed by chiral HPLC to establish the e.r. values (Supplementary Table 3).

Construction and testing of double and triple 4-OT mutants for improved enantioselectivity towards 2S3R-7. Double and triple mutants at positions His-6, Met-45 and Phe-50 were constructed by PCR using the appropriate combinations of template and primers. The gene coding for either M45Y, M45H or wild-type 4-OT in the pJexpress 414 vector was used as a template in the PCR in combination with either reverse primer ‘Rev. WT 4-OT' or ‘Rev. F50A' and forward primer ‘Fwd. WT4-OT' or ‘Fwd. H6I' (Supplementary Table 8, entries: 1, 2, 7 and 8). The PCR product was gel purified, digested with NdeI and BamHI, and cloned into an empty pJexpress 414 plasmid, which was compatibly digested and treated with alkaline phosphatase. After the ligation, the plasmid was transformed into chemically competent *E. coli* DH5α cells. The transformants were selected at 37 °C on LB agar plates containing 100 μg ml^−1^ amp. Plasmid DNA was extracted from randomly picked transformants and the entire 4-OT gene was sequenced, to ensure that the desired mutations were introduced. An aliquot of this plasmid solution was used to transform chemically competent *E. coli* BL21 (DE3) cells. CFEs prepared from cultures of these cells, expressing the 4-OT mutant proteins, were used to determine the ability of these 4-OT mutants to produce enantioenriched **7** in the Michael-type addition of **4** to **5a** according to the earlier described protocol (see above). The e.r. values of **7** produced by these mutants are listed in Supplementary Table 6, depicting only those mutants that were active.

### Semi-preparative scale synthesis experiments

Semi-preparative scale synthesis of **6a**. To demonstrate the synthetic usefulness of the 4-OT mutants A33D and M45Y/F50A for the production of enantioenriched **6a**, relative to wild-type 4-OT, semi-preparative scale synthesis experiments were conducted using these three enzymes. For this, an amount of enzyme (6 or 12 mg) was added to a 100-ml round-bottom flask, after which 20 mM NaH_2_PO_4_ buffer (pH 7.3, 6.5 or 5.5) was added, adjusting the final volume to 54 ml. The reaction was initiated by the addition of **3** (170 μl) and 6 ml of a stock solution of **5a** (20 mM in ethanol). The final concentrations were as follows: **5a** (2 mM), **3** (50 mM), 4-OT (14.7 or 29.4 μM, based on monomer concentration) and 10% ethanol (v/v) in 20 mM NaH_2_PO_4_ buffer. Samples were taken from the round-bottom flask at timely intervals for ultraviolet spectrophotometric analysis, to monitor the reaction progress by following the depletion of **5a** (Fig. 4a and Supplementary Fig. 4). After each measurement, the sample was recombined with the original reaction mixture in the round-bottom flask. When the depletion of **5a** was complete, the product (**6a**) was extracted with ethyl acetate (3 × 40 ml) using a separatory funnel. The organic phases were combined, dried with MgSO_4_ and evaporated *in vacuo*, yielding a yellowish oil. The crude oil obtained after a reaction at pH 7.3 was purified using flash column chromatography (silica gel, ethyl acetate: n-Heptane 1:4 (v/v), 3 ml min^−1^ flow); the fractions containing **6a** were identified using thin-layer chromatography (ethyl acetate: n-Heptane 1:2 (v/v), visualization: KMnO_4_) and combined. The product was concentrated *in vacuo*, redissolved in CDCl_3_ and a ^1^H NMR spectrum was recorded (Supplementary Fig. 2) on a Bruker DRX-500 (500 MHz) spectrometer; all spectra were referenced to residual CHCl_3_ (*δ*=7.26). The crude oil obtained after the reactions at pH 5.5 and pH 6.5 did not require flash chromatography, as the ^1^H NMR spectra (Supplementary Figs 2b and 5) indicated that **6a** was obtained in high purity. After confirming by ^1^H NMR analysis that product **6a** was obtained, the yields were determined (wild-type 4-OT: 8.2 mg, 4.2 × 10^−2^ mmol, 36%; M45Y/F50A: 6.9 mg, 3.6 × 10^−2^ mmol, 30%; M45Y/F50A (pH 5.5): 15.2 mg, 7.9 × 10^−2^ mmol, 65%; A33D: 6.5 mg, 3.4 × 10^−2^ mmol, 28%; wild-type 4-OT (pH 6.5): 22 mg, 1.1 × 10^−1^ mmol, 94%; and A33D (pH 6.5): 22 mg, 1.1 × 10^−1^ mmol, 94%). Aliquots of **6a** were converted to their corresponding ethylene glycol acetals according to a literature procedure^29^. The e.r. of the derivatized **6a** was determined using reverse-phase HPLC with a Chiralpak AD-RH column (150 mm × 4.6 mm, Daicel) (see Supplementary Figs 3 and 6 for the chromatograms).

Semi-preparative scale synthesis of **7**. To demonstrate the synthetic usefulness of the 4-OT mutants H6M/A33E/F50V, R39E and M45Y/F50A for the production of enantioenriched **7**, relative to wild-type 4-OT, semi-preparative scale synthesis experiments were conducted using these four enzymes. For this, 6 mg of enzyme was added to a 100-ml round-bottom flask, after which 20 mM NaH_2_PO_4_ buffer (pH 5.5) was added, adjusting the final volume to 54 ml. The reaction was initiated by the addition of **4** (540 μl) and 6 ml of a stock solution of **5a** (20 mM in ethanol). The final concentrations were as follows: **5a** (2 mM), **4** (100 mM), 4-OT (14.7 μM, based on monomer concentration) and 10% ethanol (v/v) in 20 mM NaH_2_PO_4_ buffer (pH 5.5). Samples were taken from the round-bottom flask at timely intervals for ultraviolet spectrophotometric analysis, to monitor the reaction progress by following the depletion of **5a** (Fig. 4b). After each measurement, the sample was recombined with the original reaction mixture in the round-bottom flask. When the depletion of **5a** was complete, the product (**7**) was extracted with ethyl acetate (3 × 40 ml) using a separatory funnel. The organic phases were combined, dried with MgSO_4_ and evaporated *in vacuo*, yielding a yellowish oil. The crude oil was dissolved in CDCl_3_ and a ^1^H NMR spectrum was recorded (Supplementary Fig. 7) using a Bruker DRX-500 (500 MHz) spectrometer (all spectra were referenced to residual CHCl_3_ (*δ*=7.26)). After confirming that product **7** was obtained, the yields were determined and corrected for the presence of small amounts of 2-nitro-1-phenylethanol (**10**), which is the product of the non-enzymatic water addition to **5a** (see Supplementary Fig. 7). The corrected yields were as follows: wild-type: 17.7 mg, 8.0 × 10^−2^ mmol, 67%; H6M/A33E/F50V: 20.2 mg, 9.1 × 10^−2^ mmol, 76%; R39E: 17.6 mg, 8.0 × 10^−2^ mmol, 66%; and M45Y/F50A: 22.7 mg, 10.2 × 10^−2^ mmol, 86%. The e.r. was determined using reverse-phase HPLC with the Chiralpak ID column (150 mm × 4.6 mm, Daicel) (see Supplementary Fig. 8 for the chromatograms).

Optical rotation analysis of **6a** and **7**. The optical rotations of enzymatically produced **6a** and **7** were measured in CHCl_3_ on a Schmidt+Haensch polarimeter (polartronic MH8) with a 10-cm cell (*c* is given in grams per 100 ml). The data are presented in Supplementary Table 1.

Semi-preparative scale synthesis of **6b**–**g** using A33D and M45Y/F50A. To investigate the synthetic usefulness of 4-OT A33D and 4-OT M45Y/F50A for the enantioselective synthesis of γ-nitroaldehydes **6b–g**, semi-preparative scale reactions were conducted using these engineered 4-OT variants. The reaction conditions of these semi-preparative scale reactions and the e.r. determinations were based on procedures described in earlier reports^29^,^31^. The general procedure of these experiments was as follows: the appropriate amount of biocatalyst, substrate **3** and co-solvent were diluted in buffer (20 mM NaH_2_PO_4_, pH 5.5). The reactions were initiated by the addition of the nitroalkene (**5b**–**g**) and the reaction progress was monitored using ultraviolet spectrophotometric analysis. After the ultraviolet signal of the nitroalkene vanished, the products (**6b**–**g**) were extracted from the reaction mixtures and characterized by ^1^H NMR spectroscopy and chiral HPLC or chiral GC, and the yields were determined. One adjustment on the earlier reported reaction conditions was made in the buffer pH of the A33D-catalysed reactions yielding compounds **6d** and **6e**. In these reactions, ethanol was used as a co-solvent instead of dimethyl sulfoxide (DMSO) and the A33D enzyme appeared to precipitate under these conditions. To reduce protein precipitation, the buffer pH was increased to pH 6.5. More details on the reaction conditions (Supplementary Table 9), work-up procedures and e.r. determinations are listed below.

### 3-(4-Chlorophenyl)-4-nitrobutanal (**6b**)

The 4-OT-catalysed Michael-type addition of acetaldehyde (**3**) to *trans*-4-chloro-β-nitrostyrene (**5b**) using 4-OT A33D or 4-OT M45Y/F50A was conducted under earlier reported optimized reaction conditions^31^. The reaction mixtures consisted of the following: 4-OT (2.8 mol%, relative to **5b**), **3** (65 mM), **5b** (1.3 mM) and DMSO (45% v/v) in 20 mM NaH_2_PO_4_ buffer (pH 5.5); the final reaction volume was 50 ml (see Supplementary Table 9). After ultraviolet spectrophotometric analysis of the reaction mixtures revealed full conversion of the nitroalkene (Supplementary Fig. 9), the reaction mixtures were diluted with H_2_O till a final DMSO concentration of 10% (v/v) was reached. Next, compound **6b** was extracted with ethyl acetate (3 × 60 ml). The combined organic layers were washed with H_2_O (3 × 60 ml), to eliminate traces of DMSO, and dried with brine (25 ml) and anhydrous Na_2_SO_4_. The dried organic layer was concentrated *in vacuo* to yield 3-(4-chlorophenyl)-4-nitrobutanal (**6b**) (A33D: 12 mg, 5.3 × 10^−2^ mmol, 81% and M45Y/F50A: 9 mg, 3.93 × 10^−2^ mmol, 60%) as a colourless oil. The ^1^H NMR spectroscopic data of **6b** (Supplementary Fig. 15) are in agreement with previously published data^1^,^31^. Enantiomeric ratios were determined by reverse-phase HPLC using a Chiralpak AD-RH column (150 mm × 4.6 mm, Daicel) (MeCN/water 32:68, 25 °C) at 0.5 ml min^−1^. Ultraviolet detection at 220 nm: *t*_R_: *3R-***6b**=50.8 min, *3S-***6b**=54.6 min led to the following e.r. (*3S:3R)*: racemic **6b**=50:50, 4-OT A33D **6b**=98:2 and 4-OT M45Y/F50A **6b**=38:62 (Supplementary Fig. 21). The assignment of the absolute configuration was based on earlier reported chiral HPLC data^31^.

### 3-(4-Fluorophenyl)-4-nitrobutanal (**6c**)

The 4-OT-catalysed Michael-type addition of **3** to *trans*-4-fluoro-β-nitrostyrene (**5c)** using 4-OT A33D or 4-OT M45Y/F50A was conducted under earlier reported optimized reaction conditions^31^. The reaction mixtures consisted of the following: 4-OT (1.5 mol%, relative to **5c**), **3** (50 mM), **5c** (2.0 mM) and DMSO (40% v/v) in 20 mM NaH_2_PO_4_ buffer (pH 5.5); the final reaction volume was 60 ml (see Supplementary Table 9). After ultraviolet spectrophotometric analysis of the reaction mixtures revealed full conversion of the nitroalkene (Supplementary Fig. 10), the reaction mixtures were diluted with H_2_O till a final DMSO concentration of 10% (v/v) was reached. Next, compound **6c** was extracted with ethyl acetate (3 × 60 ml). The combined organic layers were washed with H_2_O (3 × 60 ml), to eliminate traces of DMSO, and dried with brine (25 ml) and anhydrous Na_2_SO_4_. The dried organic layer was concentrated *in vacuo* to yield 3-(4-fluorophenyl)-4-nitrobutanal (**6c**) (A33D: 16 mg, 7.6 × 10^−2^ mmol, 63% and M45Y/F50A: 12 mg, 5.8 × 10^−2^ mmol, 47%) as a colourless oil. The ^1^H NMR spectroscopic data of **6c** (Supplementary Fig. 16) are in agreement with previously published data^31^,^50^. Enantiomeric ratios were determined by reverse-phase HPLC using a Chiralpak AD-RH column (150 mm × 4.6 mm, Daicel) (MeCN/water 30:70, 25 °C) at 0.5 ml min^−1^. Ultraviolet detection at 220 nm: *t*_R_: *3R-***6c**=37.4 min, *3S-***6c**=39.0 min led to the following e.r. (*3S:3R)*: racemic **6c**=49:51, 4-OT A33D **6c**=99:1 and 4-OT M45Y/F50A **6c**=14:86 (Supplementary Fig. 22). The assignment of the absolute configuration was based on earlier reported chiral HPLC data^31^.

### 3-(4-Hydroxyphenyl)-4-nitrobutanal (**6d**)

The 4-OT-catalysed Michael-type addition of **3** to *trans*-4-hydroxy-β-nitrostyrene (**5d**) using 4-OT A33D and 4-OT M45Y/F50A was conducted based on earlier reported reaction conditions^29^. The reaction mixtures consisted of the following: 4-OT (0.5 mol%, relative to **5d**), **3** (50 mM), **5d** (2.0 mM) and ethanol (10% v/v) in 20 mM NaH_2_PO_4_ buffer (pH 6.5) for the A33D-catalysed reaction and in 20 mM NaH_2_PO_4_ buffer (pH 5.5) for the M45Y/F50A-catalysed reaction. The final reaction volume was 60 ml (see Supplementary Table 9). After ultraviolet spectrophotometric analysis of the reaction mixtures revealed almost complete nitroalkene conversion (Supplementary Fig. 11), compound **6d** was extracted with ethyl acetate (3 × 40 ml). The combined organic layers were dried with brine (25 ml) and anhydrous Na_2_SO_4_, and concentrated *in vacuo* to yield 3-(4-hydroxyphenyl)-4-nitrobutanal (**6d**) (A33D: 11 mg, 5.2 × 10^−2^ mmol, 44% and M45Y/F50A: 13 mg) as a yellowish oil. ^1^H NMR spectroscopic analysis of the obtained product from the 4-OT M45Y/F50A-catalysed reaction revealed incomplete conversion (∼92%) of **5d** (Supplementary Fig. 17). The yield of the product obtained after the M45Y/F50A-catalysed reaction was therefore corrected for this contamination of 6% (w/w) **5d**. The corrected yield of product **6d** from the M45Y/F50A-catalysed reaction was 12 mg (5.8 × 10^−2^ mmol, 48%). The ^1^H NMR spectroscopic data of **6d** (Supplementary Fig. 17) are in agreement with previously published data^29^. The aldehyde functionality of **6d** was derivatized into a cyclic acetal to determine the e.r. by reverse-phase HPLC using a Chiralpak AD-RH column (150 mm × 4.6 mm, Daicel) (MeCN/water 67:33, 25 °C) at 0.5 ml min^−1^. Ultraviolet detection at 210 nm: *t*_R_: deriv.**-***3R-***6d**=5.8 min, deriv.**-***3S-***6d**=8.0 min led to the following e.r. (*3S:3R)*: racemic **6d**=51:49, 4-OT A33D **6d**=95:5 and 4-OT M45Y/F50A **6d**=18:82 (Supplementary Fig. 23). As a different stationary phase was used compared with literature, the optical rotations of the enzymatically obtained product **6d** were recorded for assigning the absolute configuration of the product. The optical rotations were A33D **6d**: 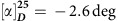 (*c*=0.8, CHCl_3_) and YA **6d**: 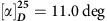 (*c*=1.3, CHCl_3_). The absolute configuration of the major enantiomers was tentatively assigned based on the sign of the optical rotation compared with other γ-nitroaldehydes and based on the assumption that A33D and M45Y/F50A generate Michael-type addition products with a consistent geometry at the chiral centre (see Tables 2 and 3)^31^.

### 3-(3-Hydroxy-4-methoxyphenyl)-4-nitrobutanal (**6e**)

The 4-OT-catalysed Michael-type addition of **3** to *trans*-4-methoxy-3-hydroxy-β-nitrostyrene (**5e**) using 4-OT A33D or 4-OT M45Y/F50A was conducted under earlier reported optimized reaction conditions^31^. The reaction mixtures consisted of the following: 4-OT (1.8 mol%, relative to **5e**), **3** (50 mM), **5e** (2.0 mM) and ethanol (10% v/v) in 20 mM NaH_2_PO_4_ buffer (pH 6.5) for the A33D-catalysed reaction and in 20 mM NaH_2_PO_4_ buffer (pH 5.5) for the M45Y/F50A-catalysed reaction. The final reaction volume was 60 ml (see Supplementary Table 9). After ultraviolet spectrophotometric analysis of the reaction mixtures revealed full conversion of the nitroalkene (Supplementary Fig. 12), compound **6e** was extracted from the reaction mixture with ethyl acetate (3 × 25 ml). The combined organic layers were dried with brine (2 × 25 ml) and anhydrous Na_2_SO_4_, and concentrated *in vacuo* to yield 3-(3-hydroxy-4-methoxyphenyl)-4-nitrobutanal (**6e**) (A33D: 27 mg, 11.2 × 10^−2^ mmol, 94% and M45Y/F50A: 21 mg, 8.8 × 10^−2^ mmol, 73%) as a colourless oil. The ^1^H NMR spectroscopic data of **6e** (Supplementary Fig. 18) are in agreement with previously published data^31^. The aldehyde functionality of **6e** was derivatized into a cyclic acetal to determine the e.r. by reverse-phase HPLC using a Chiralpak AD-RH column (150 mm × 4.6 mm, Daicel) (MeCN/water 33:67, 25 °C) at 0.8 ml min^−1^. Ultraviolet detection at 220 nm: *t*_R_: deriv.**-***3R-***6e**=20.6 min, deriv.**-***3S-***6e**=31.2 min led to the following e.r. (*3S:3R)*: racemic **6e**=49:51, 4-OT A33D **6e**=99:1 and 4-OT M45Y/F50A **6e**=3:97 (Supplementary Fig. 24). The assignment of the absolute configuration was based on earlier reported chiral HPLC data^31^.

### 3-(3-(Cyclopentyloxy)-4-methoxyphenyl)-4-nitrobutanal (**6f**)

The 4-OT-catalysed Michael-type addition of **3** to *trans*-4-methoxy-3-cyclopentyloxy-β-nitrostyrene (**5f**) using 4-OT A33D and 4-OT M45Y/F50A was conducted under earlier reported optimized reaction conditions^31^. The reaction mixtures consisted of the following: 4-OT (3.7 mol%, relative to **5f**), **3** (50 mM), **5f** (2.0 mM) and DMSO (40% v/v) in 20 mM NaH_2_PO_4_ buffer (pH 5.5); the final reaction volume was 60 ml (see Supplementary Table 9). After ultraviolet spectrophotometric analysis of the reaction mixtures revealed full conversion of the nitroalkene (Supplementary Fig. 13), the reaction mixtures were diluted with H_2_O till a final DMSO concentration of 10% (v/v) was reached. Next, compound **6f** was extracted with ethyl acetate (3 × 60 ml). The combined organic layers were washed with H_2_O (3 × 60 ml), to eliminate traces of DMSO, and dried with brine (25 ml) and anhydrous Na_2_SO_4_. The dried organic layer was concentrated *in vacuo*, to yield 3-(3-(cyclopentyloxy)-4-methoxyphenyl)-4-nitrobutanal (**6f**) (A33D: 24 mg, 7.8 × 10^−2^ mmol, 65% and M45Y/F50A: 26 mg, 8.5 × 10^−2^ mmol, 70%) as a colourless oil. The ^1^H NMR spectroscopic data of **6f** (Supplementary Fig. 19) are in agreement with previously published data^4^,^31^. The aldehyde functionality of **6f** was derivatized into a methyl ester (see ‘Derivatization of **6a**, **6d**, **6e** and **6f** for enantiomeric excess determination'), to determine the e.r. by normal-phase HPLC using a Chiralpak IB column (150 mm × 4.6 mm, Daicel) (*n*-heptane/*i*-PrOH 95:5, 25 °C) at 1 ml min^−1^. Ultraviolet detection at 220 nm: *t*_R_: deriv.**-***3R-***6f**=12.0 min, deriv.**-***3S-***6f**=13.5 min led to the following e.r. (*3S:3R*): racemic **6f**=51:49, 4-OT A33D **6f**>99:1 and 4-OT M45Y/F50A **6f**=32:68 (Supplementary Fig. 25). The assignment of the absolute configuration was based on earlier reported chiral HPLC data^31^.

### 5-Methyl-3-(nitromethyl)hexanal (**6g**)

The 4-OT-catalysed Michael-type addition of **3** to (*E*)-4-methyl-1-nitropent-1-ene (**5g**) using 4-OT A33D or 4-OT M45Y/F50A was conducted under optimized reaction conditions as earlier reported^31^. The reaction mixtures consisted of the following: 4-OT (5.3 mol%, relative to **5g**), **3** (150 mM), **5g** (3.0 mM) and DMSO (40% v/v) in 20 mM NaH_2_PO_4_ (pH 5.5); the final reaction volume was 12.8 ml (see Supplementary Table 9). After ultraviolet spectrophotometric analysis of the reaction mixtures revealed full conversion of the nitroalkene (Supplementary Fig. 14) (A33D: 50 min and M45Y/F50A: 70 min), compound **6g** was extracted from the reaction mixture with toluene (3 × 10 ml) and the combined organic layers were washed with dH_2_O (3 × 5 ml) to eliminate traces of DMSO. The organic layer was dried with brine (5 ml) and anhydrous Na_2_SO_4_, and concentrated *in vacuo* to yield 5-methyl-3-(nitromethyl)hexanal (**6g**) (A33D: 4 mg, 2.3 × 10^−2^ mmol, 60% and M45Y/F50A: 4 mg, 2.3 × 10^−2^ mmol, 60%) as a colourless oil. The ^1^H NMR spectroscopic data of **6g** (Supplementary Fig. 20) are in agreement with previously published data^1^,^31^. Enantiomeric ratios were determined by GC using an Hewlett Packard (HP) chiral 20% permethylated β-cyclodextrin column (20 m, 90 °C isocratic, 1.5 ml min^−1^). Flame ionization detection: *t*_R_: *3S-***6g**=65.6 min and *3R-***6g**=67.5 min led to the following e.r. (*3R:3S*): racemic **6g**=50:50, 4-OT A33D **6g**≥99:1 and 4-OT M45Y/F50A **6g**=10:90 (Supplementary Fig. 26). The assignment of the absolute configuration was based on earlier reported chiral GC data^31^.

### Derivatization of **6a** and **6d**–**f** for enantiomeric excess determination

Acetalysation of **6a**, **6d** and **6e**. The aldehyde functionality of **6a**, **6d** and **6e** was derivatized into a cyclic acetal, according to a literature procedure^29^,^31^. The γ-nitroaldehyde (5 mg), ethylene glycol (25.6 mg, 0.4 mmol) and p-TsOH (5 mol%, 0.2 mg, 1.0 × 10^−3^ mmol) were stirred in chloroform (800 μl) under a nitrogen atmosphere for 1 day at room temperature, to yield the cyclic acetal of the γ-nitroaldehyde.

Esterification of **6f**. The aldehyde functionality of **6f** was converted into a methyl ester according to a literature procedure^31^. A solution of KH_2_PO_4_ (1 mg, 7.35 μmol), NaClO_2_ (0.65 mg, 7.2 μmol) and MeOH (75 μl, 16.3 μmol) in dH_2_O (75 μl) were cooled down to 0 °C, and **6f** (5 mg, 2.3 × 10^−2^ mmol) in acetonitrile (75 μl) was added. After the addition of H_2_O_2_ (35% solution, 8.5 μl), the mixture was stirred at room temperature for 2 h. The pH was adjusted to 3 with 0.1 M HCl and saturated Na_2_SO_3_ solution (200 μl) was added. The product was extracted with CH_2_Cl_2_ (3 × 1 ml). The combined organic layers were washed with dH_2_O (500 μl), dried on Na_2_SO_4_ and concentrated *in vacuo*. The residue was dissolved in toluene (85 μl) and methanol (165 μl), and cooled down to 0 °C. To this cooled solution, trimethylsilyl diazomethane (20 μl, 40 μmol, 2.0 M in n-hexane) was added and stirred at 0 °C for 5 min. The solution was stirred for additional 15 min at room temperature and the reaction quenched with concentrated AcOH. The solvents were evaporated under vacuum, to yield methyl 3-(3-(cyclopentyloxy)-4-methoxyphenyl)-4-nitrobutanoate.

### Synthesis of nitroalkenes **5a**–**g**

Nitroalkenes **5a**–**d** were commercially available, whereas **5e**–**g** were synthesized and purified according to literature procedures^31^,^51^,^52^,^53^,^54^.

### Synthesis of racemic **6a**–**g**

The racemic products **6a**–**g**, which served as references in the chiral-HPLC and chiral-GC analyses, were synthesized and purified according to literature procedures^31^.

### Structural analysis of M45Y/F50A

Crystallization of M45Y/F50A. Before crystallization, purified enzyme was subjected to size-exclusion chromatography using 20 mM Tris-HCl (pH 7.5), containing 200 mM NaCl, as an eluent. Fractions containing 4-OT M45Y/F50A were pooled and concentrated to 10 mg ml^−1^. Dynamic light scattering (DynaPro NanoStar, Wyatt Technology, CA) confirmed that the mutant existed as a hexamer in solution. Mass spectrometric analysis indicated that the enzyme had been correctly processed with the N-terminal methionine being cleaved off, leaving an exposed Pro-1. Attempts to reproduce the previously reported condition of crystallization for wild-type 4-OT were unsuccessful^32^. Therefore, a screening for new conditions was carried out at room temperature in 96-well sitting-drop crystallization plates using various available commercial screens. Drops (300 nl) were prepared with a nanodispenser (Mosquito, TTP Labtech) by mixing protein and reservoir solutions at a 1:1 ratio. The structure of M45Y/F50A was solved from a crystal that appeared in the following crystallization condition: 0.2 M sodium formate, 0.1 M bis-tris propane pH 8.5 and 20% PEG 3350 (w/v). Small crystals appeared after 3 days and grew to a size of ∼100 × 75 × 75 μm^3^ within 7 days. In addition, M45Y/F50A was co-crystallized in the presence of acetaldehyde (**3**) and *trans-*β-nitrostyrene (**5a**) with the aim of trapping a substrate or product-bound structure. A stock solution of 100 mM **5a** was prepared in DMSO. Before setting up drops, M45Y/F50A was incubated with 50 mM **3** and 10 mM **5a** for 30 min. A screening was conducted to find new conditions as described above. The final concentration of DMSO in the drops was 5% (v/v). A single crystal with dimensions of 150 × 100 × 100 μm^3^ appeared in a condition consisting of 0.1 M PCTP buffer pH 7.0 and 25% (w/v) PEG 1500 after 2 weeks.

Data collection and structure determination. Before proceeding with data collection, crystals were transferred to a drop containing the mother liquor together with 15% glycerol (v/v). Crystals were directly frozen in the liquid N_2_ stream of the X-ray data collection equipment. Data sets for both crystals were collected in-house at 110 K, using a Microstar rotating anode (Cu, wavelength of 1.5418 Å) X-ray source (Bruker AXS GmbH), coupled with Helios optics (Incoatec GmbH) and a MAR345dtb detector (Marresearch GmbH). Diffraction data sets were processed, scaled and merged using XDS^55^ and Aimless^56^. The M45Y/F50A structure belonged to the space group C2, whereas the M45Y/F50A structure in complex with substrate **5a** crystallized in the P1 space group. A summary of the data collection and model refinement statistics is given in Supplementary Table 7.

Phaser^57^ from the CCP4 software suite^58^ was used to calculate initial phases. The structure of wild-type 4-OT (PDB ID: 4X19)^32^ was used as a search model, to perform molecular replacement for the M45Y/F50A structure. Subsequently, multiple cycles of refinement were performed using Refmac5 (ref. 59), together with manual model building in COOT^60^ to improve the structure. The final cycles of refinement were carried out in phenix.refine available from the Phenix software suite^61^. The coordinates and topology for **5a** were generated using the PRODRG server^62^. Target values for the bond lengths and bond angles, used in refinement, were derived from the crystal structure of **5a**^63^, deposited in Cambridge Structural Database (CSD entry: KANWOL10).

Crystal structure analysis. Calculation of Cα-backbone root-mean-square deviation values and superpositions of structures were performed using the protein structure comparison service at the European Bioinformatics Institute (PDBeFold)^64^. PyMol (the PyMOL molecular graphics system, version 1.6; Schrodinger, LLC.) was used to prepare figures and carry out analysis of the structures. Molprobity, available as a part of the phenix.refine module, was used to validate the stereochemical quality of the models^65^. Coordinates for the free and nitrostyrene-bound structure of M45Y/F50A have been deposited with the Protein Data Bank (accession codes 5CLN and 5CLO, respectively).

## Additional information

**Accession codes:** Coordinates for the free and nitrostyrene-bound structure of M45Y/F50A have been deposited with the Protein Data Bank (PDB accession codes 5CLN and 5CLO, respectively).

**How to cite this article:** van der Meer, J.-Y. *et al.* Using mutability landscapes of a promiscuous tautomerase to guide the engineering of enantioselective Michaelases. *Nat. Commun.* 7:10911 doi: 10.1038/ncomms10911 (2016).

## Supplementary Material

Supplementary Information Supplementary Figures 1-31, Supplementary Tables 1-9, Supplementary Discussion and Supplementary References

## Figures and Tables

**Table 1:** Preparative scale Michael-type addition reactions of aldehydes (**3** or **4**) to *trans*-nitrostyrene (**5a**) catalysed by wild-type 4-OT or 4-OT mutants (0.7 mol%).

**Entry**	**Substrates**	**Catalyst**	**Solvent pH**	**Reaction time (h)**	**Yield (%)**	d.r.* (***syn***:***anti***)	**e.r.**†	**Abs.**‡ **conf.**	**Product**
1	**3** and **5a**	WT 4-OT	7.3	6	36§	—	93: 7	*3S*	**6a**
2	**3** and **5a**	A33D	7.3	1.5	28§	—	99: 1	*3S*	**6a**
3	**3** and **5a**	M45Y/F50A	7.3	3	30§	—	94: 6	*3R*	**6a**
4	**3** and **5a**	WT 4-OT	6.5	1.8||	94	—	95: 5	*3S*	**6a**
5	**3** and **5a**	A33D	6.5	0.7||	94	—	99: 1	*3S*	**6a**
6	**3** and **5a**	M45Y/F50A	5.5	21	65	—	96: 4	*3R*	**6a**
7	**4** and **5a**	WT 4-OT	5.5	23	67	91: 9	57: 43	*2R3S*	**7**
8	**4** and **5a**	H6M/A33E/F50V	5.5	1	76	97: 3	77: 23	*2S3R*	**7**
9	**4** and **5a**	R39E	5.5	23	67	93: 7	95: 5	*2R3S*	**7**
10	**4** and **5a**	M45Y/F50A	5.5	2.5	86	96: 4	96: 4	*2S3R*	**7**

HPLC, high-performance liquid chromatography; 4-OT, 4-oxalocrotonate tautomerase.

^*^The d.r. values were determined by ^1^H NMR spectroscopy (Supplementary Fig. 7).

^†^The e.r. values were determined by HPLC on a chiral stationary phase (Supplementary Figs 3, 6 and 8).

^‡^The absolute configuration of the major enantiomers was determined by comparison of chiral-phase HPLC data and optical rotation data with literature data (Supplementary Table 1).

^§^At pH 7.3, the formation of undefinable side products, resulting from the inherent tendency of acetaldehyde to form oligomers^44^, requires product purification by flash column chromatography, which lowers the isolated yield of the desired product **6a**.

^||^Catalyst loading (1.4 mol%) was used.

The progress curves for these preparative scale reactions are given in Fig. 4 and Supplementary Fig. 4.

**Table 2:**
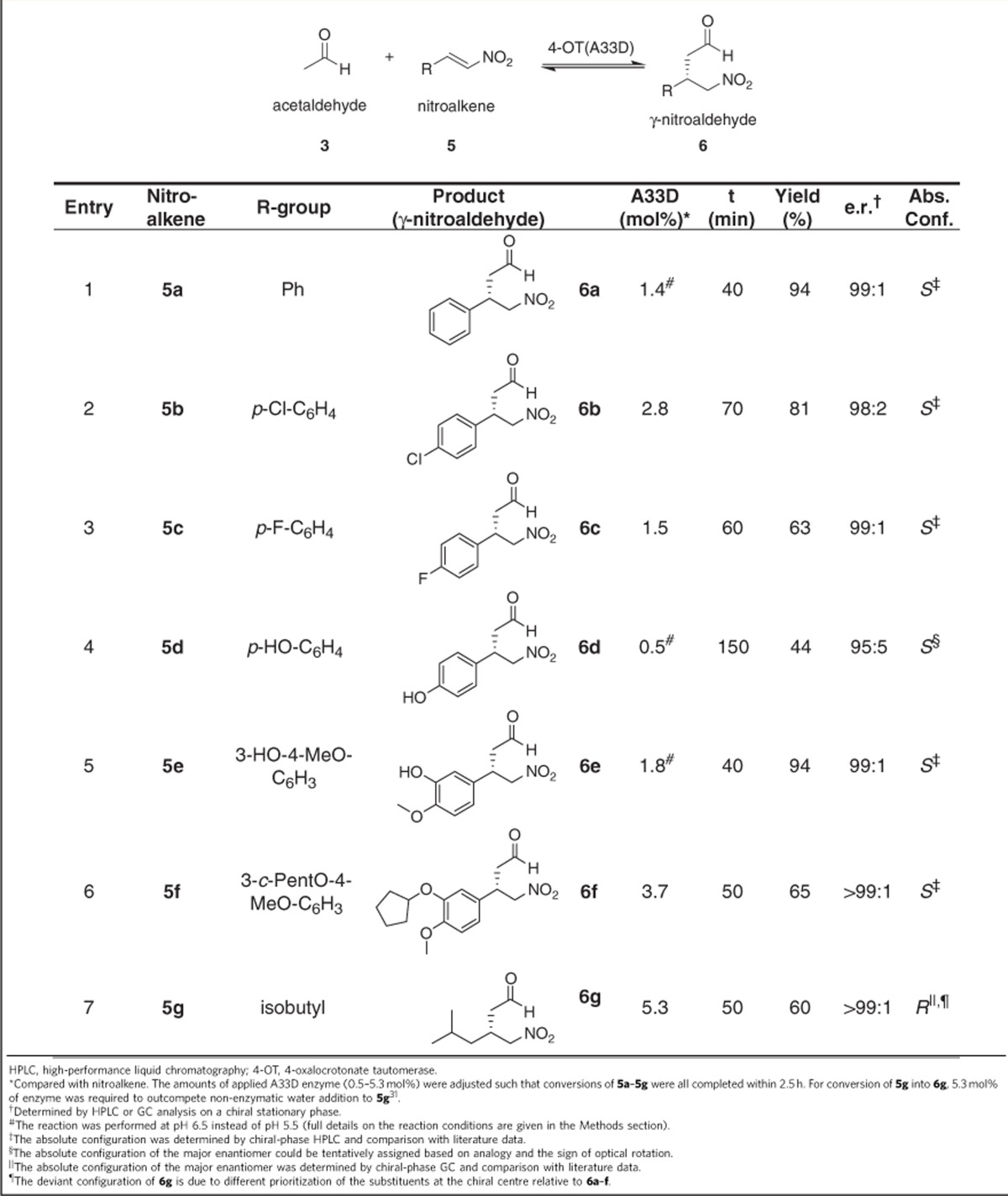
4-OT(A33D)-catalysed acetaldehyde addition to nitroalkenes 5a–5g.

**Table 3:**
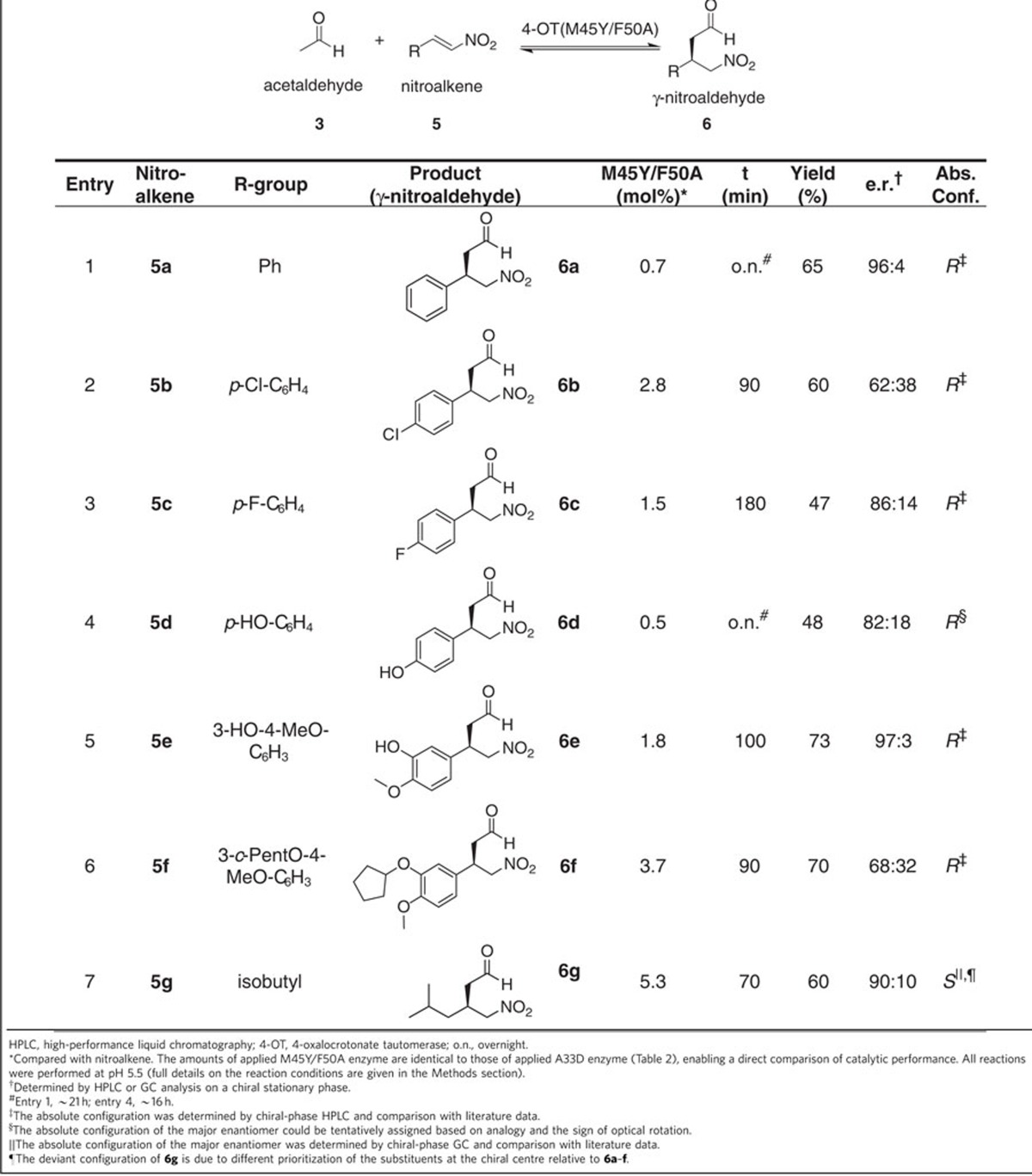
4-OT(M45Y/F50A)-catalysed acetaldehyde addition to nitroalkenes 5a–5g.
