# Supplementary material for: Using mutability landscapes of a promiscuous tautomerase to guide the engineering of enantioselective Michaelases
Source: Nat Commun. 2016 Mar 8;7:10911. doi: 10.1038/ncomms10911 (PMC4786785; doi:10.1038/ncomms10911)
Supplement: Supplementary Information — Supplementary Figures 1-31, Supplementary Tables 1-9, Supplementary Discussion and Supplementary References [file ncomms10911-s1.pdf]

Supplementary Figures

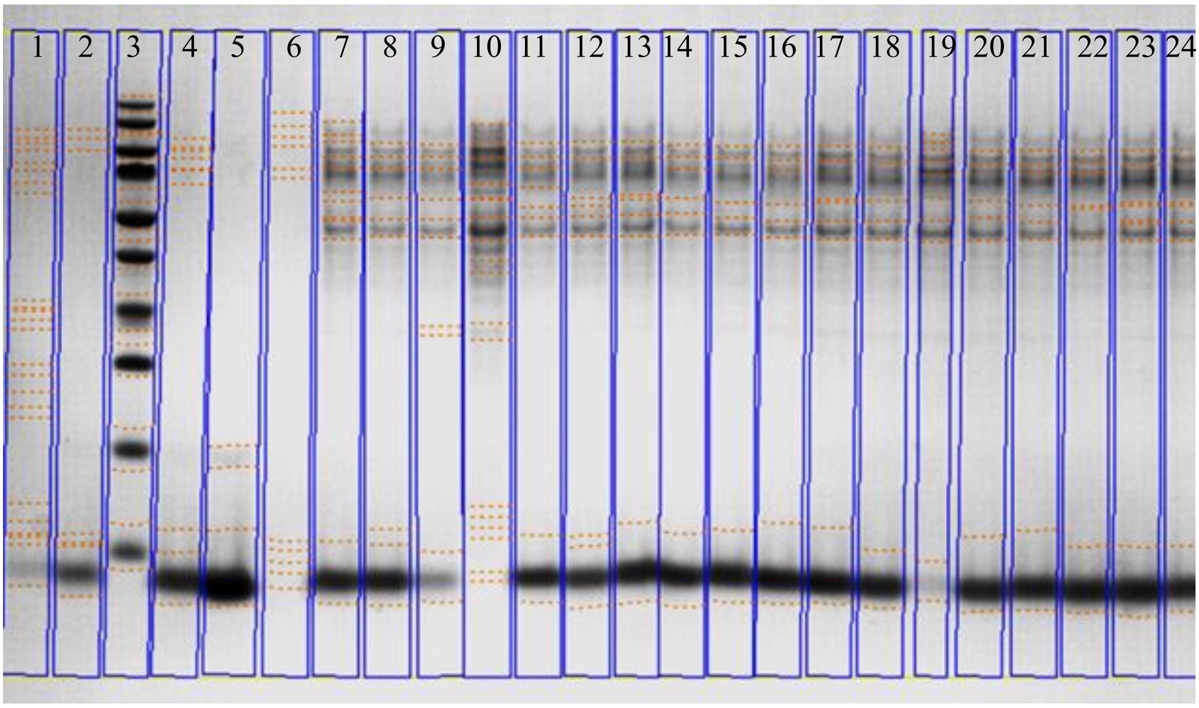

Supplementary Figure 1 | Picture of a typical SDS-page gel used in the quantitative densitometric analysis to determine the 4-OT concentration in the cell-free extracts. Lanes 1, 2, 4 and 5 contain calibration samples of known amounts of purified wild-type 4-OT (resp. 0.5, 1, 2.5 and 5 μg). Lane 3 contains the PageRuler^{TM} prestained protein ladder (Thermo Scientific). Lanes 7-24 contain CFE samples of *E. coli* BL21 (DE3) cultures, each expressing a different 4-OT variant. The blue boxes represent lanes and dashed orange lines indicate an area in which a protein band is detected by the program 'GeneTools'. This program quantifies each detected protein band, based on the size and intensity.

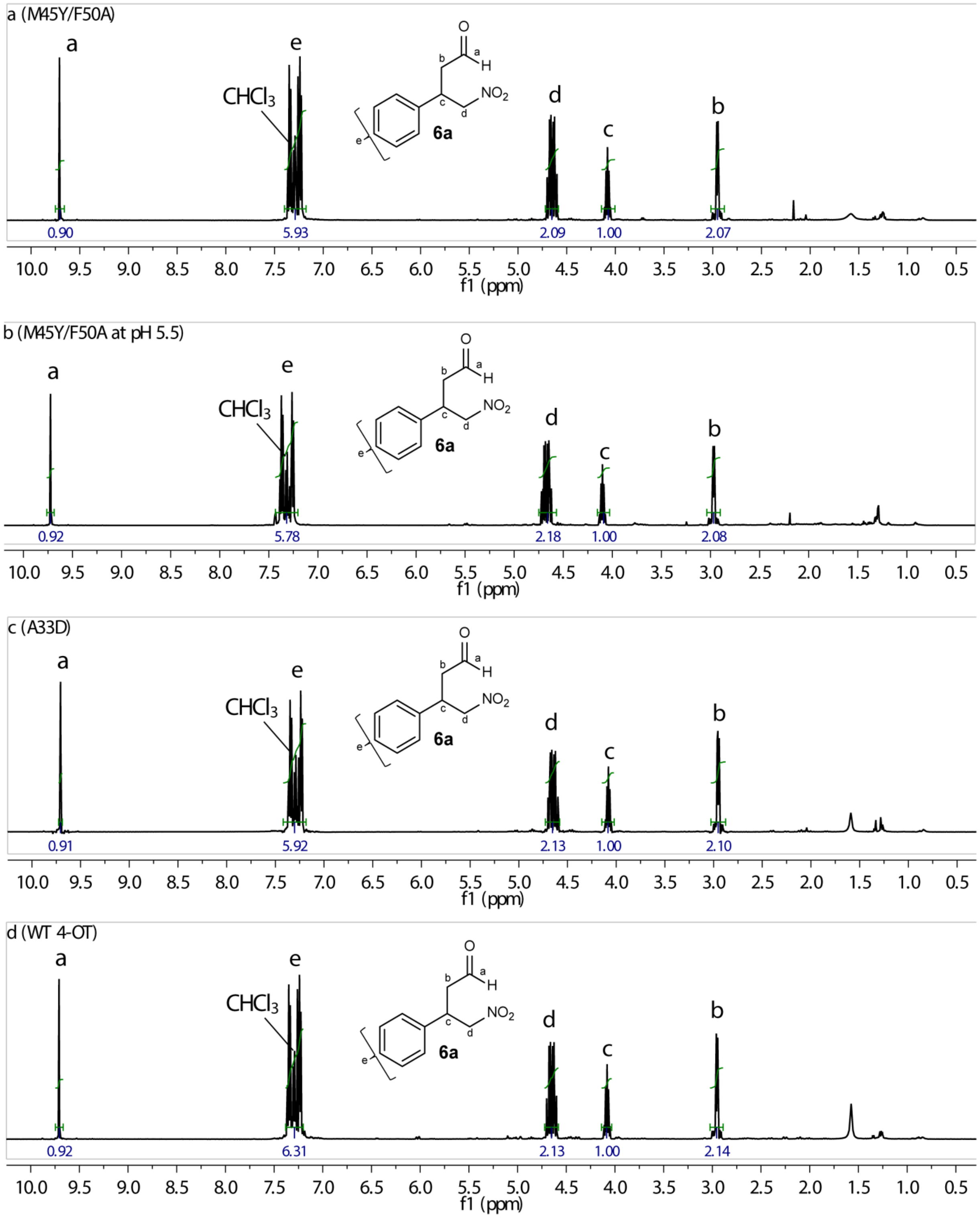

Supplementary Figure 2 | ^{1}H NMR spectra of enzymatically obtained **6a**. Product **6a** was obtained from the semi-preparative scale reactions listed in **Table 1** and shown in **Fig. 4a**. Spectra a, c and d were obtained from the reactions performed at pH 7.3; these products were purified using flash chromatography before the ^{1}H NMR spectra were recorded. Spectrum b was obtained from a reaction performed at pH 5.5 with 4-OT mutant M45Y/F50A; no flash purification step was performed on this sample before the ^{1}H NMR spectrum was recorded. The spectroscopic data are in agreement with the literature.[1]

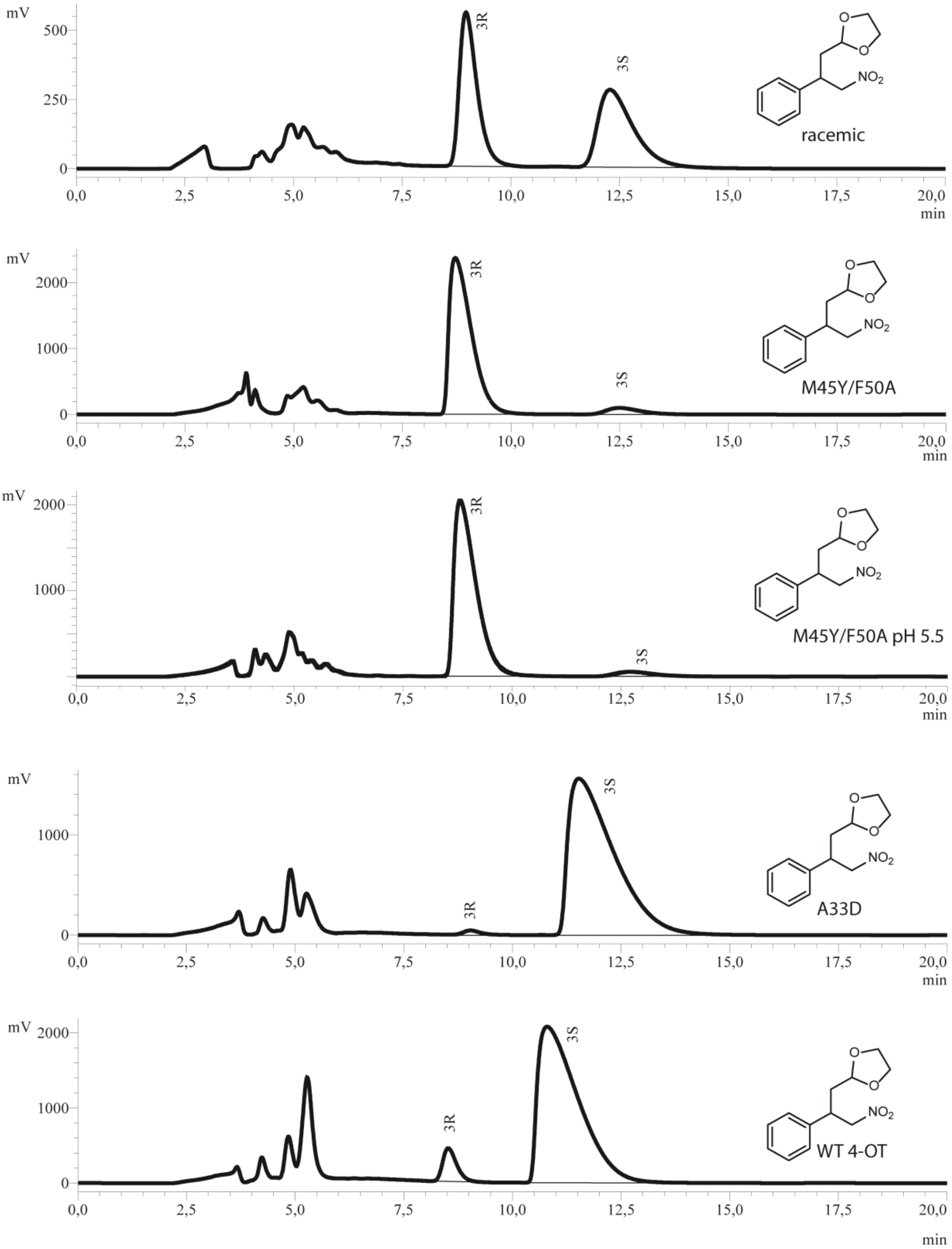

Supplementary Figure 3 | HPLC chromatograms of derivatised racemic **6a** and derivatised enzymatically obtained **6a**. The enzymatically obtained **6a** was obtained after the semi-preparative scale reactions listed in **Table 1** and shown in **Fig. 4a**. The ^{1}H NMR spectra of the products are shown in **Supplementary Figure 2**.

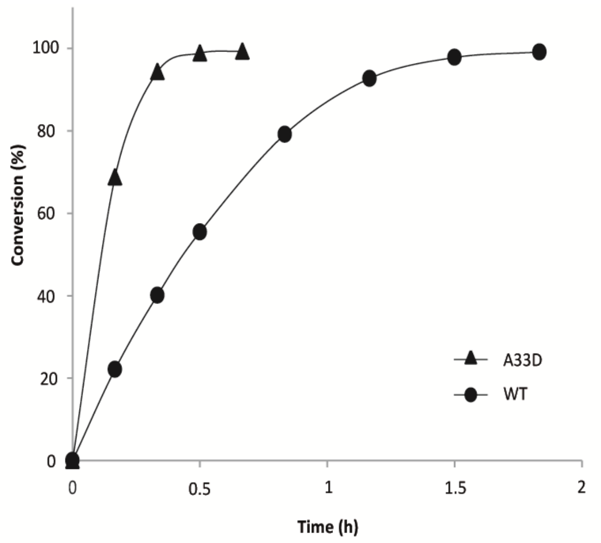

Supplementary Figure 4 | Progress curves of the Michael-type addition of **3** (50 mM) to **5a** (2 mM, 18 mg) catalyzed (1.4 mol%) by wild-type 4-OT or 4-OT A33D. The reactions were carried out in buffer [20 mM NaH_2PO_4 / 10% EtOH (v/v)] at pH 6.5. These progress curves are derived from the preparative scale reactions listed in **Table 1** (entries 4 and 5).

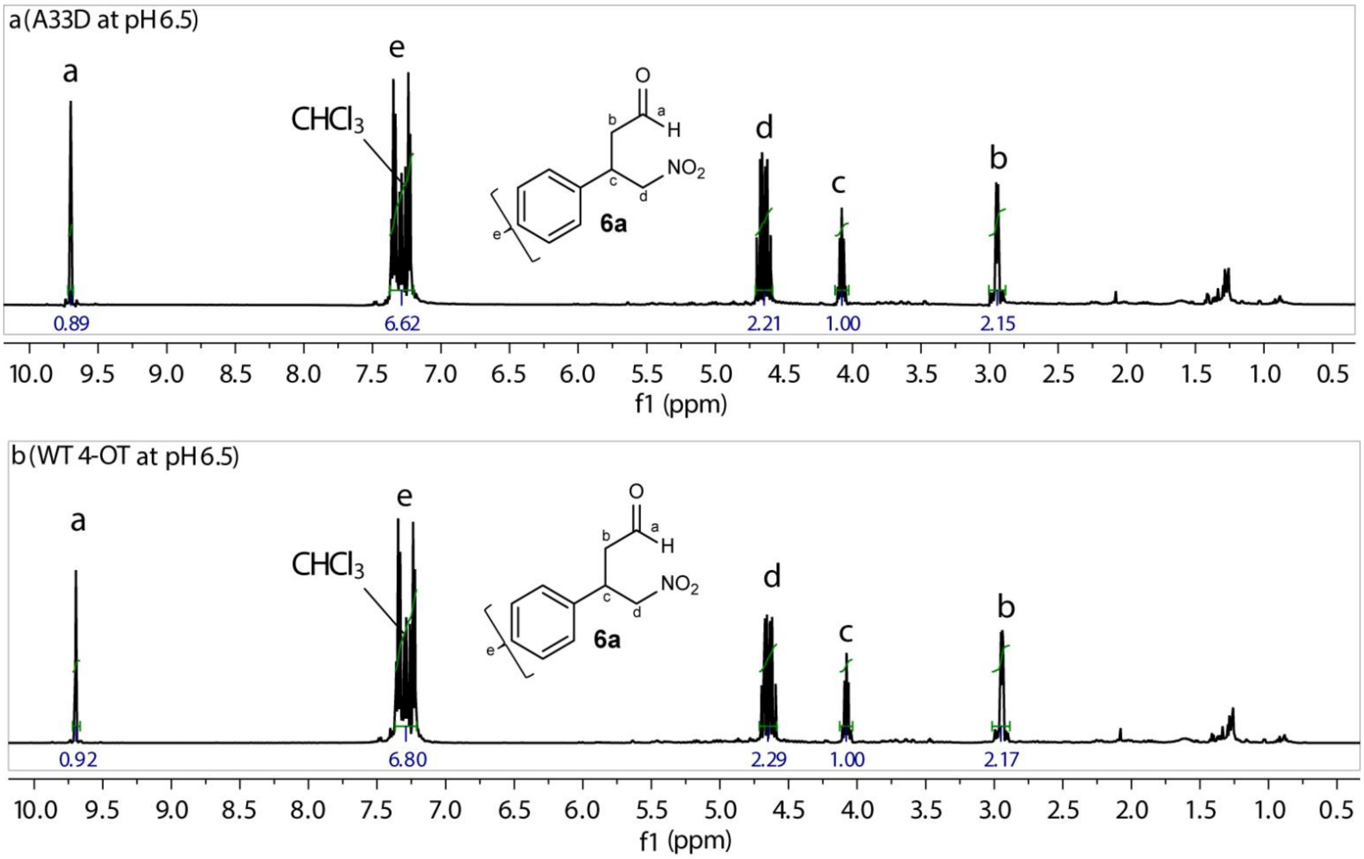

Supplementary Figure 5 | ^{1}H NMR spectra of enzymatically obtained **6a**. Product **6a** was obtained from the semi-preparative scale reactions listed in **Table 1** (entries 4 and 5) and shown in **Supplementary Figure 4**. No flash purification step was performed on these samples before the ^{1}H NMR spectra were recorded. The spectroscopic data are in agreement with the literature.[1]

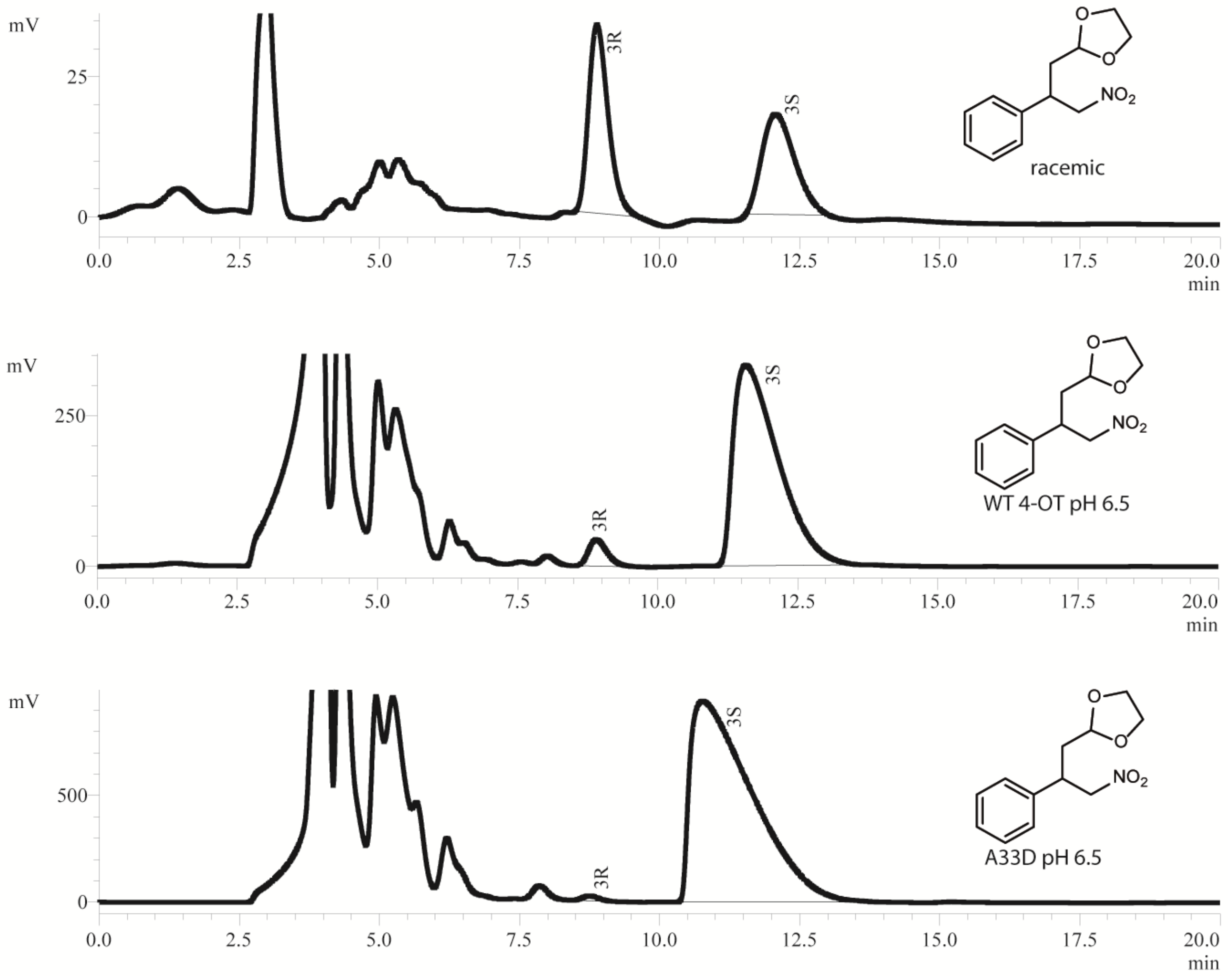

Supplementary Figure 6 | HPLC chromatograms of derivatised racemic **6a** and derivatised enzymatically obtained **6a**. The enzymatically obtained **6a** was obtained after the semi-preparative scale reactions (conducted at pH 6.5) listed in **Table 1** (entries 4 and 5) and shown in **Supplementary Figure 4**. The ^{1}H NMR spectra of the products are shown in **Supplementary Figure 5**.

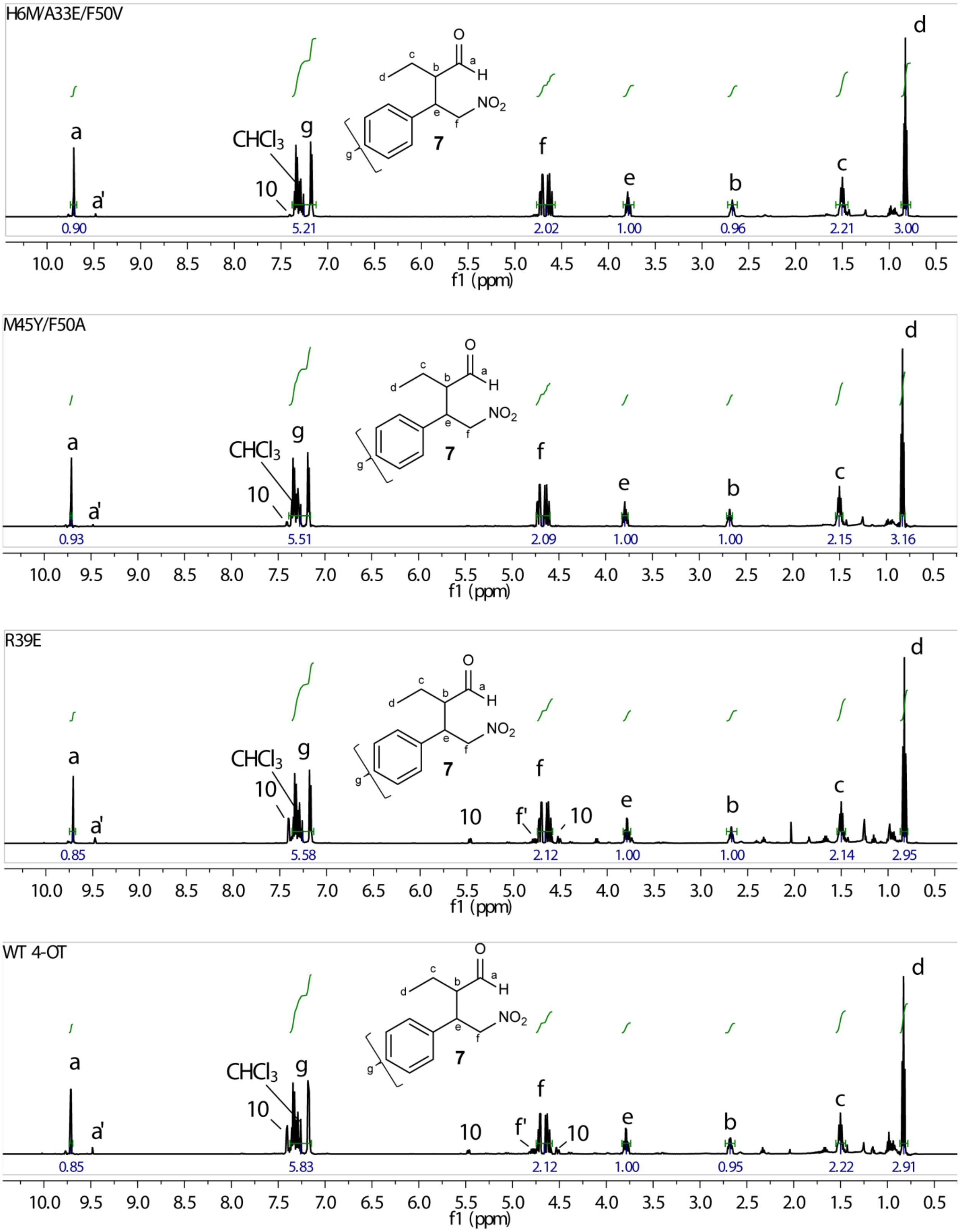

Supplementary Figure 7 | ^{1}H NMR spectra of enzymatically obtained **7**. The spectra of product **7**, which were obtained from the semi-preparative scale reactions listed in **Table 1** and shown in **Fig. 4b**, are in agreement with literature data.[2,3] The peaks assigned with a′ and f′ correspond to the *anti*-diastereoisomer of **7**. 2-Nitro-1-phenylethanol (**10**) is the product of non-enzymatic water addition to **5a**.[4]

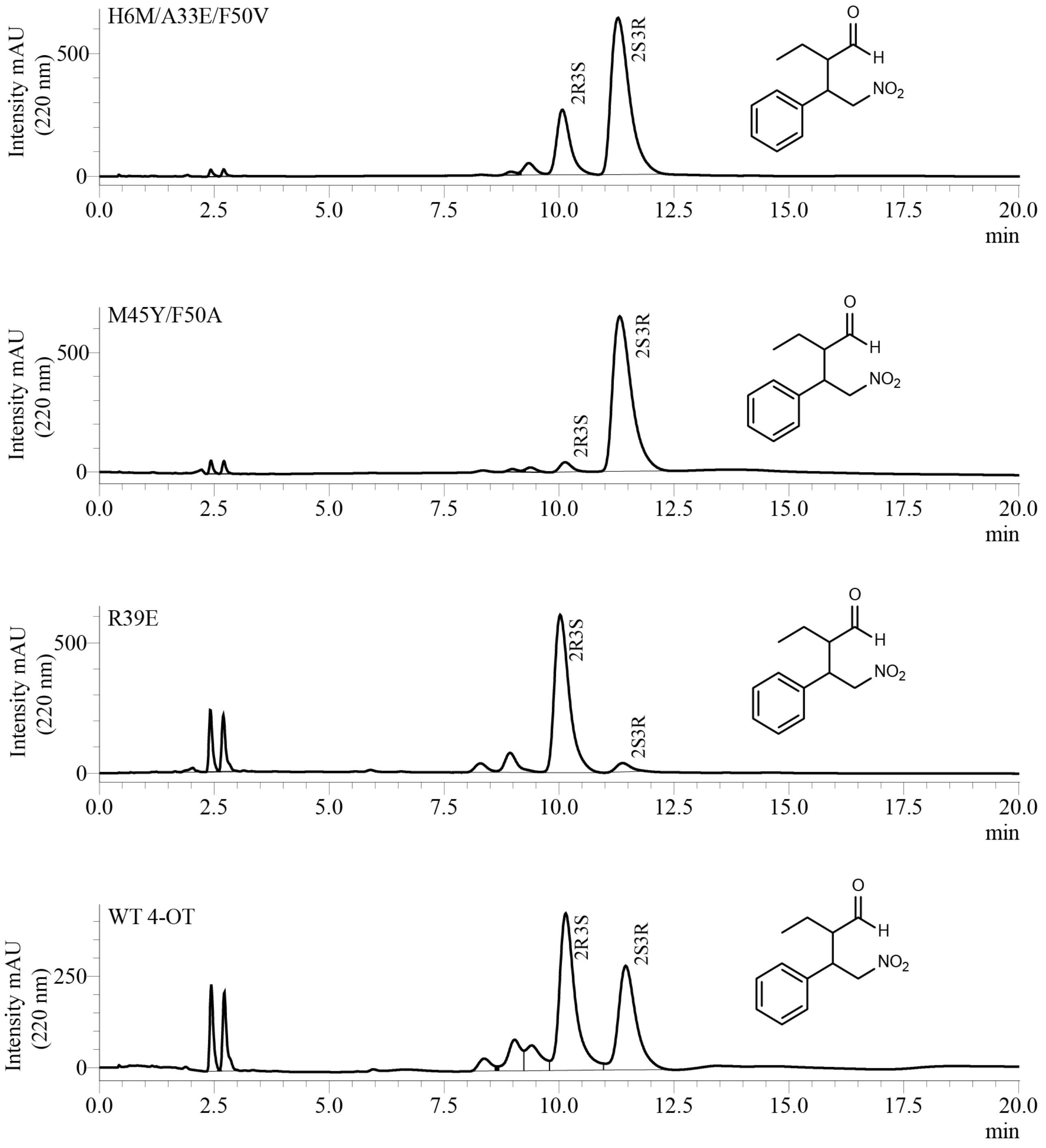

Supplementary Figure 8 | Chromatograms for e.r. determination of enzymatically produced **7**. The enzymatically obtained **7** was obtained after the semi-preparative scale reactions listed in **Table 1** and shown in **Fig. 4b.** The ^{1}H NMR spectra of the products are shown in **Supplementary Figure 7**.

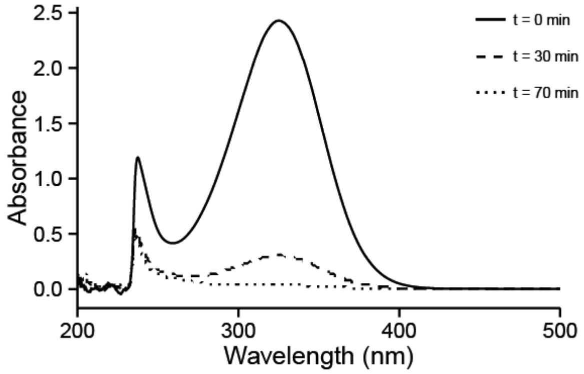

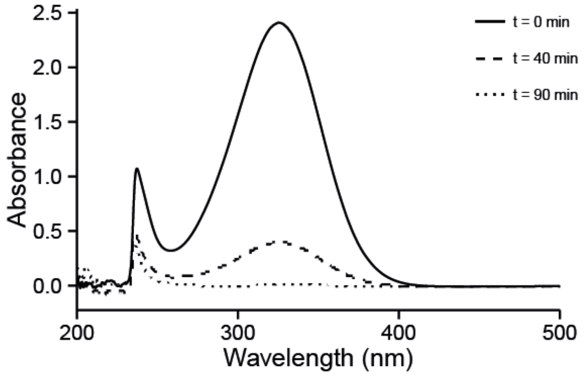

Supplementary Figure 9 | UV-spectra showing the conversion of the nitroalkene **5b**, upon incubation with **3** and either 4-OT A33D (left) or 4-OT M45Y/F50A (right) in 20 mM NaH_2PO_4/45% DMSO (v/v) at pH 5.5.

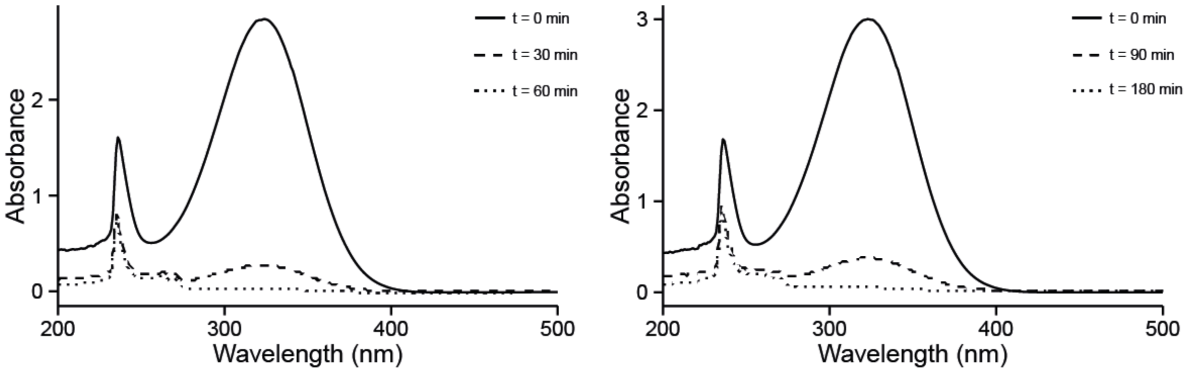

Supplementary Figure 10 | UV-spectra showing the conversion of the nitroalkene **5c**, upon incubation with **3** and either 4-OT A33D (left) or 4-OT M45Y/F50A (right) in 20 mM NaH_2PO_4/40% DMSO (v/v) at pH 5.5.

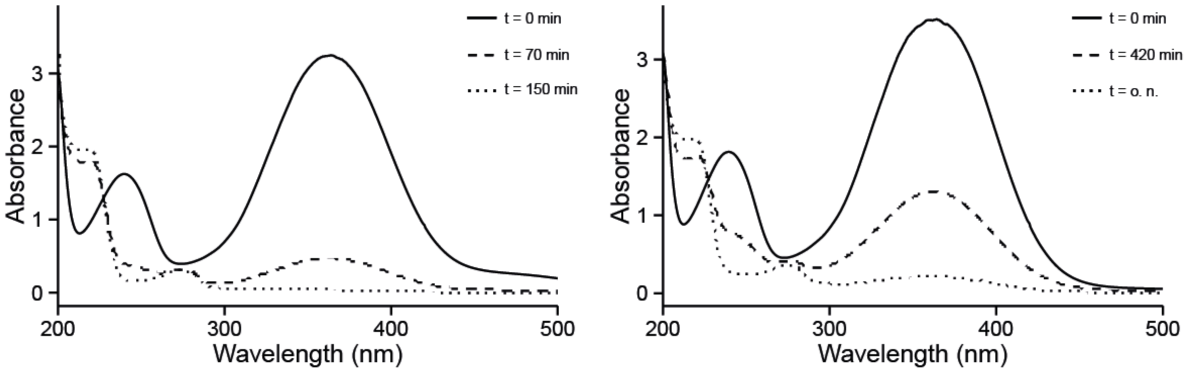

Supplementary Figure 11 | UV-spectra showing the conversion of the nitroalkene **5d**, upon incubation with **3** and either 4-OT A33D (left) or 4-OT M45Y/F50A (right) in 20 mM NaH_2PO_4/10% EtOH (v/v). The pH of the buffer was pH 6.5 for the reaction with 4-OT A33D and pH 5.5 for the reaction with 4-OT M45Y/F50A.

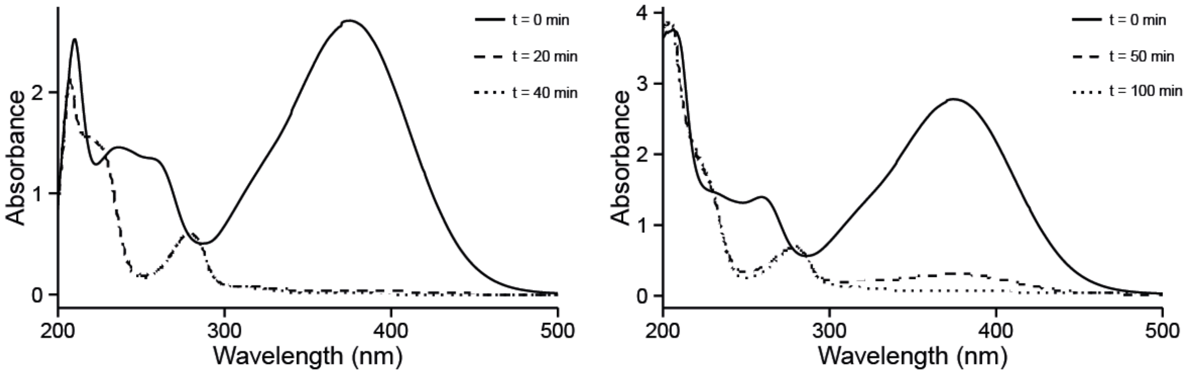

Supplementary Figure 12 | UV-spectra showing the conversion of the nitroalkene **5e**, upon incubation with **3** and either 4-OT A33D (left) or 4-OT M45Y/F50A (right) in 20 mM NaH_2PO_4/10% EtOH (v/v). The pH of the buffer was pH 6.5 for the reaction with 4-OT A33D and pH 5.5 for the reaction with 4-OT M45Y/F50A.

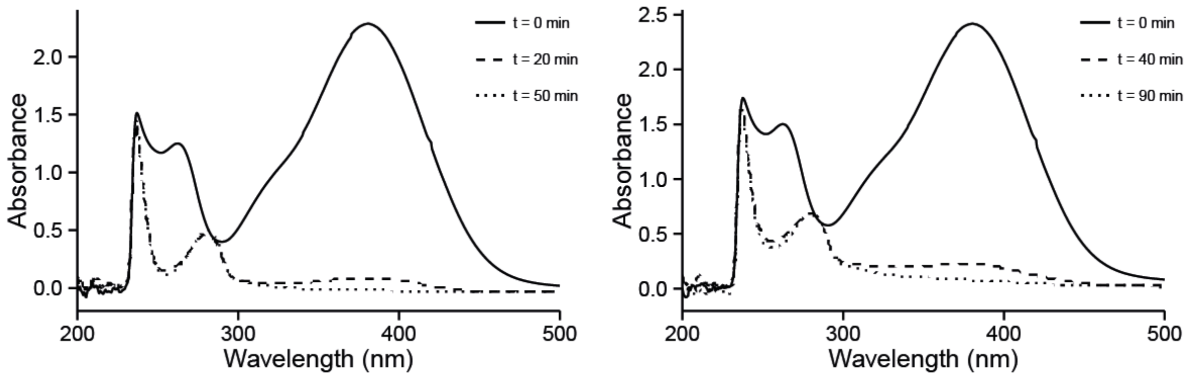

Supplementary Figure 13 | UV-spectra showing the conversion of the nitroalkene **5f**, upon incubation with **3** and either 4-OT A33D (left) or 4-OT M45Y/F50A (right) in 20 mM NaH_2PO_4/40% DMSO (v/v) at pH 5.5.

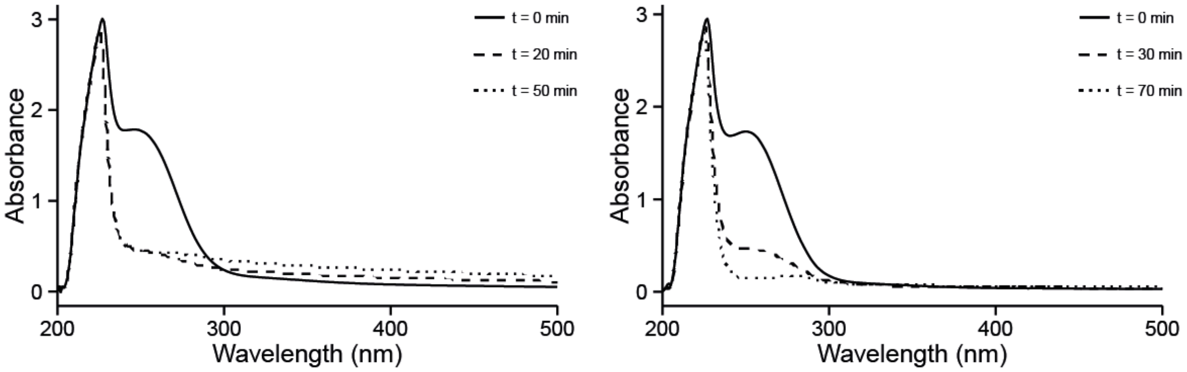

Supplementary Figure 14 | UV-spectra showing the conversion of the nitroalkene **5g**, upon incubation with **3** and either 4-OT A33D (left) or 4-OT M45Y/F50A (right) in 20 mM NaH_2PO_4/5% DMSO (v/v) at pH 5.5.

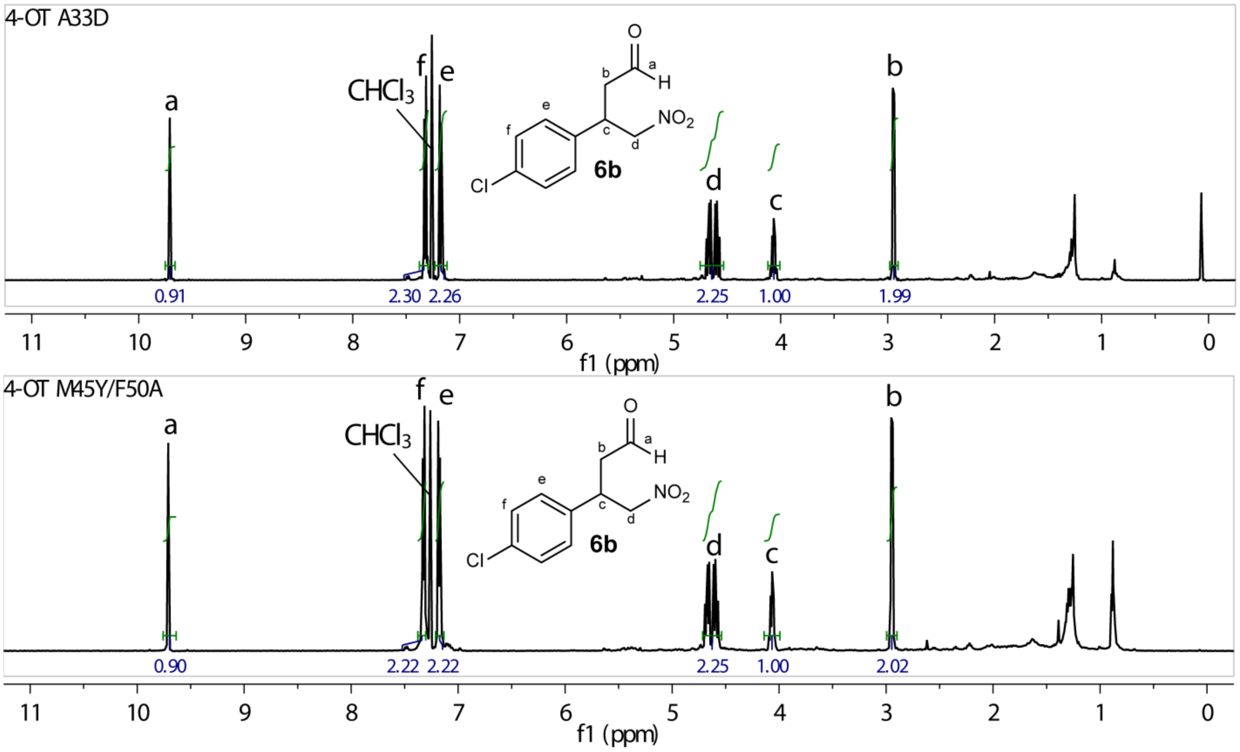

Supplementary Figure 15 | ^{1}H NMR spectra of **6b** obtained with 4-OT A33D (top) or 4-OT M45Y/F50A (bottom).

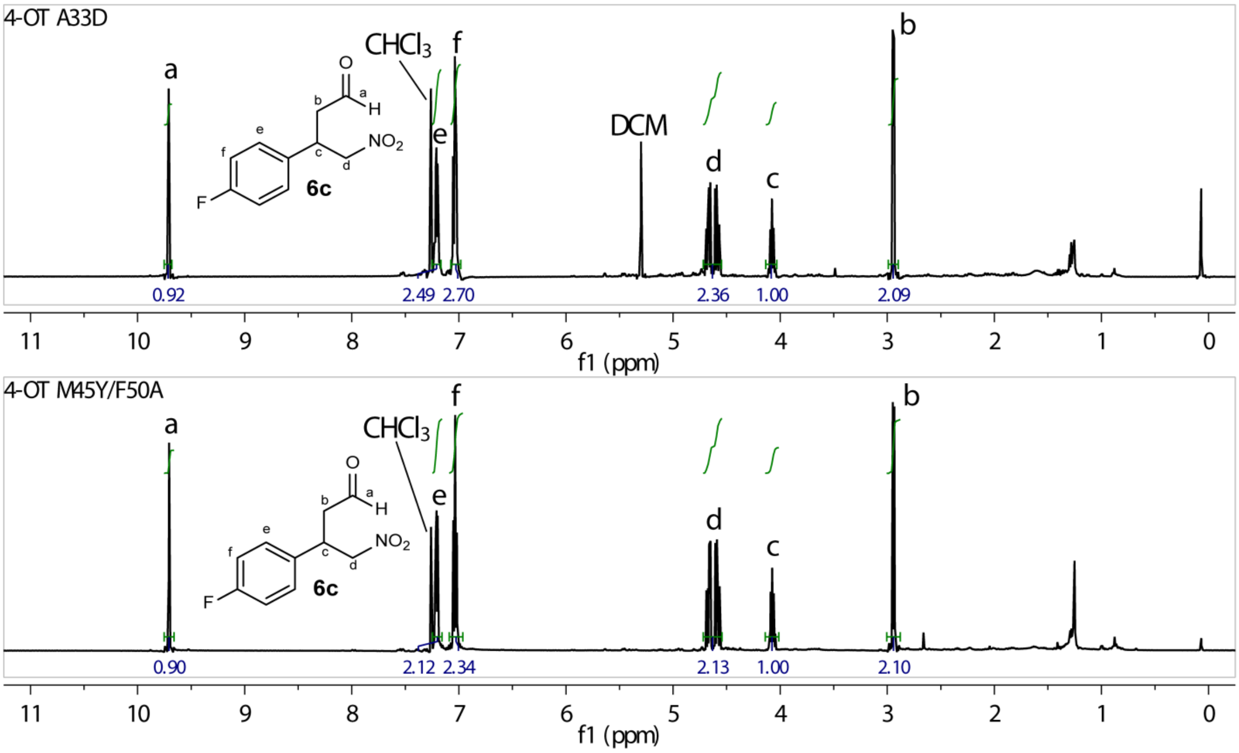

Supplementary Figure 16 | ^{1}H NMR spectra of **6c** obtained with 4-OT A33D (top) or 4-OT M45Y/F50A (bottom).

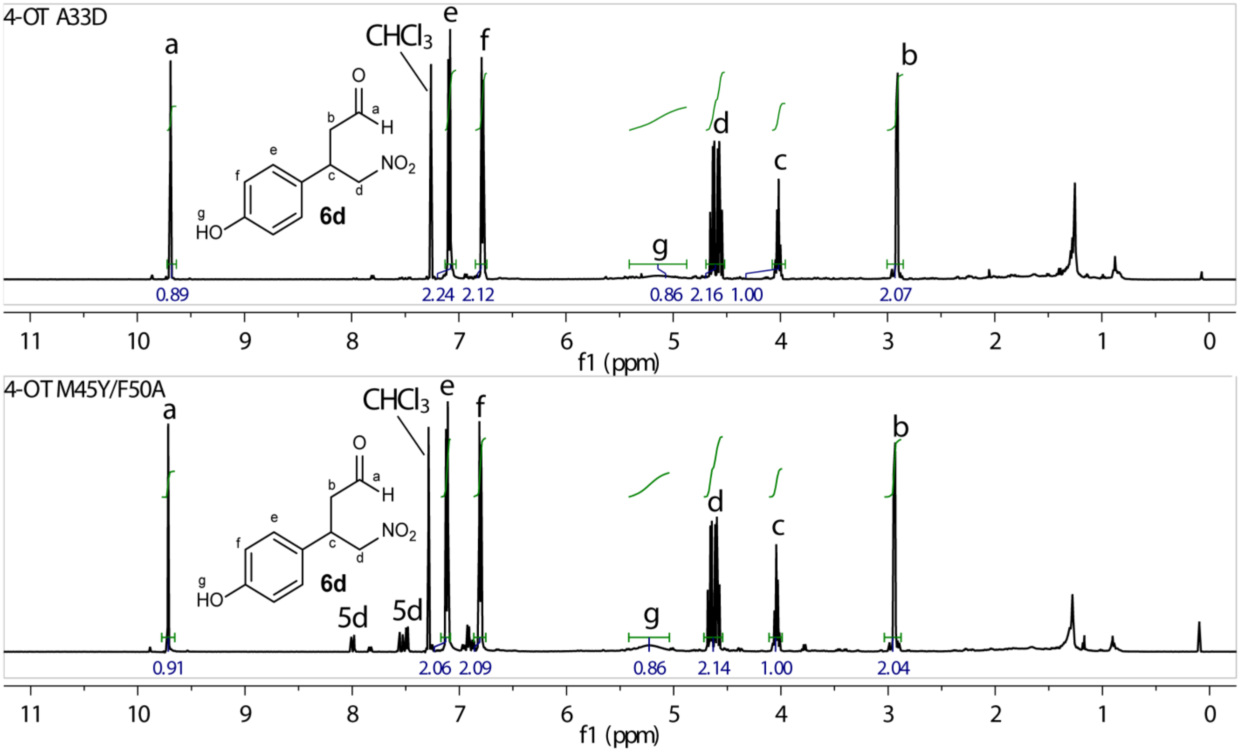

Supplementary Figure 17 | ^{1}H NMR spectra of **6d** obtained with 4-OT A33D (top) or 4-OT M45Y/F50A (bottom); **5d** is residual starting material.

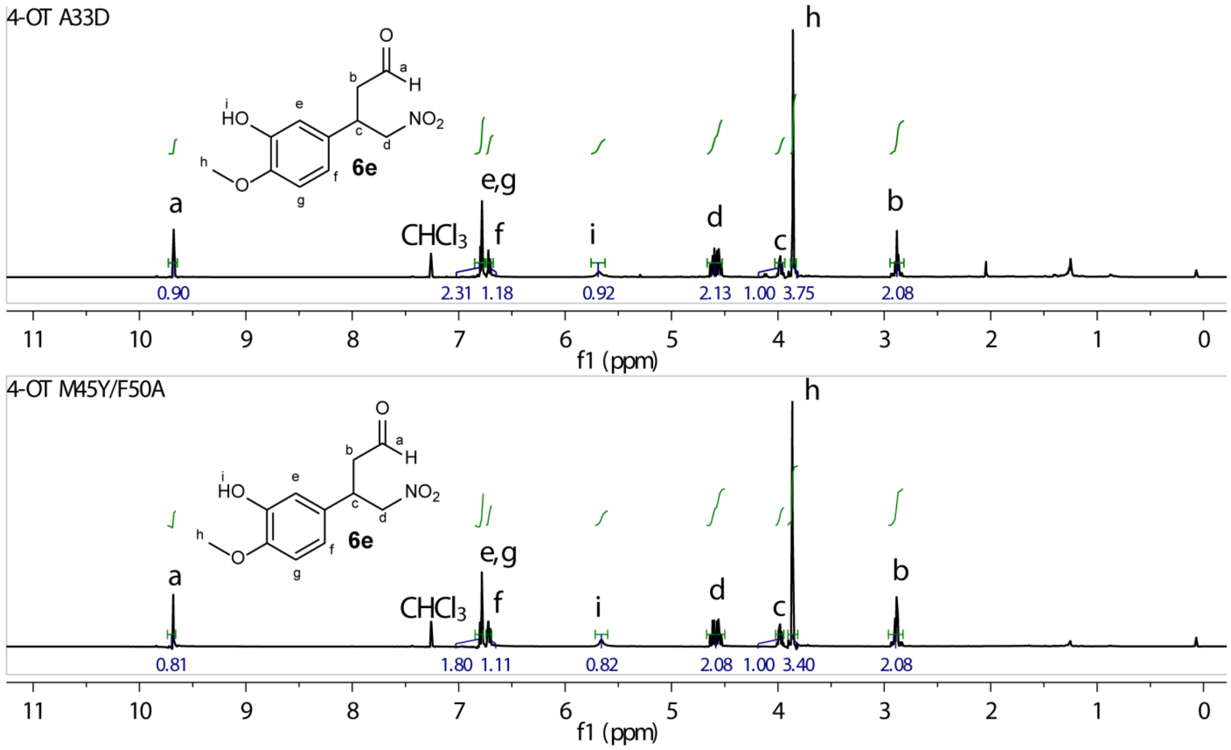

Supplementary Figure 18 | ^{1}H NMR spectra of **6e** obtained with 4-OT A33D (top) or 4-OT M45Y/F50A (bottom).

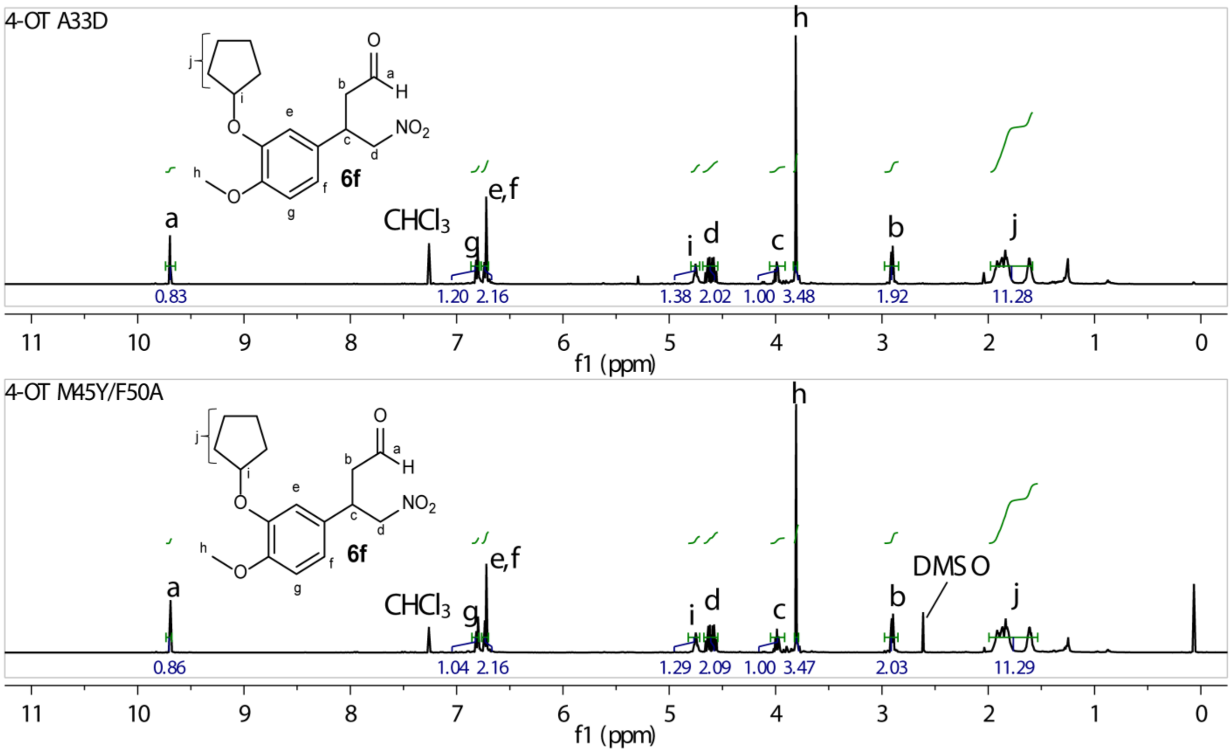

Supplementary Figure 19 | ^{1}H NMR spectra of **6f** obtained with 4-OT A33D (top) or 4-OT M45Y/F50A (bottom).

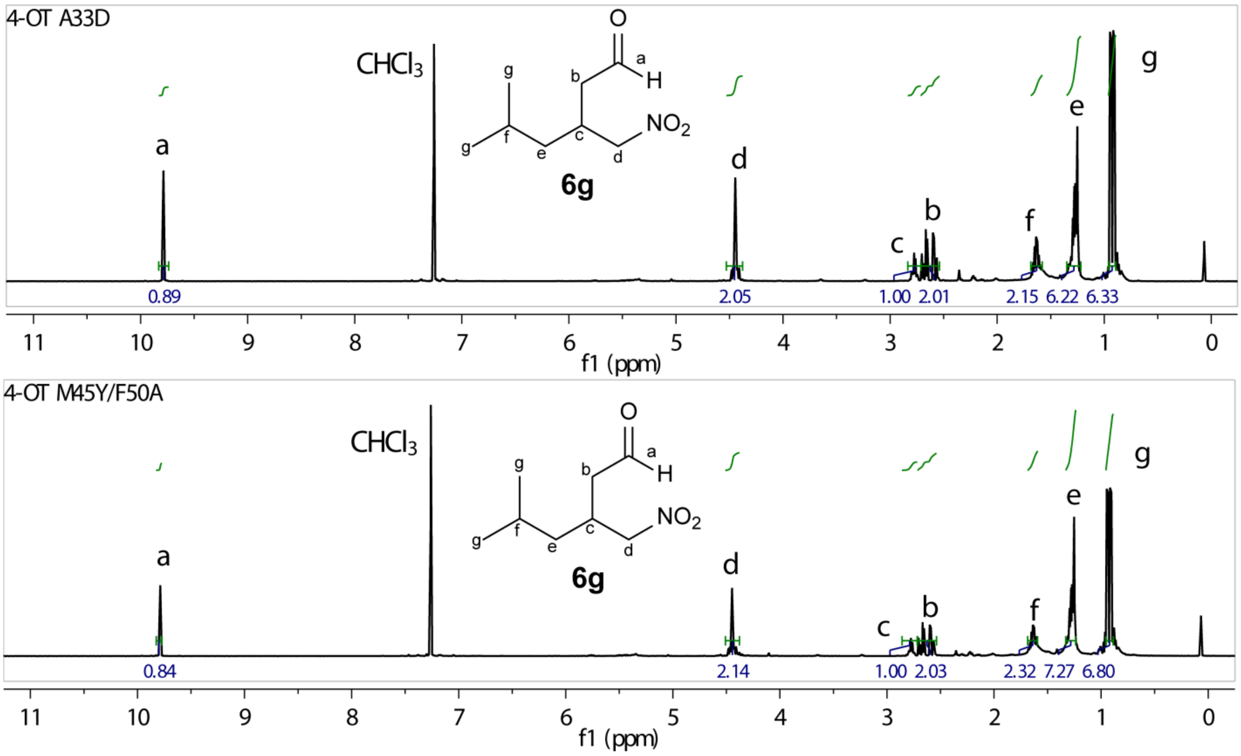

Supplementary Figure 20 | ^{1}H NMR spectra of **6g** obtained with 4-OT A33D (top) or 4-OT M45Y/F50A (bottom).

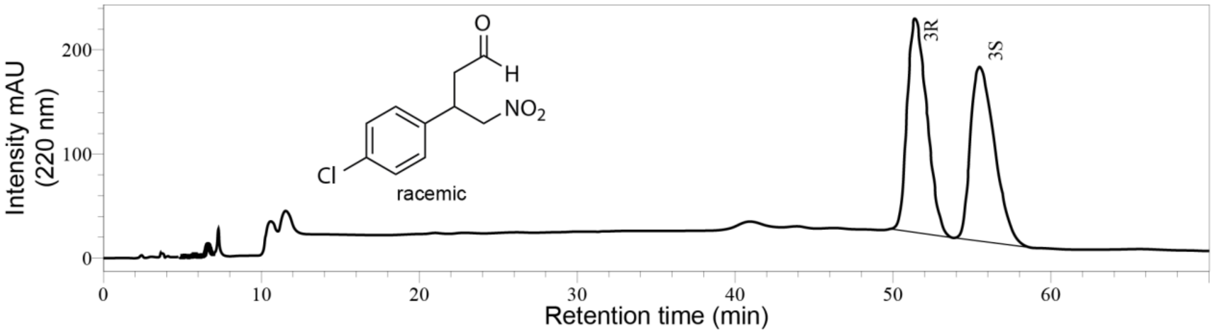

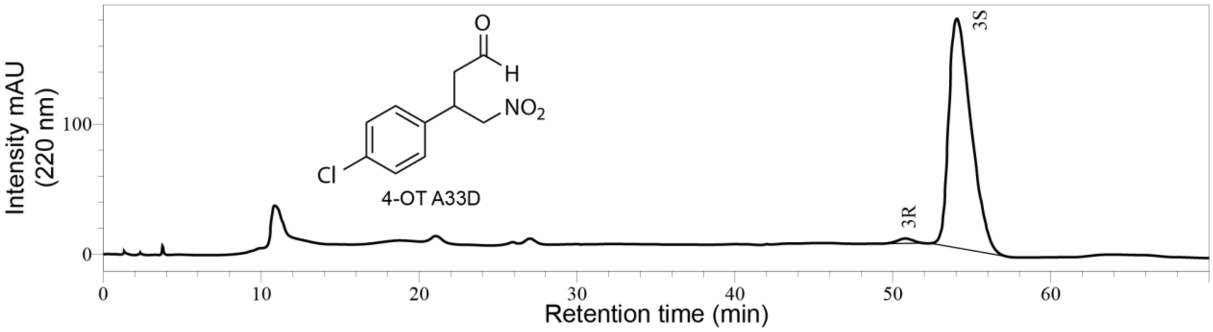

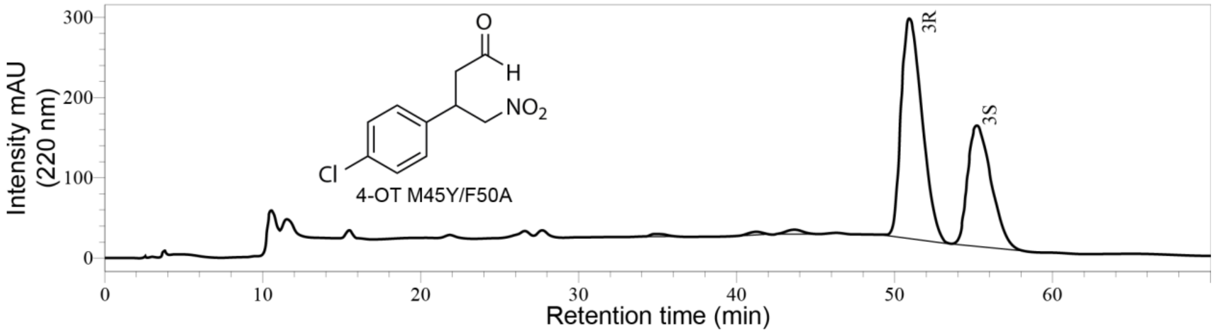

Supplementary Figure 21 | HPLC chromatograms of racemic **6b** and enzymatically obtained **6b**.

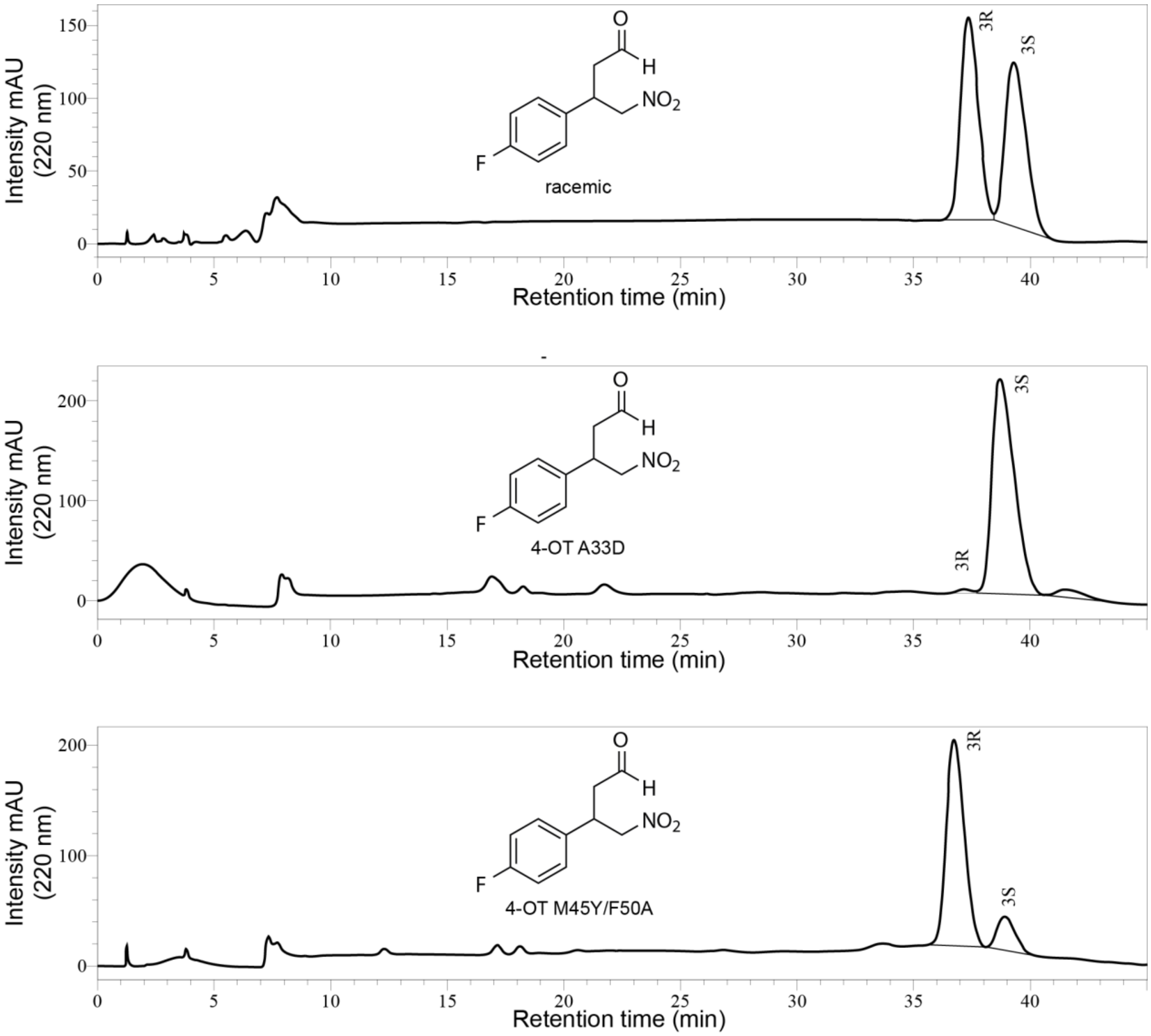

Supplementary Figure 22 | HPLC chromatograms of racemic **6c** and enzymatically obtained **6c**.

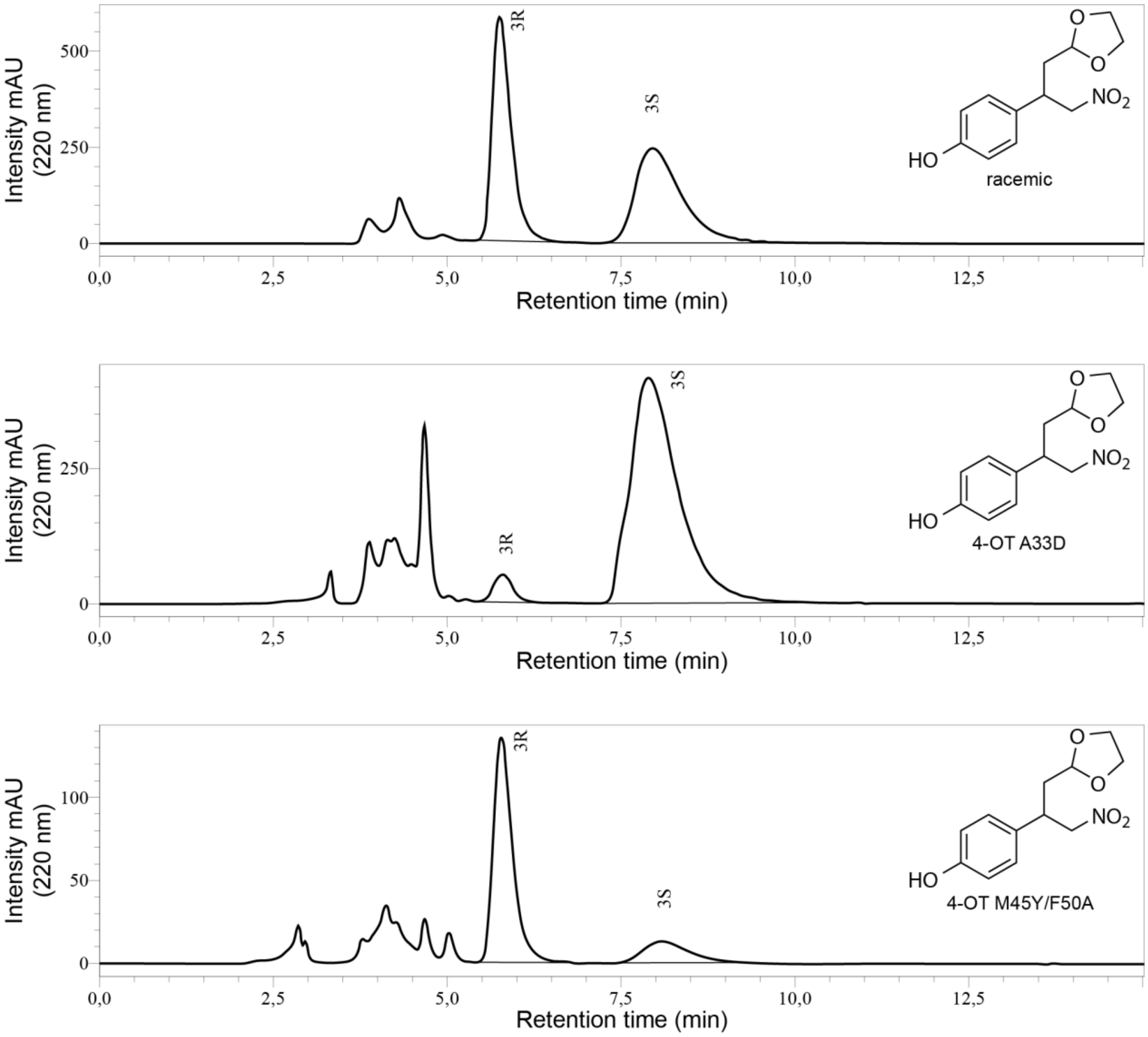

Supplementary Figure 23 | HPLC chromatograms of derivatised racemic **6d** and derivatised enzymatically obtained **6d**.

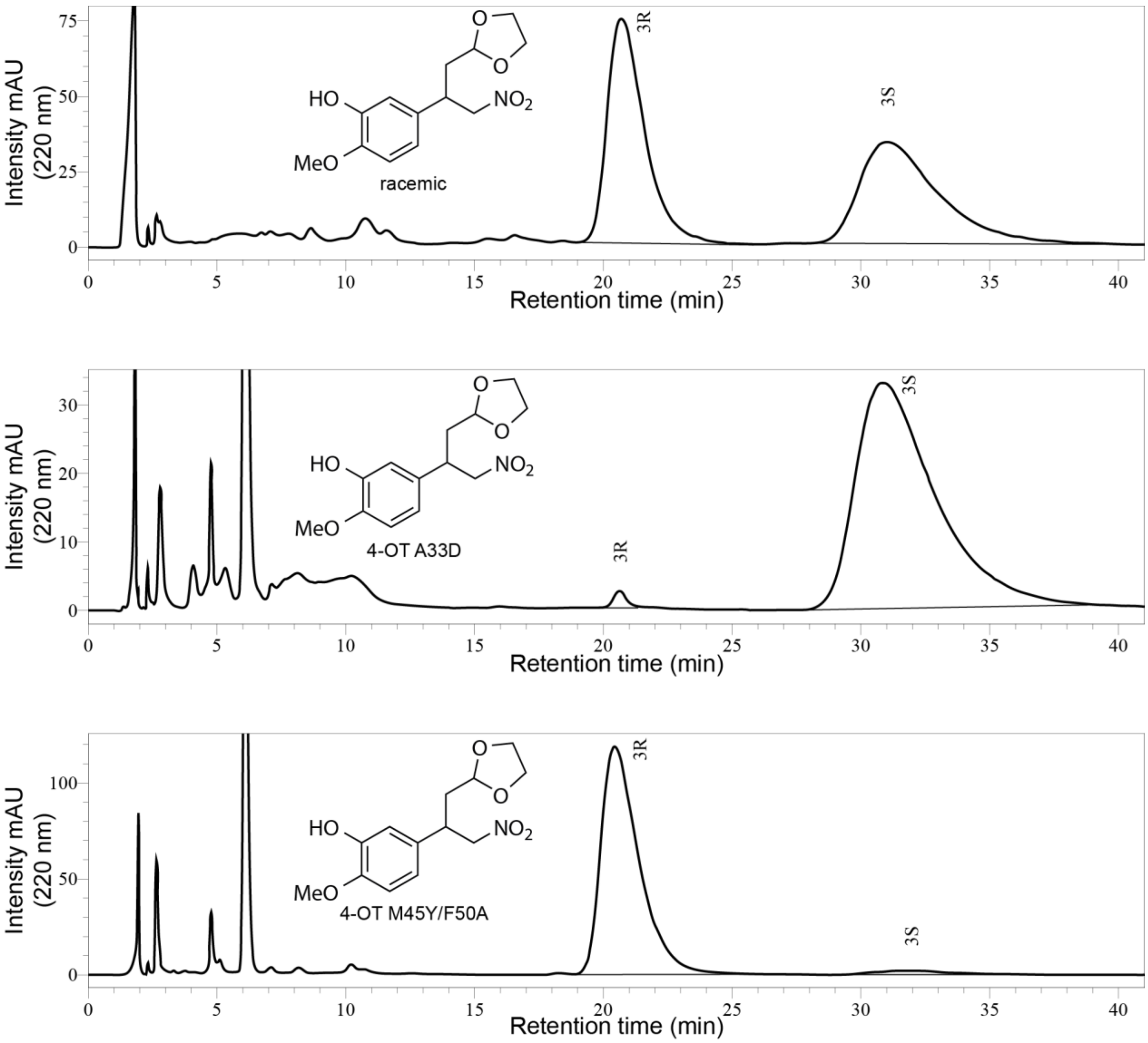

Supplementary Figure 24 | HPLC chromatograms of derivatised racemic **6e** and derivatised enzymatically obtained **6e**.

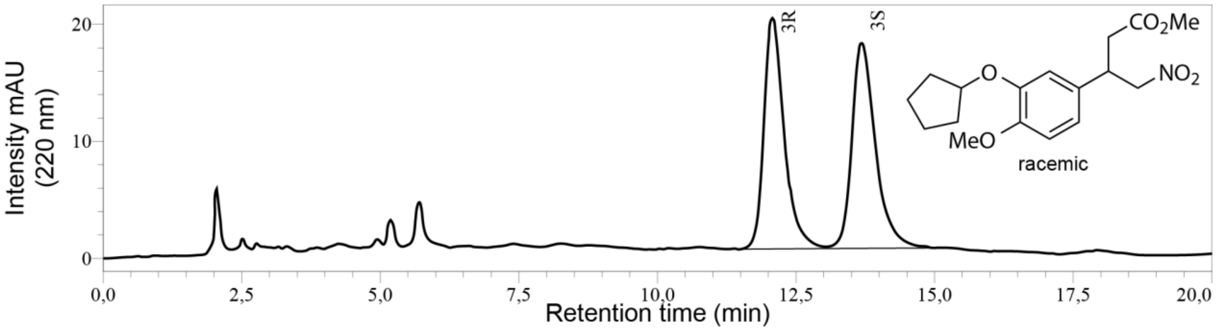

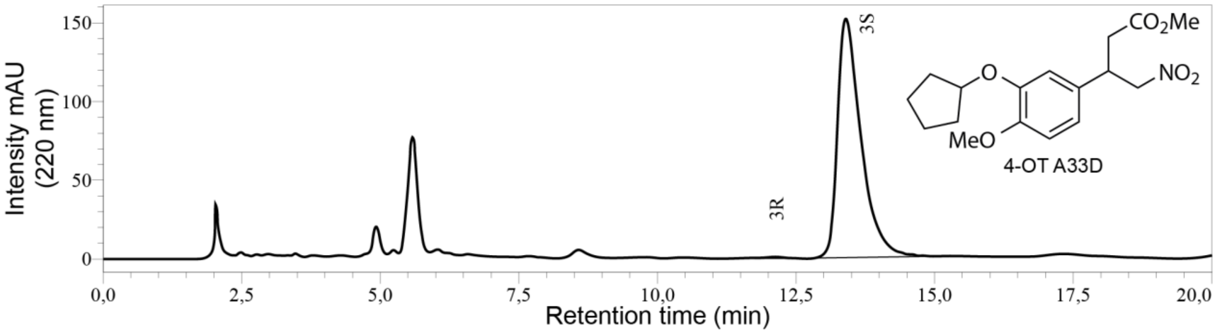

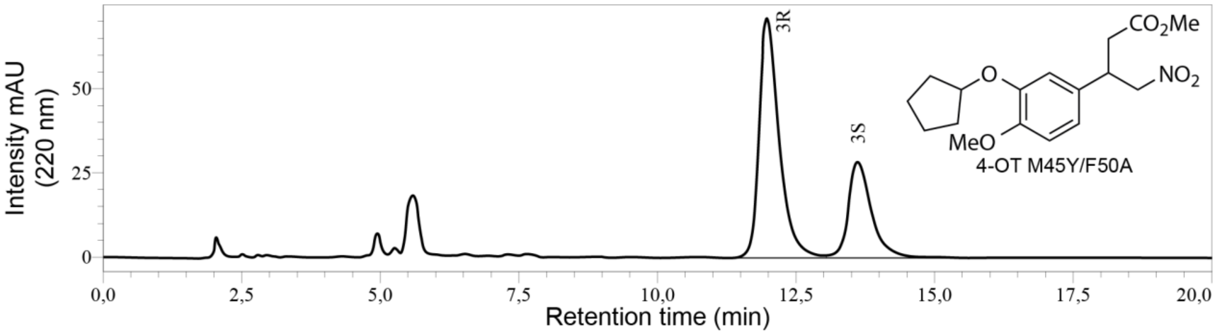

Supplementary Figure 25 | HPLC chromatograms of derivatised racemic **6f** and derivatised enzymatically obtained **6f**.

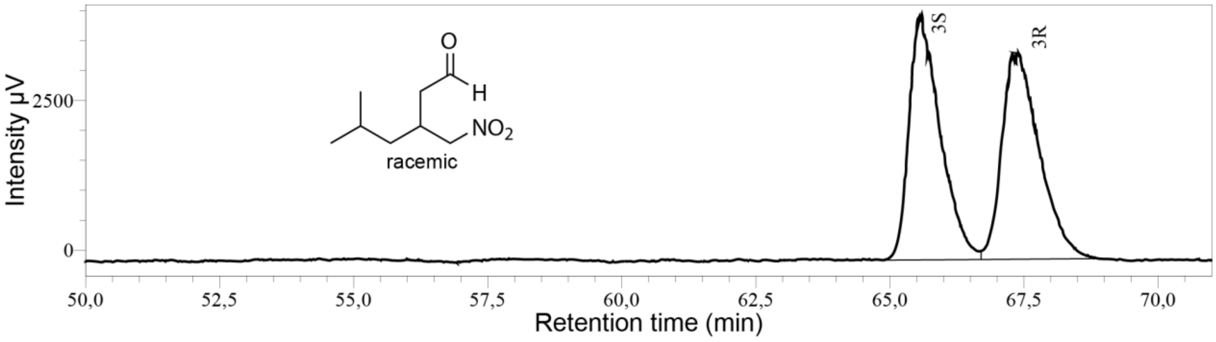

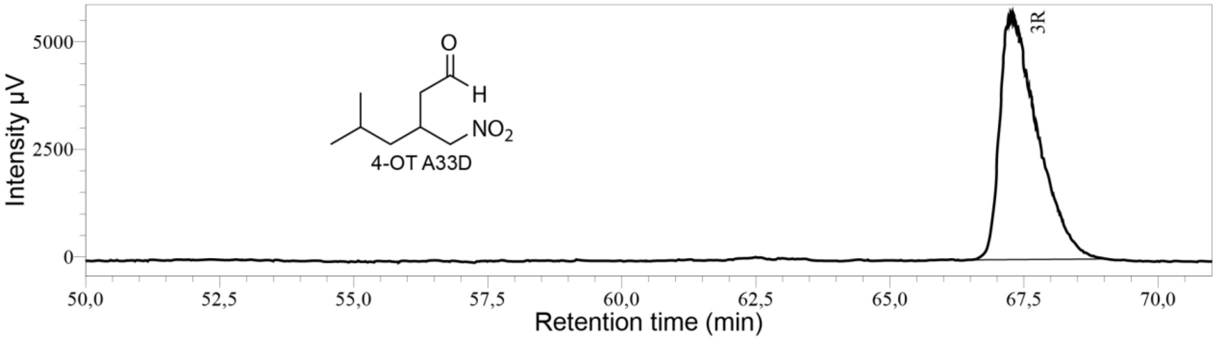

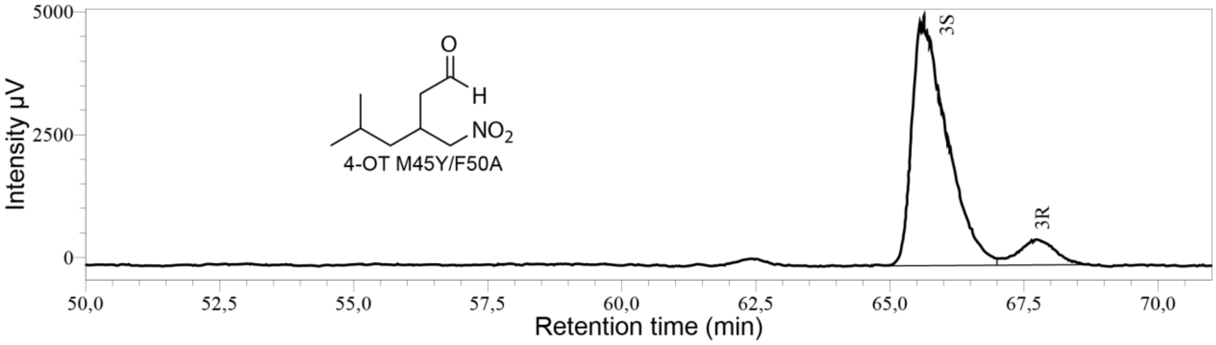

Supplementary Figure 26 | GC chromatograms of racemic **6g** and enzymatically obtained **6g**.

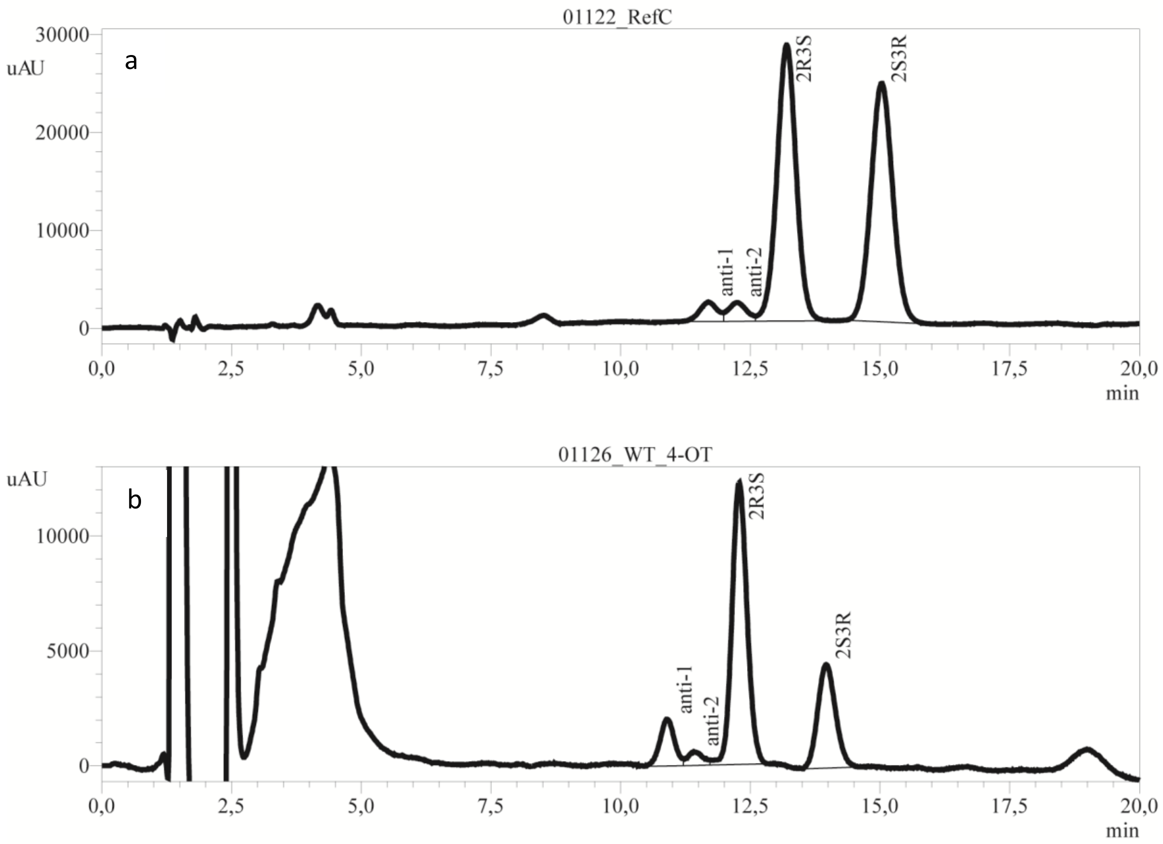

Supplementary Figure 27 | Chiral phase HPLC analysis of **7** and assignment of absolute configuration. **a,** Chromatogram of chemically synthesized racemic **7**. **b,** Chromatogram of enzymatically produced **7**.

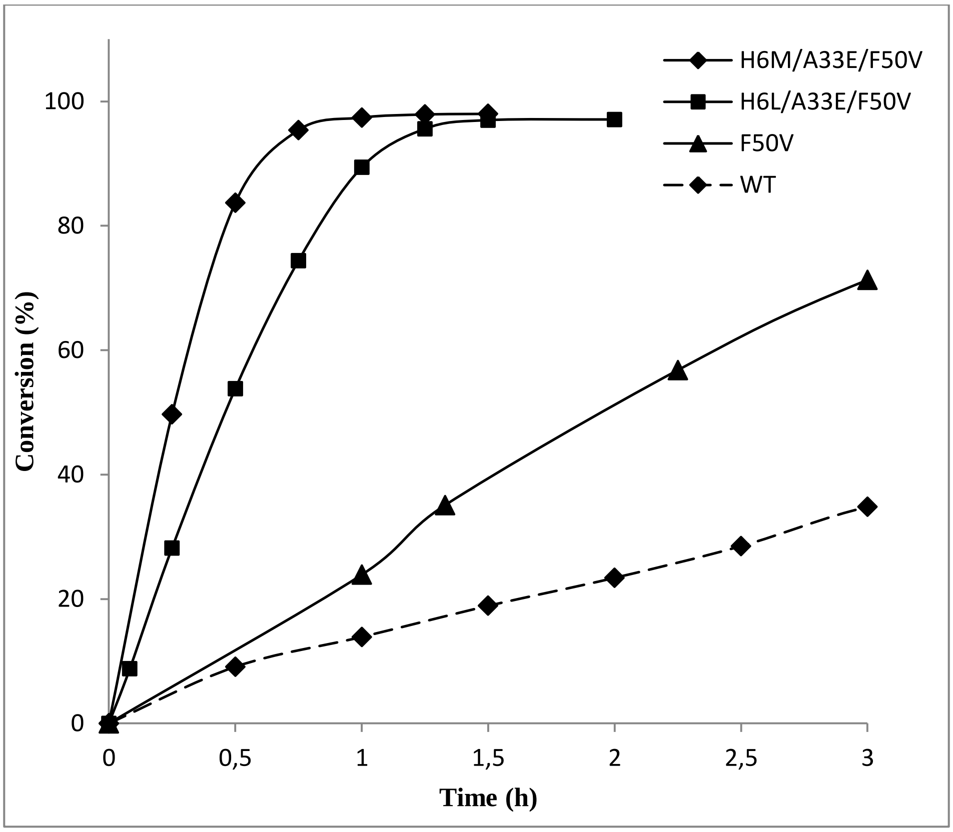

Supplementary Figure 28 | Progress curves of the Michael-type addition of **4** to **5a** catalysed by wild-type 4-OT and 4-OT variants.

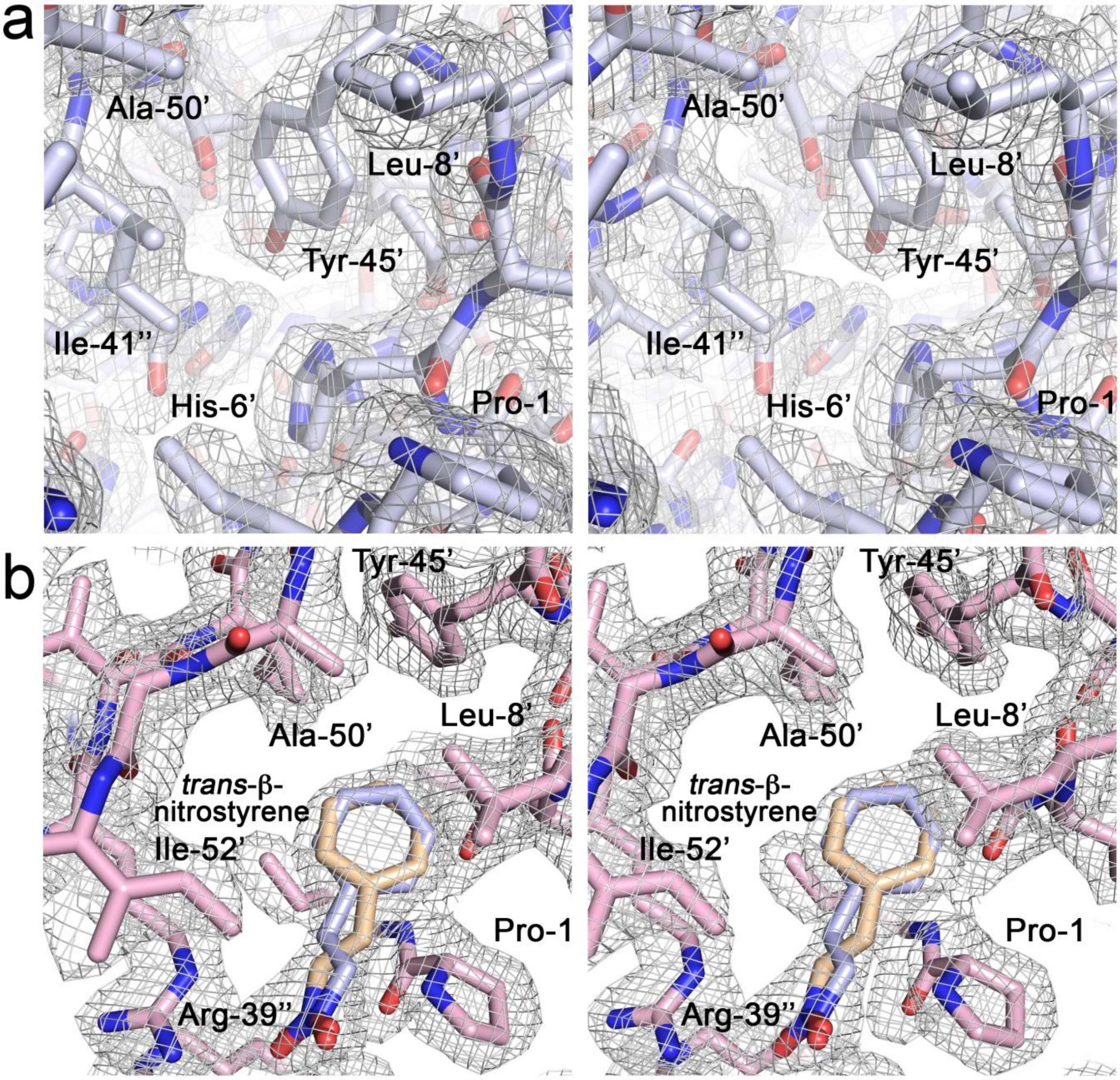

Supplementary Figure 29 | Stereo diagrams of the electron density at the Pro-1 pocket in the 4-OT mutant structures. **a,** The Pro-1 pocket in the substrate-free M45Y/F50A mutant (PDB code – 5CLN) and **b,** in nitrostyrene-bound M45Y/F50A (PDB code – 5CLO). The $2F_o$ - F_c electron density maps have been depicted as a grey mesh and contoured at 1.0 σ. Protein residues and *trans*-β-nitrostyrene are shown as sticks. The apostrophes in the labels denote that the residues are from neighbouring chains. Two binding modes of *trans*-β-nitrostyrene are shown (using different colors) which fit the electron density equally well.

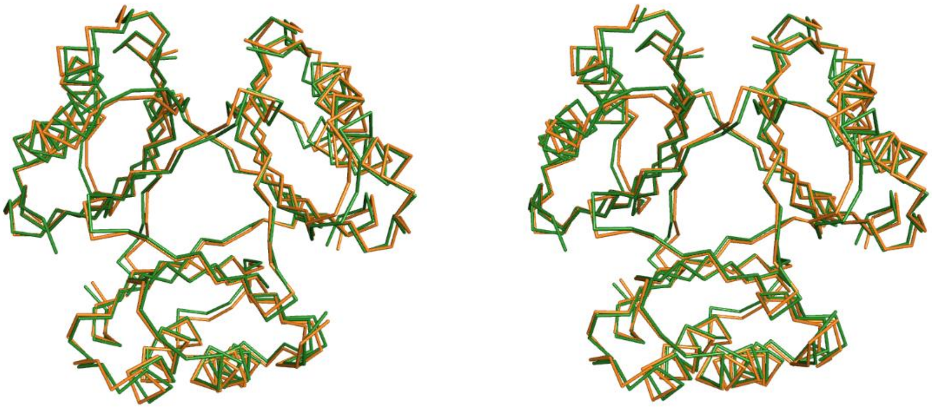

Supplementary Figure 30 | Stereo view of Cα-backbone superpositions of wild-type 4-OT and mutant M45Y/F50A. Wild-type 4-OT is depicted in orange whereas the mutant enzyme is shown in green.

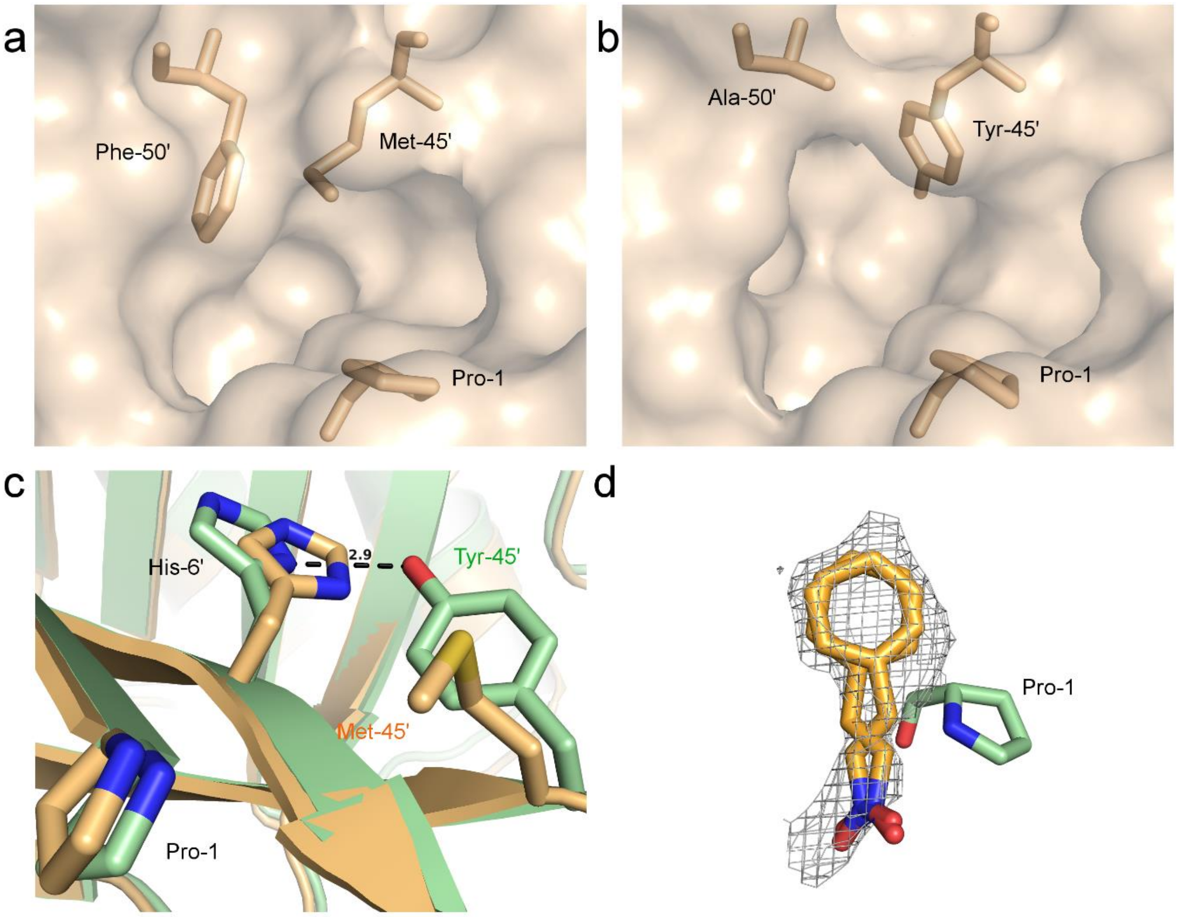

Supplementary Figure 31 | Surface representation of the Pro-1 pocket in **a,** wild-type 4-OT and **b,** M45Y/F50A. **c,** Close-up view of the active site showing the orientation of Tyr-45 in the mutant M45Y/F50A. The hydrogen bonding interaction is shown with a black dashed line and the distance is in Å. The M45Y/F50A structure is shown in green and the wild-type structure in orange. **d,** The two conformations of *trans*-β-nitrostyrene (**5a**), in the M45Y/F50A structure, fitting the electron density map. The grey mesh portrays the composite omit $2F_o - F_c$ map at 2.3 Å resolution (and contoured at 1.0 σ).

Supplementary Tables

Supplementary Table 1 | Optical rotations of the enzymatically produced **6a** and **7**

Biocatalyst	Optical rotation ($[\alpha]^{25}_{D}$)	Product	Abs. conf.[a]	e.r.[b]
WT 4-OT[c]	-6.7° (c= 0.78, $CHCl_3$)[c]	**6a**	*3S*	90 : 10[c]
A33D	-4.8° (c= 0.60, $CHCl_3$)	**6a**	*3S*	99 : 1
M45Y/F50A	+5.0° (c=1.3, $CHCl_3$)	**6a**	*3R*	94 : 6
WT 4-OT	+5.8° (c= 1.3, $CHCl_3$)	**7**	*2R3S*	57 : 43
R39E	+45.4° (c= 1.8, $CHCl_3$)	**7**	*2R3S*	95 : 5
H6M/A33E/F50V	-53.0° (c= 2.2, $CHCl_3$)	**7**	*2S3R*	77 : 23
M45Y/F50A	-73.2° (c= 2.2, $CHCl_3$)	**7**	*2S3R*	96 : 4

[a] The absolute configuration is of the major enantiomer. [b] The e.r. was determined using chiral phase HPLC, and the absolute configurations were assigned based on literature data and confirmed by optical rotations.[2, 5-7] [c] Data reported previously.[7]

Supplementary Table 2 | Peak integrations corresponding to the chromatograms in **Supplementary Figure 27**.

entry	Sample name	e.r. (*2R3S:2S3R*)
1[a]	Rac-**7**	50 : 50
2[b]	WT 4-OT	70 : 30

[a] Corresponding to the chromatogram of racemic **7** **(Supplementary Figure 27a**). [b] Corresponding to the chromatogram of enzymatically produced **7** (**Supplementary Figure 27b**).

Supplementary Table 3 | Michael-type addition reactions of **4** to **5a** catalysed by wild-type 4-OT and 4-OT variants

**Entry**	**Biocatalyst**	**Catalyst loading (mol%)**	**Reaction time (h)**[a]	**e.r. (*2R3S:2S3R*)**[b]
1	WT	0.7	o.n.	60 : 40
2	H6L/A33E/F50V	0.7	1.5	11 : 89
3	H6M/A33E/F50V	0.7	1	23 : 77
4	F50V	0.7	5	22 : 78

[a] Data obtained from **Supplementary Figure 28**. [b] The e.r. values were obtained with chiral phase HPLC analysis of samples of purified **7**, obtained from the same reactions as depicted in **Supplementary Figure 28.**

Supplementary Table 4 | The e.r. values of **7** and **6a** produced by 4-OT mutants with a selectivity towards *2R3S*-**7** and *3S*-**6a**

Biocatalyst	e.r. of *syn*-**7**[a] (*2R3S* : *2S3R*)	e.r. of **6a**[b] (*3S* : *3R*)
WT 4-OT	66 : 34	89 : 11
A33D	82 : 18	98 : 2
R39E	94 : 6	96 : 4
A57Y	84 : 16	84 : 16
R61M	86 : 14	88 : 12

All depicted e.r. values are obtained from experiments using purified biocatalysts. [a] The e.r. values were determined by HPLC analysis using a chiral stationary phase (see Methods). [b] The e.r. values of derivatised **6a** were determined by HPLC analysis using a chiral stationary phase (see Methods).

Supplementary Table 5 | The e.r. values of **7** and **6a** produced by 4-OT mutants with a selectivity towards *2S3R*-**7** and *3R*-**6a**

Biocatalyst	e.r. *syn*-**7**[b] (*2S3R* : *2R3S*)	e.r. of **6a**[c] (*3R* : *3S*)
H6I	92 : 8	79 : 21
R11I	75 : 25	53 : 47
M45H[a]	80 : 20	87 : 13
F50A	93 : 7	93 : 7
G54E	77 : 23	87 : 13

All depicted e.r. values are obtained from experiments using purified biocatalysts. [a] The mutant which had the best enantioselectivity towards *2S3R*-**7** at position Met-45 was the Tyr mutant. However, this mutant could not be purified as it expressed poorly. Therefore, M45H was used instead, as it was the second-best mutant at this residue position in terms of enantioselectivity, based on the data from the mutability landscape. [b] The e.r. values were determined by HPLC analysis using a chiral stationary phase (see Methods). [c] The e.r. values of derivatised **6a** were determined by HPLC analysis using a chiral stationary phase (see Methods).

Supplementary Table 6 | The e.r. values of product **7** produced by double and triple mutants of 4-OT

Entry	Mutant[a]	e.r. (*2R3S:2S3R*)[b]
1	H6I/M45Y/F50A	4 : 96
2	M45H/F50A	2 : 98
3	M45Y/F50A	1 : 99
4	H6I/F50A	4 : 96

[a] The other double and triple mutants (H6I/M45H/F50A, H6I/M45Y and H6I/M45H) did not have any detectable 'Michaelase' activity. [b] The e.r. values were determined by HPLC analysis using a chiral stationary phase (see Methods).

Supplementary Table 7 | Crystallographic data collection and refinement statistics

	**M45Y/F50A**	**M45Y/F50A-Nitrostyrene**
Data collection		
Space group	C2	P1
Unit cell dimensions,		
a, *b*, *c* (Å)	87.1, 87.2, 97.2	56.7, 61.1, 75.8,
α, β, γ (°)	90.0, 94.5, 90.0	98.2, 95.1, 91.1
Resolution range (Å)	57 – 2.7	56 – 2.3
	(2.84 – 2.71) *	(2.38 – 2.30)
R_{merge}	0.148 (0.714)	0.074 (0.414)
Completeness (%)	99.4 (95.8)	94.7 (93.8)
Average I / σI	6.9 (1.8)	10.7 (2.0)
Redundancy	4.2	2.0
Refinement		
Resolution range	57 - 2.7	44 - 2.3
Nr of reflections	18204	42246
R_{work} / R_{free}	0.232/0.263	0.236/0.270
Composition of asymmetric unit (AU)	12 chains	18 chains
total number of atoms	5222	8027
solvent molecules	50	244
others	--	1 *trans*-β-nitrostyrene
Average B (Å^2)		
protein	40.7	33.7
ligand	---	41.9
solvent molecules	32.4	28.7
R.m.s.d bonds (Å) /angles (˚)	0.005/ 0.988	0.004/ 0.876
Ramachandran plot		
% most favoured	97.4	98.2
% allowed	2.6	1.8
PDB code	5CLN	5CLO

* Highest resolution shell values are shown in parentheses. Single crystals of the YA mutant and of the mutant crystallised in the presence of nitrostyrene were used to collect data sets for structure determination.

Supplementary Table 8 | The sequence of the used mutagenic primers

Entry	Name	Sequence 5′-3′
**1**	Rev. WT 4-OT	ATGTTATGGATCCTCAGCGTCTGACCTTGCTGGCCAGTTCGCCGCCGATGCCGAAGTGGCCCTT
**2**	Rev. F50A	ATGTTATGGATCCTCAGCGTCTGACCTTGCTGGCCAGTTCGCCGCCGATGCC**GGC**GTGGCCCTT
**3**	Rev. 45NNK	ATGTTATGGATCCTCAGCGTCTGACCTTGCTGGCCAGTTCGCCGCCGATGCCGAAGTGGCCCTTGGC**MNN**CTCCGT
**4**	Rev. F50A/45NNK	ATGTTATGGATCCTCAGCGTCTGACCTTGCTGGCCAGTTCGCCGCCGATGCC**GGC**GTGGCCCTTGGC**MNN**CTCCGT
**5**	Rev. F50V/45NNK	ATGTTATGGATCCTCAGCGTCTGACCTTGCTGGCCAGTTCGCCGCCGATGCC**GAC**GTGGCCCTTGGC**MNN**CTCCGT
**6**	Rev. F50L/45NNK	ATGTTATGGATCCTCAGCGTCTGACCTTGCTGGCCAGTTCGCCGCCGATGCC**CAG**GTGGCCCTTGGC**MNN**CTCCGT
**7**	Fwd. WT 4-OT	ATAGCAGGTACATATGCCTATTGCCCAGATCCACAT
**8**	Fwd. H6I	ATAGCAGGTACATATGCCTATTGCCCAGATC**ATT**ATCCTTGAAGGC
**9**	Fwd. H6L	ATAGCAGGTACATATGCCTATTGCCCAGATC**CTT**ATCCTTGAAGGC
**10**	Fwd. H6M	ATAGCAGGTACATATGCCTATTGCCCAGATC**ATG**ATCCTTGAAGGC
**11**	Fwd. H6V	ATAGCAGGTACATATGCCTATTGCCCAGATC**GTC**ATCCTTGAAGGC

Mutagenic codons are underlined and in bold, *Nde*I restriction sites (CATATG) are represented with a dashed underline and *BamH*I restriction sites (GGATCC) are represented with a wave underline.

Supplementary Table 9 | Summary of the reaction conditions for the semi-preparative scale synthesis of γ-nitroaldehydes **6b-g**.

Product	[Nitroalkene] (mM)	[Acetaldehyde] (mM)	Mol% 4-OT[a]	Co-solvent (% v/v)	Reaction volume (ml)
**6b**	1.3	65	2.8	DMSO 45%	50
**6c**	2.0	50	1.5	DMSO 40%	60
**6d**	2.0	50	0.5	EtOH 10%	60
**6e**	2.0	50	1.8	EtOH 10%	60
**6f**	2.0	50	3.7	DMSO 40%	60
**6g**	3.0	150	5.3	DMSO 5%	12.8

These reaction conditions were based on earlier reported optimized reaction conditions.[7, 8] [a] Relative to the nitroalkene.

Supplementary Discussion

Engineering 4-OT variants with further enhanced 'Michaelase' activity

To investigate whether the positions at which single mutations improved the activity of 4-OT in the Michael-type addition of **4** to **5a** (i.e. His-6, Ala-33, Met-45 and Phe-50) are good targets to further enhance 4-OT's promiscuous 'Michaelase' activity, these 'hotspot' positions were subjected to combinatorial mutagenesis (see Methods). The resulting library was screened for Michael-type addition activity of **3** or **4** to **5a**. As might be expected from the mutability landscape shown in **Fig. 3b** (main text), this library did not contain any mutants which were more active in the Michael-type addition of **3** to **5a** than the single mutant A33D. On the other hand, screening of the library resulted in the identification of two triple mutants (H6M/A33E/F50V and H6L/A33E/F50V) with pronounced activity for the Michael-type addition of **4** to **5a**. Surprisingly, these mutants did not have a mutation at position Met-45, even though this position was randomized to all possible amino acids. This may suggest that mutations at position Met-45 do not contribute to the favourable effect of the mutations at the other positions in either an additive or synergistic manner. The progress curves of the Michael-type addition of **4** to **5a** catalyzed by the purified enzymes clearly demonstrate the strongly enhanced activities of these triple mutants when compared to wild-type 4-OT and the best single mutant F50V (**Supplementary Figure 28**).

The synthetic potential of the best triple mutant, H6M/A33E/F50V, was further compared to that of wild-type 4-OT using 0.7 mol% enzyme and a 25-fold excess of **4** (50 mM) over **5a** (2 mM). Under these optimized conditions, the triple mutant is more efficient in the addition of **4** to **5a** than wild-type 4-OT, with the H6M/A33E/F50V-catalyzed reaction being completed within 1 h at room temperature (**Fig. 4b**). Product analysis (**Supplementary Figures 7 and 8**) revealed that mutant H6M/A33E/F50V exhibits inverted enantioselectivity (compared to wild-type 4-OT) for the Michael-type addition of **4** to **5a**, producing the *2S3R* enantiomer of 2-ethyl-4-nitro-3-

phenylbutanal (**7**) with an e.r. of 77:23 (**Table 1, entry 8**). Thus, combinatorial mutagenesis of the identified 'hotspot' positions indeed yielded a highly active biocatalyst for the Michael-type addition of **4** to **5a**, yielding enantioenriched γ-nitroaldehyde **7**.

Supplementary References

1. Gotoh, H., Ishikawa, H. & Hayashi, Y. Diphenylprolinol silyl ether as catalyst of an asymmetric, catalytic, and direct Michael reaction of nitroalkanes with alpha,beta-unsaturated aldehydes. *Org. Lett.* **9**, 5307-5309 (2007).

2. Betancort, J. M. & Barbas, C. F.,. Catalytic direct asymmetric Michael reactions: taming naked aldehyde donors. *Org. Lett.* **3**, 3737-3740 (2001).

3. Patora-Komisarska, K., Benohoud, M., Ishikawa, H., Seebach, D. & Hayashi, Y. Organocatalyzed Michael addition of aldehydes to nitro alkenes - Generally accepted mechanism revisited and revised. *Helv. Chim. Acta* **94**, 719-745 (2011).

4. Purkarthofer, T. *et al*. A biocatalytic Henry reaction--the hydroxynitrile lyase from Hevea brasiliensis also catalyzes nitroaldol reactions. *Angew. Chem. Int. Ed. Engl.* **45**, 3454-3456 (2006).

5. Alexakis, A. & Andrey, O. Diamine-catalyzed asymmetric Michael additions of aldehydes and ketones to nitrostyrene. *Org. Lett.* **4**, 3611-3614 (2002).

6. Miao, Y., Geertsema, E. M., Tepper, P. G., Zandvoort, E. & Poelarends, G. J. Promiscuous catalysis of asymmetric Michael-type additions of linear aldehydes to beta-nitrostyrene by the proline-based enzyme 4-oxalocrotonate tautomerase. *ChemBioChem.* **14**, 191-194 (2013).

7. Geertsema, E. M. *et al*. Biocatalytic Michael-type additions of acetaldehyde to nitroolefins with the proline-based enzyme 4-oxalocrotonate tautomerase yielding enantioenriched gamma-nitroaldehydes. *Chem. Eur. J.* **19**, 14407-14410 (2013).

8. Zandvoort, E., Geertsema, E. M., Baas, B. J., Quax, W. J. & Poelarends, G. J. Bridging between organocatalysis and biocatalysis: asymmetric addition of acetaldehyde to beta-nitrostyrenes catalyzed by a promiscuous proline-based tautomerase. *Angew. Chem. Int. Ed. Engl.* **51**, 1240-1243 (2012).
